# The goose genome sequence leads to insights into the evolution of waterfowl and susceptibility to fatty liver

**DOI:** 10.1186/s13059-015-0652-y

**Published:** 2015-05-06

**Authors:** Lizhi Lu, Yan Chen, Zhuo Wang, Xiaofeng Li, Weihu Chen, Zhengrong Tao, Junda Shen, Yong Tian, Deqian Wang, Guoqin Li, Li Chen, Fang Chen, Dongming Fang, Lili Yu, Yudong Sun, Yong Ma, Jinjun Li, Jun Wang

**Affiliations:** Institute of Animal Husbandry and Veterinary Science, Zhejiang Academy of Agricultural Sciences, Hangzhou, China; BGI-Shenzhen, Shenzhen, 518083 China; Institute of Zhedong White Goose, Xianshan, China; BGI-Tech, BGI-Shenzhen, Shenzhen, 518083 China; Department of Biology, University of Copenhagen, Copenhagen, Denmark; King Abdulaziz University, Jeddah, Saudi Arabia

## Abstract

**Background:**

Geese were domesticated over 6,000 years ago, making them one of the first domesticated poultry. Geese are capable of rapid growth, disease resistance, and high liver lipid storage capacity, and can be easily fed coarse fodder. Here, we sequence and analyze the whole-genome sequence of an economically important goose breed in China and compare it with that of terrestrial bird species.

**Results:**

A draft sequence of the whole-goose genome was obtained by shotgun sequencing, and 16,150 protein-coding genes were predicted. Comparative genomics indicate that significant differences occur between the goose genome and that of other terrestrial bird species, particularly regarding major histocompatibility complex, Myxovirus resistance, Retinoic acid-inducible gene I, and other genes related to disease resistance in geese. In addition, analysis of transcriptome data further reveals a potential molecular mechanism involved in the susceptibility of geese to fatty liver disease and its associated symptoms, including high levels of unsaturated fatty acids and low levels of cholesterol. The results of this study show that deletion of the goose *lep* gene might be the result of positive selection, thus allowing the liver to adopt energy storage mechanisms for long-distance migration.

**Conclusions:**

This is the first report describing the complete goose genome sequence and contributes to genomic resources available for studying aquatic birds. The findings in this study are useful not only for genetic breeding programs, but also for studying lipid metabolism disorders.

**Electronic supplementary material:**

The online version of this article (doi:10.1186/s13059-015-0652-y) contains supplementary material, which is available to authorized users.

## Background

Geese play an important role in agricultural economics, with China producing the vast majority (94%) of the approximately 2.23 million tons of goose meat consumed worldwide annually, followed by Egypt, Hungary, and Poland [1]. Compared with other terrestrial poultry (for example, chicken and turkey), waterfowl, such as ducks and geese possess uniquely favorable economic traits. First, they exhibit a low susceptibility to certain avian viruses, showing little or no symptoms while still acting as a virus carrier, making them a natural repository for certain avian viruses [2-4]. Second, compared to other birds, the goose liver has a high capacity for fat accumulation, although geese do not normally develop liver fibrosis or necrosis. In agricultural production, this particular phenotype is manifested following short-term overfeeding (approximately 2 to 3 weeks), resulting in fatty livers and a 5- to 10-fold increase in liver size [5]. Previous studies have shown that the serum enzyme levels of overfed geese are similar to those observed in humans with non-alcoholic fatty liver disease [5-7], suggesting that the unique fat storage and metabolic characteristics of goose liver may be an important reference for the study of lipid metabolism disorders in humans.

In order to determine special characteristics of geese, we sequenced and analyzed the complete goose genome. The results of this study may be useful for genetic breeding programs with geese and other waterfowls, and may serve as an important reference for the study of lipid metabolism disorders in humans.

## Results and discussion

### Genome assembly and annotation

We sequenced an individual *Anser cygnoides* genome using an Illumina HiSeq-2000 instrument, obtaining approximately 139.55 Gb with small-insert-size libraries (200 bp, 500 bp, or 800 bp; average read length: 100 bp) and large-insert-size libraries (2 kb, 5 kb, 10 kb, or 20 kb; average read length: 49 bp; Additional file 1: Table S1). Sequence data were assembled into a 1.12-Gb draft genome using SOAPdenovo software (Table 1). Our assembly covered >98% of the transcriptome-assembled unigenes (Additional file 1: Table S2), indicating that the genome sequence was of high quality. The average GC content of the goose genome is approximately 38%, similar to that of other birds such as chicken, duck, turkey, and zebra finch (Additional file 2: Figure S1). By combining homology-based, *ab initio* prediction and transcriptome-assisted methods, we predicted 16,150 genes (Additional file 1: Table S3), 75.7% of which are supported by homology-based evidence (Additional file 1: Table S4), and 77.7% are covered by transcriptome reads (Table 1). We found that 77.7% of the identified genes were well supported by public protein databases (Additional file 1: Table S5). The repeat content of the goose genome is similar to that of chicken, duck, turkey, and zebra finch (Additional file 1: Table S6). We also predicted 153 microRNAs (miRNAs), 69 rRNAs, 226 tRNAs, and 206 small nuclear RNAs (snRNAs) in the goose genome (Additional file 1: Table S7).

**Table 1:** **Assembly and annotation statistics for the goose genome**

**Features**	
Estimate of genome size	1,208,661,181 bp
Number of scaffolds (≥2 kb)	1,049
Total size of assembled scaffolds	1,122,178,121 bp
N50 (scaffolds)	5.2 Mb
Longest scaffold	24 Mb
Number of contigs (≥2 kb)	60,979
Total size of assembled contigs	1,086,838,604 bp
N50 (contigs)	27.5 Kb
Longest contig	201 Kb
GC content	38%
Number of gene models	16,150
Total size of repeats	71,056,681 bp
Repeats share in genome	6.33%
Supported by RNA-Seq data	77.7%

### Comparative genomic analysis

We compared genome synteny and orthologous relationships among bird genomes. The goose genome has a high synteny with the duck genome [8], which covered approximately 81.09% and 82.35% of each genome, respectively (Additional file 1: Table S8 and Additional file 2: Figure S2), whereas approximately 592 goose scaffolds with lengths >5 kb mapped to and occupied 67.67% of the chicken genome [9] (Additional file 1: Table S8 and Additional file 2: Figure S3). In addition, we found that chromosomal rearrangements occur between the goose and chicken genomes (Additional file 1: Tables S9 and S10 and Additional file 2: Figure S4). For example, scaffold 45 is a goose genome sequence fragment, but it was in synteny with chromosomes 4 and 5 of the chicken genome. When comparing orthologs, 70% of the goose genes corresponded with 1:1 orthologs in the chicken gene-set (Additional file 2: Figure S5). Of the 1:1 orthologs for goose vs. duck (8,322 orthologs), however, 26.62% share up to 90% identity (Additional file 2: Figure S5). For chicken vs. turkey, 48.33% of the 1:1 orthologs (9,378 orthologs) share up to 90% identity (Additional file 2: Figure S5). For peregrine vs. saker, 57.87% of the 1:1 orthologs (10,569 orthologs) share up to 90% identity (Additional file 2: Figure S5).

**Figure 1:**
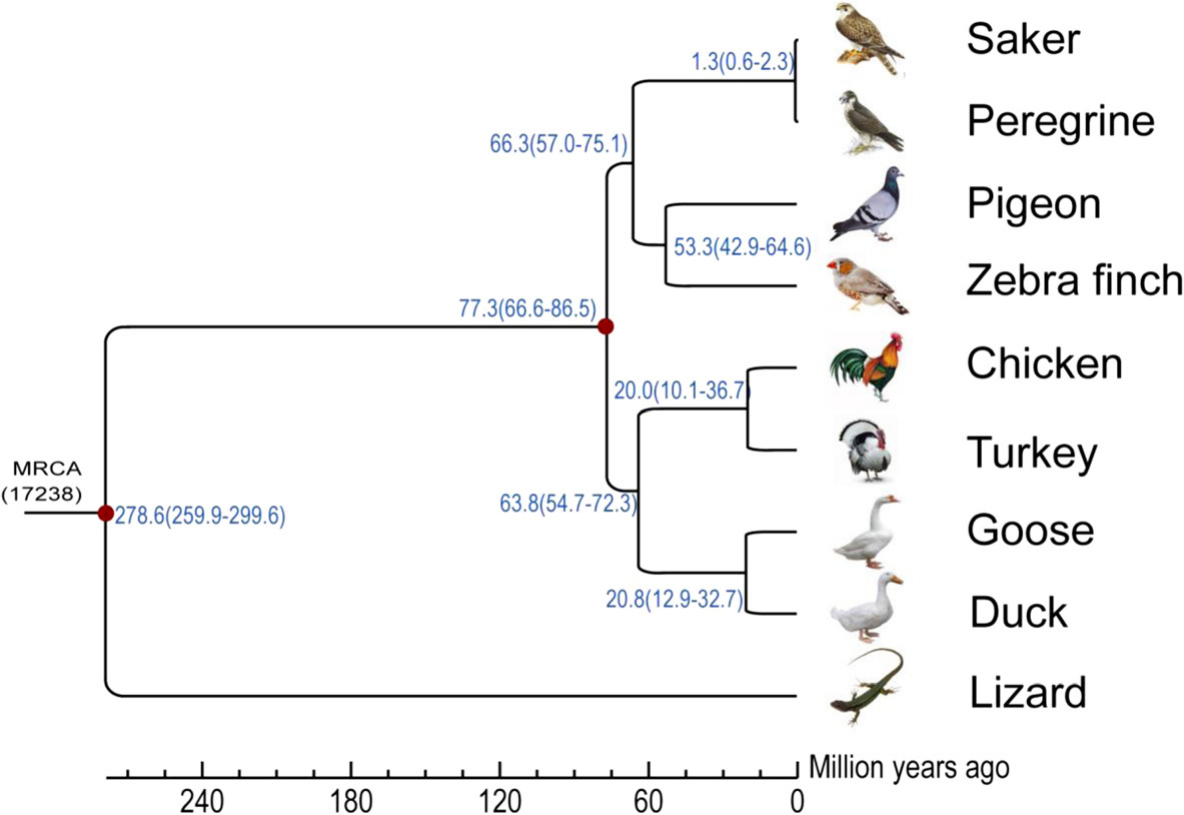
Divergence times for the nine species investigated in this study. A phylogenetic tree based on 4-fold degenerate sites in single-copy orthologous genes is shown. The divergence time estimates were calibrated using fossil data for lizard-bird and chicken-zebra finch. The estimated divergence times and associated 95% CIs are shown.

A phylogenetic tree of eight avian species (goose, duck, chicken, turkey, zebra finch, pigeon, peregrine, and saker) was constructed using 4-fold degenerate sites from 5,081 single-copy orthologs. Analysis of the resulting tree revealed that geese and ducks belong to a subclade that was most likely derived from a common ancestor approximately 20.8 million years ago (Mya), whereas the chicken and turkey diverged 20.0 Mya, and the peregrine and saker diverged 1.3 Mya (Figure 1 and Additional file 2: Figure S6). Of the nine species, goose-specific gene families (other species lack these families) have enriched gene ontology (GO) functions, such as zinc ion binding, integrase activity, and DNA integration. Moreover, the olfactory receptor activity, DNA metabolic processing, G-protein coupled receptor activity, and transmembrane receptor activity GO categories exhibit the most significant gene-family expansion when compared with others birds (Additional file 1: Table S11), indicating that these function were enhanced during goose evolution.

### Rapidly and slowly evolved GO terms

To identify the GO categories that have undergone rapid or slow evolution in waterfowl, we compared two waterfowl (goose and duck) with terrestrial birds (chicken and turkey). We searched for functionally related genes with exceptionally high or low selection constraints in the goose and duck. For categories with at least 10 genes, the ω value (ω = Ka/Ks, where Ka = number of non-synonymous substitutions per non-synonymous site, and Ks = number of synonymous substitutions per synonymous site) was calculated for these categories and normalized using the median ω of each species pair. We identified 191 GO categories with elevated Ka/Ks ratios at the specified threshold between the waterfowl and terrestrial birds (Additional file 1: Table S12). Nineteen of these GO categories, including GTPase activity, galactosyltransferase activity, chloride transport, and GABA-A receptor activity may have undergone significantly rapid evolution (Additional file 1: Table S12).

### Positive selection

Ortholog identification was performed for goose, duck, zebra finch, chicken, turkey, and pigeon genome sequences, using the method applied for accelerated GO category analysis. Alignments of 7,861 orthologous genes were used to estimate the ratio of the rates of non-synonymous and synonymous substitutions per gene (ω), using the Codeml program under a branch-site model and F3x4 codon frequencies. We then performed a likelihood ratio test and identified 21 positively selected genes (PSGs) in waterfowl branches by means of FDR adjustment with Q-values <0.05 (Additional file 1: Table S13). Several of the PSGs, including eIF-3S1, GATA1, and eIF-3A, are involved in transcription or translation regulation. Kinase (PIK3R, FGFR2) and signaling molecule (KAI1) genes were also under positive selection, indicating that they may be involved in adaptation to an aquatic environment (Additional file 1: Table S13).

### The resistance of waterfowl to disease

The major histocompatibility complex (MHC) gene is widely expressed in jawed vertebrates, and its function correlates with host disease resistance and immune responses [10-12]. Transposable elements in the chicken MHC region are more prevalent compared to the goose MHC region (54.62% in chicken vs. 15.11% in goose; Additional file 1: Table S14). Moreover, the distribution of the goose and chicken MHC region is different (Additional file 1: Table S15 and Additional file 2: Figure S7). In addition, we found that the goose genome exhibits substantial copy-number variations of innate immune response-related genes, as well as gene structures, when compared with chicken, turkey, zebra finch, human, and rat genomes (Additional file 1: Table S16). RNA viruses that escape toll-like receptors and infiltrate the cytoplasm are recognized by Retinoic acid-inducible gene I (RIG-I), a pattern-recognition receptor that plays an important antiviral role [13-16]. Results from recent studies have shown that RIG-I is present in most mammals and some birds [17-19]. We found that RIG-I genes aligned well between goose and zebra finch (Additional file 1: Tables S17 and S18), but only fragments of the goose RIG-I aligned with the chicken and turkey RIG-I genes (Additional file 1: Table S19). We constructed a phylogenetic tree based on these data (Additional file 2: Figures S8 and S9) and found that the RIG-I gene is absent in chickens and turkeys. Compared to turkeys and chickens, some mammal and waterfowl species have increased resistance to the influenza virus [20,21]. This phenomenon may be because most mammals have two Myxovirus resistance (Mx) genes, while avian birds have only one. The Mx gene is a member of the guanine-3 phosphokinase gene family, and its expression is induced by interferons [21]. Many Mx proteins have been shown to provide influenza virus resistance at the cellular level [22]. Moreover, the different Mx proteins confer resistance to different diseases, and single base mutations can affect the ability of the protein to confer resistance [21,22]. In addition, the phylogenetic tree shows that mutations at key sites in the chicken and turkey Mx genes may inactivate the Mx protein, affecting antiviral activity and leading to diminished viral resistance (Additional file 2: Figures S10 and S11).

### The susceptibility of geese to fatty liver

**Figure 2:**
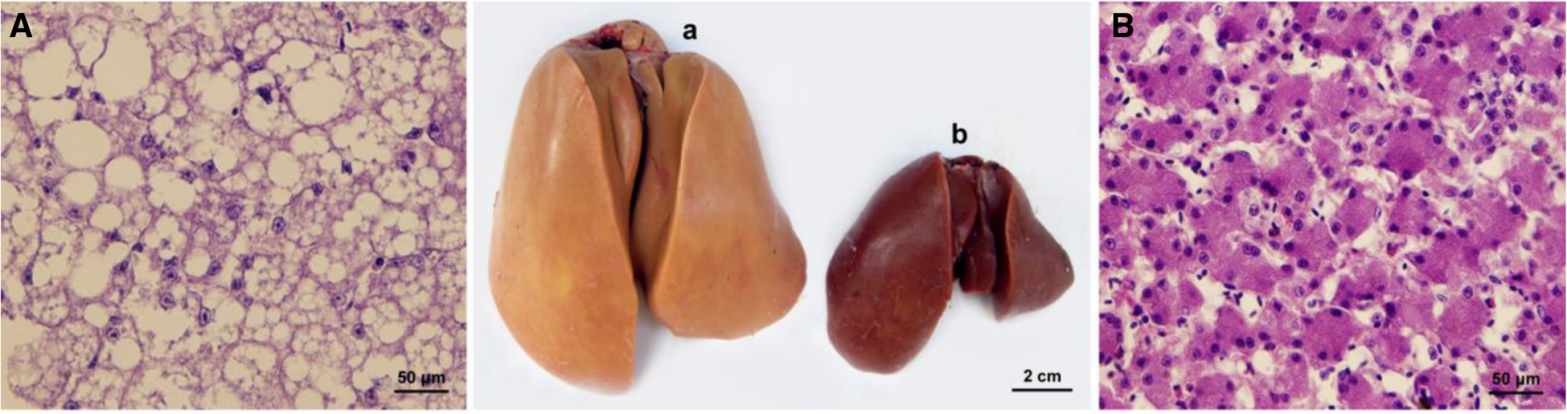
Comparison of livers and liver tissue sections between overfed and control geese. **(A)** Goose liver tissue section after 3 weeks of overfeeding (200×); (a) Goose liver after 3 weeks of overfeeding. **(B)** Normal goose liver tissue section (200×); (b) Normal goose liver.

**Figure 3:**
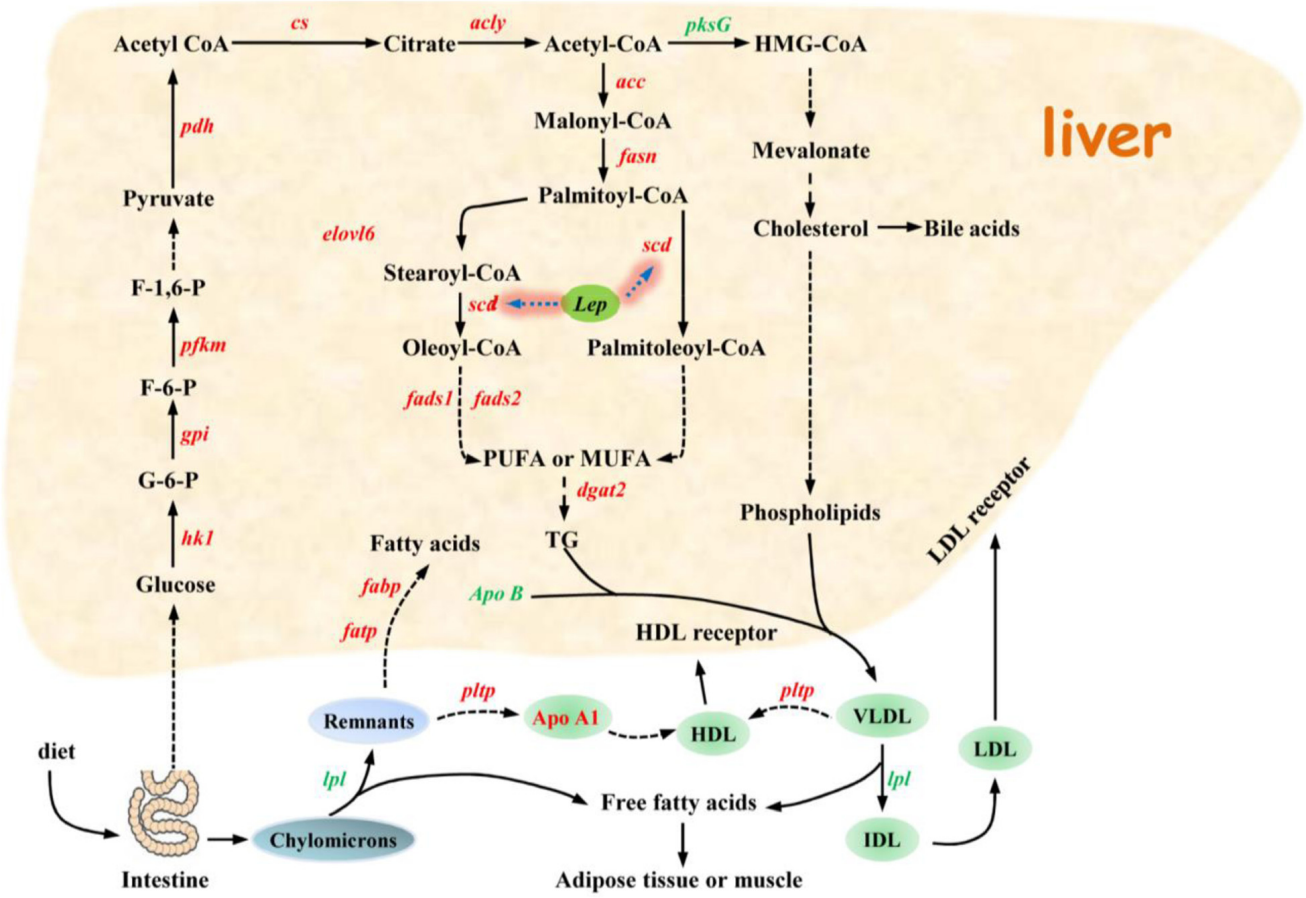
Glucose and lipid metabolic pathways in goose liver. The diagram shown is based on established KEGG pathways and hepatic lipid-metabolism findings from previous studies. The solid lines represent single-step reactions, whereas the dotted lines indicate multi-step reactions. Red and green italic letters represent increased and decreased liver gene expression levels, respectively, when comparing a goose overfed a carbohydrate-rich diet vs. the control group fed a normal diet. Gene symbols: acc, acetyl-Coenzyme A carboxylase; acly, ATP citrate lyase; apoB, apolipoprotein B; cs, citrate synthase; dgat2, diacylglycerol O-acyltransferase 2; elovl6, elongation of very long chain fatty acids protein 6; fads1, fatty acid desaturase 1; fads2, fatty acid desaturase 2; fasn, fatty acid synthase; gpi, glucose-6-phosphate isomerase; hk1, hexokinase 1; lep, leptin; lpl, lipoprotein lipase; pdh, pyruvate dehydrogenase; pfkm, phosphofructokinase; scd, stearoyl-CoA desaturase; fatp, fatty acid transporter protein; pksG, hydroxymethylglutaryl-CoA synthase.

The liver is a vital organ that plays an important role in lipid metabolism, digestion, absorption, synthesis, decomposition, and transport. Under natural conditions, birds, especially some wild waterfowl, are more likely to show non-pathological hepatic steatosis as a result of energy storage before migration [23]. To identify the genetic mechanism underlying the occurrence of fatty liver, many previous studies have focused on goose fatty liver formation [5-7,24,25]. However, to date, the adaptive molecular mechanisms that induce higher synthesis of hepatic lipids, especially unsaturated fatty acids, in response to carbohydrate-rich diets remain to be understood in waterfowl species. To establish the molecular mechanism responsible for fat deposition in goose liver, we analyzed goose liver tissues in terms of cell morphology and plasma parameters, as well as performed tissue transcriptome and microRNA sequencing and analysis. After 20 d of overfeeding, the body weights of overfed geese were significantly higher than that of control geese. Liver weights were considerably higher in overfed geese (*P* <0.01) and accounted for 8.44% of the overall body weight, compared with 3.26% in the control geese (Additional file 1: Table S20). During the force-feeding period, overfeeding significantly increased the glucose, total cholesterol (TC), triglyceride (TG), and free fatty acid serum concentrations (Additional file 1: Table S21). Figure 2 shows that overfeeding of geese with a high-energy diet resulted in liver enlargement, with several lipid droplets deposited in the liver cells. Transcriptome analysis showed that the gene expression levels of key enzymes involved in hepatocyte fatty acid synthesis (*hk1*, *gpi*, *pfkm*, *pdh*, *cs*, *acly*, *mdh1*, *me1*, *acc*, *fasn*, *elovl6*, *scd*, *fads1*, *fads2*, and *dgat2*) were significantly elevated (red italic lettering shown in Figure 3 and Table 2), while the activities of extracellular liver lipoprotein lipase (*lpl*) and the first key enzyme (*pksG*) involved in hepatic cholesterol synthesis were significantly reduced (green italic lettering in Figure 3 and Table 2). The expression of fatty acid transport protein genes (*fatp*), which are responsible for the transport of exogenous lipids into cells [26], was significantly increased (Figure 3 and Table 2). In contrast, expression of apolipoprotein B (*apoB*), which is responsible for binding with endogenous lipids and promoting their diffusion from liver cell membranes as very low-density lipoproteins (VLDLs) [27,28], was significantly attenuated (Figure 3 and Table 2). Previous studies have shown that *lpl* plays a major role in lipolysis of fatty acids from extracellular chylomicrons or VLDL, which can then be used or deposited in fat or muscle tissues [7,23]. The reduction in *lpl* activity increases the tendency for a large amount of extracellular lipids to diffuse into liver cells. These results suggest that the mechanism of goose fatty liver formation is mainly attributable to an imbalance between the storage and secretion (as plasma lipoproteins) of newly synthesized endogenous lipids and exogenous lipids in the cytoplasm. The liver lipid secretion capacity cannot offset the storage of newly synthesized cytoplasmic lipids, resulting in fat deposition in the liver.

**Table 2:** **Information on the expression of glucolipid metabolism-related genes in goose liver**

**Name**	**C-1A-expression**	**T-1A-expression**	**C-1A-RPKM**	**T-1A-RPKM**	**log2Ratio**	**Up-/Down regulation**	***P*** **value**	**FDR**
Lpl	781	91	30.87285963	4.089769539	-2.916247667	Down	2.68E-119	1.06E-117
Fasn	81651	414355	509.3386174	2938.659719	2.528461303	Up	0	0
fads2	3679	8407	231.481134	601.393154	1.377413828	Up	0	0
fads1	7505	17147	322.6220046	838.0367342	1.377168642	Up	0	0
pfkm	2780	9997	72.0097561	294.4068125	2.03154677	Up	0	0
elovl6	7719	23097	561.4417291	1909.98889	1.766356045	Up	0	0
dgat2	204	1358	11.44903316	86.65029289	2.919978854	Up	4.96E-243	3.64E-241
Gpi	4038	7274	164.6323252	337.1737168	1.034244447	Up	1.60E-306	1.56E-304
Lepr	279	258	3.697717665	3.887593702	0.072242375	Up	5.60E-01	6.88E-01
Pdh	3065	5848	120.1146982	260.558148	1.117192669	Up	9.39E-280	8.01E-278
Acc	17885	52933	129.0541753	434.2514158	1.750553735	Up	0	0
me1	539	3124	44.14885523	290.9198306	2.720173707	Up	0	0
mdh1	6693	14942	364.3829222	924.8632877	1.343784779	Up	0	0
pksG	45643	9198	1255.614662	287.6783778	-2.125865085	Down	0	0
acly	24637	55079	313.72077	797.3942774	1.345812205	Up	0	0
fatp	271	624	6.844164678	17.9170856	1.388389609	Up	1.23E-43	2.35E-42
apoB	292897	161550	1618.343461	1014.832473	-0.673276234	Down	0.00E + 00	0.00E + 00
Scd	11207	105759	494.6247139	5306.832681	3.423444832	Up	0	0
hk1	2342	11617	26.88532988	151.6191604	2.495561003	Up	0	0
Cs	1358	2599	14.68979424	31.96346415	1.121609586	Up	2.96E-126	1.23E-124

**Figure 4:**
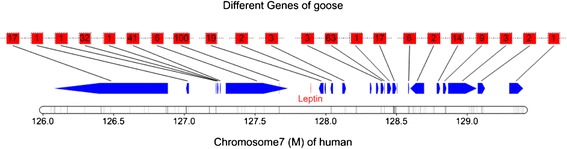
Proximal regions of the *lep* gene in *H. sapiens.* The figure shown depicts a region of *H. sapiens* chromosome 7 (126.0 to 129.4 Mb), for which the blue arrows indicate the orientation of genes along the chromosome. Arrows pointing to the right or left represent genes expressed from the positive or negative DNA strand, respectively. The relative position of the *H. sapiens LEP* gene is shown with red labeling. The black areas along the chromosome represent regions of co-linearity between *H. sapiens* and *A. cygnoides* that are generally considered to be conserved. The figure shows no co-linear regions near the *LEP* gene in *H. sapiens*. The dotted line represents *A. cygnoides* genes, which were distributed in different scaffolds, with the links determined based on the existence of homologous genes in adjacent regions of the *H. sapiens* chromosome. Numbers in the dashed boxes represent the number of homologous links of *H. sapiens* genes found in *A. cygnoides* (BLASTP: e value set to 1e^-5^). The gaps in the dashed line represent corresponding genes in *H. sapiens* that lacked homologous genes in *A. cygnoides*. The diagram shows that during the evolutionary divergence of *H. sapiens* and *A. cygnoides*, genomic fragments near the *lep* gene may have been deleted from the goose genome, with the *lep* gene excluded from further replication as a result.

In addition, we found that the copy numbers of some genes related to liver lipid synthesis and transportation were significantly greater than those in other species. For example, the goose has more than three times as many *scd* gene copies than that found in the *Gallus gallus*, *A. cygnoides*, and *Homo sapiens* genomes (Additional file 1: Table S22). The *Scd* gene is a key enzyme in the hepatic synthesis of monounsaturated fatty acids. Its gene expression is independently regulated by insulin and leptin, which exerts different regulatory effects: insulin promotes *scd* gene expression, whereas leptin plays an inhibitory role [29-32]. Through the Jak2, ERK1/2, and p90RSK signaling pathways, leptin can regulate the sp1 transcription factor downstream of the *scd* gene promoter to inhibit *scd* gene expression [33]. Moreover, some studies have reported that the loss or inhibition of SCD could be of benefit for the treatment of obesity, hepatic steatosis, and other metabolic disorders [24,34]. However, our study showed that *A. cygnoides* may not possess the *lep* gene (Figure 4 and Additional file 1: Table S23). The existence of *lep* in birds (especially domestic fowl) remains an elusive and controversial question [35,36], although *lep* sequences have been identified in some birds (Peregrine falcon, *F. peregrinus*, mallard, and zebra finch) [37]. In this study, we downloaded all known sequences of the *lep* gene as reference sequences for comparison with the goose genome. However, no similar fragments or reads were found that aligned to these genes. Considering that the GC content in birds is much higher than in mammals, it is possible that the *lep* gene is present in the goose genome, but that it resides in a region that was not sequenced. However, we were unable to clone this gene from the goose genome by PCR, after multiple attempts. Likewise, despite numerous large sequencing projects accruing more than 600 K EST sequences and the repeated assembly of the chicken genome sequence, the *lep* gene has not been identified in the chicken genome or that of two other domestic birds (ducks and turkeys) [37]. More effort should be dedicated to determining the presence or absence of the *lep* gene in future studies.

Results from previous studies have shown that the toxic and damaging effects of saturated fatty acids (SFAs) in the liver are significantly stronger than those of monounsaturated fatty acids (MUFAs) [38,39]. The implication of these findings is that the physiological transformation of SFA into MUFA by *scd* enzymes could alleviate the toxic effects of excessive liver exposure to SFA. Furthermore, the results of some studies have indicated that ob/ob mice (*lep*-deficient model mice) readily develop hepatic steatosis, but do not show spontaneous progression to steatohepatitis or liver fibrosis [40,41] because leptin is an essential mediator of hepatic fibrogenesis [41,42]. We therefore hypothesize that deletions of the goose *lep* gene may result from positive selection, thus allowing the liver to adapt energy storage mechanisms for long-distance migration, as observed in other wild birds [43]. In addition, our results indicated that microRNAs are closely related to goose liver lipid metabolism in that multiple genes related to lipid synthesis or transport (*lpl*, *fads1*, *pfkm*, *mdh1*, *pksG*, *fatp*, *acly*, *scd*, *cs*, and *elovl1*) are regulated by single or multiple microRNAs (Additional file 1: Table S24), although this requires further verification.

## Conclusions

In summary, this is the first report describing the complete goose-genome sequence and contributes to the genomic resources for studying aquatic birds. Genome-wide comparisons and orthologous analyses showed that the genome map is reliable and that the goose is a particularly interesting species with regard to evolutionary adaptation to its environment. The availability of the full goose-genome sequence will facilitate future genetic breeding programs. Moreover, studies examining goose genes involved in disease resistance and hepatic lipid metabolism may reveal unique immunity or disease-resistance mechanisms in waterfowls, and thus provide a valuable reference for research on human diseases related to lipid metabolism in the liver.

## Materials and methods

### Genome sequencing and assembly

High-quality genomic DNA was extracted from whole blood of a 70-day old male Zhedong goose (*A. cygnoides*) reared in Xianshan County, Zhejiang Province, China. We constructed 12 paired-end sequencing libraries for whole genome sequencing (WGS) using a WGS kit (Illumina), according to the manufacturer’s recommended protocol. We next sequenced the DNA on a HiSeq 2000 sequencing platform and assembled the short sequences using SOAPdenovo software [44]. The genome size was calculated from the total length of the sequence reads divided by the sequencing depth. To estimate the sequencing depth, we counted the frequency of each 17-mer from the WGS sequencing reads and plotted the copy-number distribution. The peak value of the frequency curve represented the overall sequencing depth. We used the algorithm, where k_num_ is the k-mer number, k_depth_ is the K-mer depth, b_num_ is the base number, and b_depth_ is the base depth. G denotes the genome size, and k_depth_ is the overall depth estimated from the K-mer distribution. To assess the completeness of the assembly, we aligned the unigenes from Illumina RNA-Seq data to the assembled sequence using the BLAT algorithm with default parameters.

### Repeat annotation

Repetitive sequences and transposable elements (TEs) in the genome were identified using a combination of *de novo* and homology-based approaches at both the DNA and protein levels. Briefly, we first constructed a *de novo* repeat library for *A. cygnoides* using RepeatModeler [45] with the default parameters, which generated consensus sequences and classification information for each repeat family. To identify transposable elements at the DNA level, RepeatMasker [46] was applied, using both the repetitive sequence database that we built and that deposited in Repbase [47]. We next executed protein-based RepeatMasking [48] in a WU-BLASTX search against the TE protein database to further identify repeat-related proteins. The overlapping TEs belonging to the same repeat class were collated and combined according to the coordination in the genome. In addition, we annotated tandem repeats using the Tandem Repeats Finder [46] (TRF) software. For comparisons with the *G. gallus* genome, we also annotated repetitive elements of the *G. gallus* genome using the same process and parameters. The repeat divergence rate was calculated as the percentage of substitutions in the corresponding regions between annotated repeats and consensus sequences in the Repbase database [49].

### Gene annotation

We conducted gene annotations for the *A. cygnoides* genome by combining homology information, *de novo* predictions, and RNA-Seq data. For the homology-based prediction, protein sequences obtained from four sequenced animal genomes, namely *G. gallus*, *H. sapiens*, *Meleagris gallopavo*, and *Taeniopygia guttata*, were mapped onto the *A. cygnoides* genome, using TBLASTN with an E-value cutoff of 1e^-5^. Homologous genome sequences were aligned against the matching proteins using GeneWise [50] for accurate spliced alignments. For *de novo* predictions, we performed Augustus [51] and GenScan [52] analysis of the repeat-masked genome, with parameters trained from the relative species, and filtered out partial sequences and/or small genes of <150 bp coding length. We next combined all the predictions using GLEAN [53] to produce consensus gene sets. Finally, we aligned all RNA reads to the reference genome, using TopHat [54], assembled the transcripts with Cufflinks [55] using the default parameters, and predicted the open reading frames (ORFs) to obtain reliable transcripts with HMM-based training parameters. To finalize the gene set, we combined the GLEAN set with the gene models produced by RNA-Seq, filtering out genes containing one exon that were only supported by the RNA-Seq data.

Gene functions were assigned based on the best matches derived from alignments with proteins annotated in the Swiss-Prot and TrEMBL [56] databases, using BLASTP (E-value ≤1e^-5^). We annotated motifs and domains using InterProScan [57], searching against publicly available databases, including ProDom, PRINTS, Pfam, SMART, PANTHER, and PROSITE. We also mapped the *A. cygnoides* genes to KEGG [58] pathway maps by searching the KEGG databases, identifying the best hit for each gene and then assigning them to the pathway maps.

tRNA genes were identified using tRNAscan-SE [59], with eukaryote parameters. For rRNA identification, we aligned the *H. sapiens* rRNA sequences against the *A. cygnoides* genome using BLASTN with an E-value cutoff of 1e^-5^. Subsequently, snRNAs were predicted using INFERNAL software [60] and searching against the Rfam database [61].

### Comparative genomics

We compared gene families from eight avian species (*A. cygnoides*, *Anas platyrhynchos*, *G. gallus*, *M. gallopavo*, *T. guttata*, *Columba livia*, *Falco peregrinus*, and *Falco cherrug*) and the green anole lizard (*A. carolinensis*) by TreeFam [62], using the following steps. Initially, protein sequence alignments were performed with Blastp with an E-value cutoff of <1e^-7^. HSP segments were then concatenated between the same pairs of proteins using the Solar software package, followed by the identification of homologous relationships between protein sequences, based on bit-scores and the identity of homologous gene pairs. Finally, gene families were detected by clustering using hcluster_sg [54], with a minimum edge weight >10, a minimum edge density >0.34, and with other default parameter values.

In phylogenetic analysis, echo single-copy family results from TreeFam were translated into amino acid sequences for multiple alignments by Muscle [63]. A phylogenetic tree of nine species was generated via super-alignment through the maximum-likelihood method in PhyML software [64] or the Bayesian inference method in MrBayes software [65] by concatenating all 4-fold degenerate sites of single-copy orthologs. The ages of speciation events were estimated using the Bayesian relaxed molecular clock (BRMC) approach implemented in the MCMCTREE program in the PAML package [64]. Both the correlated molecular clock and the JC69 models were used to estimate speciation events. The MCMC process of the PAML MCMCTREE program was run to sample 100,000 times, with the sampling frequency set to 2 after a burn-in of 10,000 iterations. The fine-tune parameters were set to allow acceptance proportions falling in intervals (0.15, 0.7). Elsewhere, the default parameters were used. Two independent runs were performed to check convergence.

LASTZ local alignment software was used to align sequences between two genomes. The self-alignment generated by LASTZ, and most LASTZ parameters were set by default. Prior to aligning, repeat sequences were masked, and the genome assembly was split into several small subfiles. The maximum simultaneous gap allowed during aligning was 100 bp. After the alignment, we extracted alignment blocks of >1 kb and >90% identity. These alignment blocks were predicted to be SDs. After removing the overlapping fragments, we obtained a non-redundant set of SDs.

We downloaded the relative character genes (MHC, Mx, and RIG-I) from NCBI and aligned them with goose and homolog species gene sets using BLASTP with an E-value cutoff of 1e^-5^. Next, according to the function, the description of the genes to ensure the copy number, we constructed a phylogenetic tree with PhyML and compared the gene structures of single-copy genes.

### Transcriptome sequencing and analysis of goose susceptibility to fatty liver

Twelve healthy male geese hatched on the same day were grown under natural conditions of light and temperature at ChangXing Glory Goose Industry Co., Ltd. After 90 days, they were randomly divided into two groups (n = 6 per group). The control group was given free access to a normal diet (2,800 kcal/kg, 150 g of protein/kg). The overfed group was fed a carbohydrate-rich diet (3,500 kcal/kg, 100 g of protein/kg, and 4.8 g of fat/kg) for four meals (300 g/meal) per day. All geese had free access to water at all times. At the age of 110 days, all geese were deprived of feed overnight, but provided water. On the following morning, the geese were sacrificed, both the body and liver weights of geese were weighed, and approximately 8 g samples liver tissue samples were isolated and stored at -70°C until RNA extraction. Individual blood samples were collected from geese in both the control and overfed group on 90 and 110 days of age. Sera were separated by centrifugation at 3,500 × *g* for 15 min and stored at -20°C until further biochemical analysis. Whole-plasma parameters such as glucose, TC, TG, high-density lipoprotein, VLDL, lipoprotein, phospholipids, and free fatty acid serum levels were determined using corresponding kits. The protocol for goose treatment was in accordance with Chinese legislation on animal experimentation. Total RNA was isolated from the livers, and RNA sequencing libraries were constructed using the Illumina mRNA-Seq Prep Kit. We then sequenced all libraries using an Illumina HiSeq 2000 instrument.

To determine gene expression levels, RNA-Seq reads from the control and overfed groups were mapped to the assembly, and the reads per kilobase per million mapped reads (RPKM) values were calculated for each predicted transcript. Next, we compared gene expression levels in the two libraries, defining genes as differentially expressed if they showed at least a 2-fold change in expression and an adjusted *P* value of <0.001 (based on the Poisson model).

MicroRNA (miRNA) expression levels between two samples were compared to identify differentially expressed miRNAs, using the following steps: (1) miRNA expression was normalized in the two samples (control and overfed) to determine the expression of transcripts per million reads. miRNA was normalized using the formula: normalized expression = (actual miRNA count/total count of clean reads) × 1,000,000. (2) Fold-changes and *P* values were calculated from the normalized expression levels, using the formula: fold-change = log_2_ (treatment/control). The rules for predicting target genes of novel miRNA were based on those suggested by Allen *et al.* [66] and Schwab *et al.* [67], namely: (1) No more than four mismatches were permitted between sRNA and target (G-U bases count as 0.5 mismatches). (2) No more than two adjacent mismatches were allowed in the miRNA/target duplex. (3) No adjacent mismatches in positions two to 12 of the miRNA/target duplex (5′ end of miRNA) were permitted. (4) No mismatches in positions 10 to 11 of miRNA/target duplex were permitted. (5) No more than 2.5 mismatches in positions one to 12 of the miRNA/target duplex (5′ end of miRNA) were permitted. (6) The minimum free energy (MFE) of the miRNA/target duplex should be ≥75% of the MFE of the miRNA bound to its perfect complement.

### Data access

Accession codes: The whole-genome shotgun project has been deposited in DDBJ/EMBL/GenBank nucleotide core database under the accession code AOGC00000000. The version described in this paper is the first version, AOGC00000000. All short-read data have been deposited in the Sequence Read Archive (SRA) under accession SRA062749. Raw sequence data of the transcriptome have been deposited in the SRA under accession codes SRA251539.

## Additional files

Additional file 1: Table S1. Statistics for raw data and clean data. **Table S2.** EST evaluation of the goose genome assembly. **Table S3.** General statistics for predicting protein-coding genes. **Table S4.** Summary of evidence from the GLEAN gene model. **Table S5.** Functional annotation statistics. **Table S6.** General statistics regarding repetitive genome sequences. **Table S7.** Non-coding RNA genes in the genome. **Table S8.** Aligned sequence lengths of *four birds*. **Table S9.** Intra-chromosomal and inter-chromosomal rearrangements in the goose and chicken genomes. **Table S10.** Detailed information regarding intra-chromosomal and inter-chromosomal rearrangement in goose and chicken chromosomes. **Table S11.** GO-enrichment profiles in the expanded goose gene family. **Table S12.** Rapidly and slowly evolved GO terms. **Table S13.** Positive-selection genes found in the goose and duck genomes. **Table S14.** MHC region differences between the goose, duck, and chicken genomes. **Table S15.** Gene alignment information for the goose and chicken genome in the MHC region. **Table S16.** Copy-number variations of innate immunity genes. **Table S17.** RIG-I gene alignment results for six species. **Table S18.** Alignment information for the goose, duck, and zebra-finch RIG-I genes. **Table S19.** Alignment information for RIG-I gene fragments of chicken and turkey. **Table S20.** Carcass traits of geese after overfeeding. **Table S21.** Plasma parameters of geese after overfeeding. **Table S22.** Copy-number variation of glucolipid metabolism-related genes in geese and other animals. **Table S23.** Comparison of the chicken, duck, goose, human, and mouse *lep* gene sequences. **Table S24.** miRNA information corresponding to glucolipid metabolism-related genes. Additional file 2: Figure S1. GC content distributions for various bird genomes. **Figure S2.** Venn diagram of orthologous genes among five species. **Figure S3.** Whole-genome alignment of goose and chicken sequences. **Figure S4.** Chromosomal rearrangement between geese and chickens. **Figure S5.** Orthologous information of nine genomes. **Figure S6.** Phylogenetic tree constructed with orthologous genes. **Figure S7.** Differences between the chicken and goose MHC gene regions. **Figure S8.** Phylogenetic tree of the RIG-I gene in various species. **Figure S9.** Comparison of RIG-I gene structures in seven avian species. **Figure S10.** Phylogenetic tree of the Mx gene in seven species and comparison of gene structures. **Figure S11.** Single amino-acid changes in the Mx gene in four birds.

## References

[1] Pingel H (2011). Waterfowl production for food security. Lohmann Information..

[2] Webster RG, Bean WJ, Gorman OT, Chambers TM, Kawaoka Y (1992). Evolution and ecology of influenza A viruses. Microbiol Rev..

[3] Murata S, Hayashi Y, Kato A, Isezaki M, Takasaki S, Onuma M (2012). Surveillance of Marek’s disease virus in migratory and sedentary birds in Hokkaido, Japan. Vet J..

[4] Murata S, Chang KS, Yamamoto Y, Okada T, Lee SI, Konnai S (2007). Detection of the virulent Marek’s disease virus genome from feather tips of wild geese in Japan and the Far East region of Russia. Arch Virol..

[5] Mourot J, Guy G, Peiniau P, Hermier D (2006). Effects of overfeeding on lipid synthesis, transport and storage in two breeds of geese differing in their capacity for fatty liver production. Anim Res..

[6] Hermier D, Salichon MR, Guy G, Peresson R (1999). Differential channelling of liver lipids in relation to susceptibility to hepatic steatosis in the goose. Poult Sci..

[7] Xu HY, Wang Y, Han CC, Jiang L, Zhuo WH, Ye JQ, Wang JW (2010). Estimation of lipoprotein-lipase activity (LPL) and other biochemical changes in two breeds of overfeeding geese. Asian-Australasian J Anim Sci..

[8] Huang Y, Li Y, Burt DW, Chen H, Zhang Y, Qian W (2013). The duck genome and transcriptome provide insight into an avian influenza virus reservoir species. Nat Genet..

[9] International Chicken Genome Sequencing C (2004). Sequence and comparative analysis of the chicken genome provide unique perspectives on vertebrate evolution. Nature.

[10] Shiina T, Shimizu S, Hosomichi K, Kohara S, Watanabe S, Hanzawa K (2004). Comparative genomic analysis of two avian (quail and chicken) MHC regions. J Immunol..

[11] Dalgaard T, Boving MK, Handberg K, Jensen KH, Norup LR, Juul-Madsen HR (2009). MHC expression on spleen lymphocyte subsets in genetically resistant and susceptible chickens infected with Marek’s disease virus. Viral Immunol..

[12] Dalgaard TS, Vitved L, Skjodt K, Thomsen B, Labouriau R, Jensen KH (2005). Molecular characterization of major histocompatibility complex class I (B-F) mRNA variants from chickens differing in resistance to Marek’s disease. Scand J Immunol..

[13] Yoneyama M, Fujita T (2007). RIG-I family RNA helicases: cytoplasmic sensor for antiviral innate immunity. Cytokine Growth Factor Rev..

[14] Barral PM, Sarkar D, Su ZZ, Barber GN, DeSalle R, Racaniello VR (2009). Functions of the cytoplasmic RNA sensors RIG-I and MDA-5: key regulators of innate immunity. Pharmacol Ther..

[15] Cowled C, Baker ML, Zhou P, Tachedjian M, Wang LF (2012). Molecular characterisation of RIG-I-like helicases in the black flying fox, Pteropus alecto. Dev Comp Immunol..

[16] Schmidt A, Endres S, Rothenfusser S (2011). Pattern recognition of viral nucleic acids by RIG-I-like helicases. J Mol Med (Berl)..

[17] Pichlmair A, Schulz O, Tan CP, Naslund TI, Liljestrom P, Weber F (2006). RIG-I-mediated antiviral responses to single-stranded RNA bearing 5’-phosphates. Science..

[18] Barber MR, Aldridge JR, Webster RG, Magor KE (2010). Association of RIG-I with innate immunity of ducks to influenza. Proc Natl Acad Sci U S A..

[19] Nazmi A, Dutta K, Basu A (2011). RIG-I mediates innate immune response in mouse neurons following Japanese encephalitis virus infection. PLoS One..

[20] Cardona CJ, Xing Z, Sandrock CE, Davis CE (2009). Avian influenza in birds and mammals. Comp Immunol Microbiol Infect Dis..

[21] Haller O, Stertz S, Kochs G (2007). The Mx GTPase family of interferon-induced antiviral proteins. Microbes Infect..

[22] Ko JH, Jin HK, Asano A, Takada A, Ninomiya A, Kida H (2002). Polymorphisms and the differential antiviral activity of the chicken Mx gene. Genome Res..

[23] Han C, Wang J, Li L, Zhang Z, Wang L, Pan Z (2009). The role of insulin and glucose in goose primary hepatocyte triglyceride accumulation. J Exp Biol..

[24] Zhu LH, Meng H, Duan XJ, Xu GQ, Zhang J, Gong DQ (2011). Gene expression profile in the liver tissue of geese after overfeeding. Poult Sci..

[25] Mourot J, Guy G, Lagarrigue S, Peiniau P, Hermier D (2000). Role of hepatic lipogenesis in the susceptibility to fatty liver in the goose (Anser anser). Comp Biochem Physiol B Biochem Mol Biol..

[26] DiRusso CC, Li H, Darwis D, Watkins PA, Berger J, Black PN (2005). Comparative biochemical studies of the murine fatty acid transport proteins (FATP) expressed in yeast. J Biol Chem..

[27] Packard CJ, Demant T, Stewart JP, Bedford D, Caslake MJ, Schwertfeger G (2000). Apolipoprotein B metabolism and the distribution of VLDL and LDL subfractions. J Lipid Res..

[28] Fisher EA (2012). The degradation of apolipoprotein B100: multiple opportunities to regulate VLDL triglyceride production by different proteolytic pathways. Biochim Biophys Acta..

[29] Mauvoisin D, Mounier C (2011). Hormonal and nutritional regulation of SCD1 gene expression. Biochimie..

[30] Dobrzyn A, Ntambi JM (2005). The role of stearoyl-CoA desaturase in the control of metabolism. Prostaglandins Leukot Essent Fatty Acids..

[31] Mainieri D, Summermatter S, Seydoux J, Montani JP, Rusconi S, Russell AP (2006). A role for skeletal muscle stearoyl-CoA desaturase 1 in control of thermogenesis. FASEB J..

[32] Flowers MT, Ntambi JM (2009). Stearoyl-CoA desaturase and its relation to high-carbohydrate diets and obesity. Biochim Biophys Acta..

[33] Mauvoisin D, Prevost M, Ducheix S, Arnaud MP, Mounier C (2010). Key role of the ERK1/2 MAPK pathway in the transcriptional regulation of the Stearoyl-CoA Desaturase (SCD1) gene expression in response to leptin. Mol Cell Endocrinol..

[34] Ntambi JM, Miyazaki M, Stoehr JP, Lan H, Kendziorski CM, Yandell BS (2002). Loss of stearoyl-CoA desaturase-1 function protects mice against adiposity. Proc Natl Acad Sci U S A..

[35] Denver RJ, Bonett RM, Boorse GC (2011). Evolution of leptin structure and function. Neuroendocrinology..

[36] Pitel F, Faraut T, Bruneau G, Monget P (2010). Is there a leptin gene in the chicken genome? Lessons from phylogenetics, bioinformatics and genomics. Gen Comp Endocrinol..

[37] Prokop JW, Schmidt C, Gasper D, Duff RJ, Milsted A, Ohkubo T (2014). Discovery of the elusive leptin in birds: identification of several ‘missing links’ in the evolution of leptin and its receptor. PLoS One..

[38] Gentile CL, Pagliassotti MJ (2008). The role of fatty acids in the development and progression of nonalcoholic fatty liver disease. J Nutr Biochem..

[39] Ricchi M, Odoardi MR, Carulli L, Anzivino C, Ballestri S, Pinetti A (2009). Differential effect of oleic and palmitic acid on lipid accumulation and apoptosis in cultured hepatocytes. J Gastroenterol Hepatol..

[40] Diehl AM (2005). Lessons from animal models of NASH. Hepatol Res..

[41] Leclercq IA, Farrell GC, Schriemer R, Robertson GR (2002). Leptin is essential for the hepatic fibrogenic response to chronic liver injury. J Hepatol..

[42] Ikejima K, Honda H, Yoshikawa M, Hirose M, Kitamura T, Takei Y (2001). Leptin augments inflammatory and profibrogenic responses in the murine liver induced by hepatotoxic chemicals. Hepatology..

[43] Pilo B, George JC (1983). Diurnal and seasonal variation in liver glycogen and fat in relation to metabolic status of liver and m. pectoralis in the migratory starling, Sturnus roseus, wintering in India. Comp Biochem Physiol A Comp Physiol.

[44] Li R, Fan W, Tian G, Zhu H, He L, Cai J (2010). The sequence and de novo assembly of the giant panda genome. Nature..

[45] Price AL, Jones NC, Pevzner PA (2005). De novo identification of repeat families in large genomes. Bioinformatics..

[46] Tarailo-Graovac M, Chen N. Using RepeatMasker to identify repetitive elements in genomic sequences. Curr Protoc Bioinformatics. 2009;Chapter 4:Unit 4 10. 10.1002/0471250953.bi0410s25 19274634

[47] Jurka J, Kapitonov VV, Pavlicek A, Klonowski P, Kohany O, Walichiewicz J (2005). Repbase Update, a database of eukaryotic repetitive elements. Cytogenet Genome Res..

[48] Repeat Masker. http://www.repeatmasker.org.

[49] Repbase Update. http://www.girinst.org/repbase.

[50] Birney E, Clamp M, Durbin R (2004). GeneWise and Genomewise. Genome Res..

[51] Stanke M, Keller O, Gunduz I, Hayes A, Waack S, Morgenstern B (2006). AUGUSTUS: ab initio prediction of alternative transcripts. Nucleic Acids Res..

[52] Salamov AA, Solovyev VV (2000). Ab initio gene finding in Drosophila genomic DNA. Genome Res..

[53] Elsik CG, Mackey AJ, Reese JT, Milshina NV, Roos DS, Weinstock GM (2007). Creating a honey bee consensus gene set. Genome Biol..

[54] Trapnell C, Pachter L, Salzberg SL (2009). TopHat: discovering splice junctions with RNA-Seq. Bioinformatics..

[55] Trapnell C, Williams BA, Pertea G, Mortazavi A, Kwan G, van Baren MJ (2010). Transcript assembly and quantification by RNA-Seq reveals unannotated transcripts and isoform switching during cell differentiation. Nat Biotechnol..

[56] Bairoch A, Apweiler R (2000). The SWISS-PROT protein sequence database and its supplement TrEMBL in 2000. Nucleic Acids Res..

[57] Quevillon E, Silventoinen V, Pillai S, Harte N, Mulder N, Apweiler R (2005). InterProScan: protein domains identifier. Nucleic Acids Res..

[58] Kanehisa M, Goto S (2000). KEGG: kyoto encyclopedia of genes and genomes. Nucleic Acids Res..

[59] Lowe TM, Eddy SR (1997). tRNAscan-SE: a program for improved detection of transfer RNA genes in genomic sequence. Nucleic Acids Res..

[60] Nawrocki EP, Kolbe DL, Eddy SR (2009). Infernal 1.0: inference of RNA alignments. Bioinformatics..

[61] Griffiths-Jones S, Moxon S, Marshall M, Khanna A, Eddy SR, Bateman A (2005). Rfam: annotating non-coding RNAs in complete genomes. Nucleic Acids Res..

[62] Li H, Coghlan A, Ruan J, Coin LJ, Heriche JK, Osmotherly L (2006). TreeFam: a curated database of phylogenetic trees of animal gene families. Nucleic Acids Res..

[63] Edgar RC (2004). MUSCLE: multiple sequence alignment with high accuracy and high throughput. Nucleic Acids Res..

[64] Yang Z (2007). PAML 4: phylogenetic analysis by maximum likelihood. Mol Biol Evol..

[65] Huelsenbeck JP, Ronquist F (2001). MRBAYES: Bayesian inference of phylogenetic trees. Bioinformatics..

[66] Allen E, Xie Z, Gustafson AM, Carrington JC (2005). microRNA-directed phasing during trans-acting siRNA biogenesis in plants. Cell.

[67] Schwab R, Palatnik JF, Riester M, Schommer C, Schmid M, Weigel D (2005). Specific effects of microRNAs on the plant transcriptome. Dev Cell..

