# Supplementary material for: The goose genome sequence leads to insights into the evolution of waterfowl and susceptibility to fatty liver
Source: Genome Biol. 2015 May 6;16(1):89. doi: 10.1186/s13059-015-0652-y (PMC4419397; doi:10.1186/s13059-015-0652-y)
Supplement: Additional file 1: Table S1. — Statistics for raw data and clean data. Table S2. EST evaluation of the goose genome assembly. Table S3. General statistics for predicting protein-coding genes. Table S4. Summary of evidence from the GLEAN gene model. Table S5. Functional annotation statistics. Table S6. General statistics regarding repetitive genome sequences. Table S7. Non-coding RNA genes in the genome. Table S8. Aligned sequence lengths of four birds. Table S9. Intra-chromosomal and inter-chromosomal rearrangements in the goose and chicken genomes. Table S10. Detailed information regarding intra-chromosomal and inter-chromosomal rearrangement in goose and chicken chromosomes. Table S11. GO-enrichment profiles in the expanded goose gene family. Table S12. Rapidly and slowly evolved GO terms. Table S13. Positive-selection genes found in the goose and duck genomes. Table S14. MHC region differences between the goose, duck, and chicken genomes. Table S15. Gene alignment information for the goose and chicken genome in the MHC region. Table S16. Copy-number variations of innate immunity genes. Table S17. RIG-I gene alignment results for six species. Table S18. Alignment information for the goose, duck, and zebra-finch RIG-I genes. Table S19. Alignment information for RIG-I gene fragments of chicken and turkey. Table S20. Carcass traits of geese after overfeeding. Table S21. Plasma parameters of geese after overfeeding. Table S22. Copy-number variation of glucolipid metabolism-related genes in geese and other animals. Table S23. Comparison of the chicken, duck, goose, human, and mouse lep gene sequences. Table S24. miRNA information corresponding to glucolipid metabolism-related genes. [file 13059_2015_652_MOESM1_ESM.doc]

**Additional file 1**

**Table S1. Statistics for raw data and clean data.**

| **Insert size** | **Raw data** | | | | **Clean data** | | | |
| --- | --- | --- | --- | --- | --- | --- | --- | --- |
|  | **Total data (G)** | **Read length** | **Sequence coverage (X)** | **Physical coverage (X)** | **Total data (G)** | **Read length** | **Sequence coverage (X)** | **Physical coverage (X)** |
| 200 bp | 32.52 | 100 | 25.01 | 21.26 | 27.25 | 90.66 | 20.96 | 17.81 |
| 500 bp | 33.45 | 100 | 25.73 | 64.33 | 26.87 | 92.58 | 20.67 | 51.68 |
| 800 bp | 25.1 | 100 | 19.31 | 77.23 | 18.89 | 89.55 | 14.53 | 58.13 |
| 2 kb | 18.77 | 49 | 14.44 | 294.64 | 15.31 | 49 | 11.77 | 240.29 |
| 5 kb | 15.86 | 49 | 12.2 | 622.62 | 12.61 | 49 | 9.7 | 494.8 |
| 10 kb | 5.9 | 49 | 4.54 | 463.42 | 2.62 | 49 | 2.02 | 206 |
| 20 kb | 7.94 | 49 | 6.11 | 1246.94 | 3.69 | 49 | 2.84 | 579.1 |
| Total | 139.55 |  | 107.35 | 2790.44 | 107.23 |  | 82.49 | 1647.79 |

**Table S2. EST evaluation of the goose genome assembly.**

| **Dataset** | **Number** | **Total length (bp)** | **Bases covered by assembly (%)** | **Sequences covered by assembly (%)** | **With >90% sequence in one scaffold** | | **With >50% sequence in one scaffold** | |
| --- | --- | --- | --- | --- | --- | --- | --- | --- |
|  |  |  |  |  | **Number** | **Percent** | **Number** | **Percent** |
| >0 bp | 153,987 | 78,639,561 | 98.36 | 99.92 | 134,851 | 87.57 | 152,969 | 99.34 |
| >200 bp | 153,987 | 78,639,561 | 98.36 | 99.92 | 134,851 | 87.57 | 152,969 | 99.34 |
| >500 bp | 43,981 | 46,011,505 | 98.06 | 99.98 | 39,571 | 89.97 | 43,605 | 99.15 |
| >1,000 bp | 15,487 | 26,454,368 | 98.22 | 100 | 14,186 | 91.60 | 15,392 | 99.39 |

**Table S3. General statistics for predicting protein-coding genes.**

| **Gene set** | | **Number** | **Average gene length (bp)** | **Average CDS length (bp)** | **Average exon per gene** | **Average exon length (bp)** | **Average intron length (bp)** |
| --- | --- | --- | --- | --- | --- | --- | --- |
| *De novo* | *AUGUSTUS* | 17,555 | 38,805.35 | 1,532.52 | 9.6 | 159.58 | 4,332.41 |
|  | *GENSCAN* | 39,084 | 21,050.58 | 1,390.05 | 8.08 | 172.04 | 2,776.91 |
| Homolog | *G. gallus* | 43,719 | 8,934.78 | 748.95 | 4.29 | 174.53 | 2,487.19 |
|  | *H. sapiens* | 14,877 | 19,993.43 | 1,482.55 | 8.77 | 169.1 | 2,383.14 |
|  | *T. guttata* | 16,938 | 17,393.38 | 1,287.21 | 7.8 | 165.1 | 2,369.71 |
|  | *M. gallopavo* | 31,261 | 10,902.56 | 887.85 | 5.07 | 175.17 | 2,461.49 |
| *GLEAN* | | 13,530 | 24,670.85 | 1,697.70 | 9.73 | 174.47 | 2,631.29 |
| RNA-Seq | | 16,033 | 22,436.45 | 1,518.06 | 8.61 | 176.4 | 2,648.18 |
| Final set | | 16,150 | 23,245.04 | 2,270.93 | 9.39 | 241.74 | 2,498.7 |

The Homolog (*G. gallus*, *H. sapiens*, *T. guttata*, and *M. gallopavo*) and *De novo* (GENSCAN, AUGUSTUS) approaches were used for predicting protein-coding genes. Results were consolidated using the *GLEAN* program.

**Table S4. Summary of evidence from the GLEAN gene model.**

|  | **>=20% overlap** | | **>=50% overlap** | | **>=80% overlap** | |
| --- | --- | --- | --- | --- | --- | --- |
|  | **No.** | **Ratio (%)** | **No.** | **Ratio (%)** | **No.** | **Ratio (%)** |
| P (single) | 359 | 2.22 | 568 | 3.52 | 1,895 | 11.73 |
| P (more) | 1,704 | 10.55 | 2,258 | 13.98 | 2,258 | 13.98 |
| H (single) | 98 | 0.61 | 105 | 0.65 | 147 | 0.91 |
| H (more) | 317 | 1.96 | 950 | 5.88 | 950 | 5.88 |
| P+H | 11,815 | 73.16 | 11,106 | 68.77 | 8,473 | 52.46 |

**Table S5. Functional annotation statistics.**

|  | **Number** | **Percent (%)** |
| --- | --- | --- |
| Total | 16,150 |  |
| InterPro | 10,767 | 66.67 |
| GO | 9,159 | 56.717 |
| KEGG | 9,730 | 60.25 |
| Swissprot | 12,198 | 75.53 |
| TrEMBL | 12,512 | 77.47 |
| Annotated | 12,553 | 77.73 |
| Unannotated | 3,597 | 22.27 |

Five protein databases were used for predicting gene functions, including the InterPro, Gene ontology, KEGG, Swiss-Prot, and TrEMBL databases. The table shows the numbers of gene matches for each database.

**Table S6. General statistics regarding repetitive genome sequences.**

| **Type** | **Goose** | | **Duck** | | **Chicken** | | **Turkey** | | **Zebra finch** | |
| --- | --- | --- | --- | --- | --- | --- | --- | --- | --- | --- |
|  | **Repeat size** | **% of genome** | **Repeat size** | **% of genome** | **Repeat size** | **% of genome** | **Repeat size** | **% of genome** | **Repeat size** | **% of genome** |
| Trf | 14,128,568 | 1.26 | 15,782,811 | 1.43 | 12,286,555 | 1.11 | 4,998,615 | 0.47 | 23,411,148 | 1.92 |
| Repeatmasker | 58,555,338 | 5.22 | 58,699,602 | 5.31 | 93,902,804 | 8.47 | 79,516,130 | 7.49 | 67,803,101 | 5.55 |
| Total | 72,683,906 | 6.33 | 74,482,413 | 6.74 | 104,952,076 | 9.47 | 83,745,511 | 7.89 | 89,553,322 | 7.33 |

**Table S7. Non-coding RNA genes in the genome**

| **Type** | | **Copy number** | **Average length (bp)** | **Total length (bp)** | **% of genome** |
| --- | --- | --- | --- | --- | --- |
| miRNA | | 153 | 85.15 | 13,028 | 0.001 |
| tRNA | | 226 | 75.00 | 16,949 | 0.002 |
|  | rRNA | 69 | 108.67 | 7,498 | 0.001 |
| rRNA | 18S | 10 | 144.90 | 1,449 | 0.000 |
|  | 28S | 47 | 110.70 | 5,203 | 0.000 |
|  | 5.8S | 2 | 122.50 | 245 | 0.000 |
|  | 5S | 10 | 60.10 | 601 | 0.000 |
|  | snRNA | 206 | 118.44 | 24,399 | 0.002 |
| snRNA | CD-box | 103 | 92.48 | 9,525 | 0.001 |
|  | HACA-box | 48 | 141.90 | 6,811 | 0.001 |
|  | Splicing | 46 | 142.65 | 6,562 | 0.001 |

**Table S8. Aligned sequence lengths of *A. cygnoides* vs. *G. gallus, A. platyrhynchos*, and *T. guttata*.**

| **Species vs. species** | **Aligned length (bp)** | **Target genome coverage rate (%)** | **Query genome coverage rate (%)** |
| --- | --- | --- | --- |
| Goose vs. chicken | 759,342,668 | 67.67 | 68.50 |
| Goose vs. duck | 909,970,149 | 81.09 | 82.35 |
| Goose vs. zebra finch | 636,889,291 | 56.75 | 52.13 |

**Table S9. Intra-chromosomal and inter-chromosomal rearrangements in the goose and chicken genomes.** Syntenic segments are defined as continuous regions without order or orientation changes. Different cutoffs of syntenic segments, in the range of 5 kb to 200 kb, were used to find inter-chromosomal and intra-chromosomal breakpoints. The chicken genome was used as an outgroup to distinguish rearrangements in the goose and duck genomes.

| Cutoff (kb) | Intra-chromosomal rearrangement | | | | Inter-chromosomal rearrangement | | | |
| --- | --- | --- | --- | --- | --- | --- | --- | --- |
|  | Number of scaffolds | Number of breakpoints | Number of geese changed | Number of ducks changed | Number of scaffolds | Number of breakpoints | Number of geese changed | Number of ducks changed |
| 5 | 67 | 234 | 29 | 171 | 314 | 1,323 | 68 | 1,178 |
| 10 | 44 | 165 | 16 | 122 | 311 | 1,307 | 57 | 1,167 |
| 20 | 33 | 125 | 12 | 92 | 305 | 1,288 | 52 | 1,151 |
| 50 | 11 | 51 | 8 | 30 | 298 | 1,246 | 46 | 1,114 |
| 100 | 6 | 38 | 8 | 24 | 282 | 1,176 | 41 | 1,061 |
| 200 | 3 | 21 | 6 | 15 | 238 | 855 | 32 | 771 |

**Table S10. Detailed information regarding intra-chromosomal and inter-chromosomal rearrangement in goose and chicken chromosomes.**

| **Scaffold ID** | **Pos start** | **Pos end** | **Pos start** | **Pos end** |  |  |
| --- | --- | --- | --- | --- | --- | --- |
| scaffold125 | 1339768 | 1458453 | 1458453 | 1549452 | 50000 | duck_changed |
| scaffold125 | 1458453 | 1549452 | 1549452 | 1782961 | 50000 | duck_changed |
| scaffold2 | 4360663 | 4463147 | 4463147 | 4523573 | 50000 |  |
| scaffold13 | 625268 | 1118425 | 1118425 | 1206620 | 50000 |  |
| scaffold13 | 1118425 | 1206620 | 1206620 | 1332398 | 50000 |  |
| scaffold86 | 8361 | 1151659 | 1151682 | 1808934 | 500000 | duck_changed |
| scaffold86 | 1151682 | 1808934 | 1809058 | 1912729 | 100000 | duck_changed |
| scaffold86 | 1809058 | 1912729 | 1912751 | 2056468 | 100000 | duck_changed |
| scaffold86 | 1912751 | 2056468 | 2056469 | 2267038 | 100000 | duck_changed |
| scaffold86 | 2056469 | 2267038 | 2267198 | 3043155 | 200000 | duck_changed |
| scaffold86 | 2267198 | 3043155 | 3044648 | 4185192 | 500000 | duck_changed |
| scaffold86 | 3044648 | 4185192 | 4185215 | 5780277 | 500000 | duck_changed |
| scaffold86 | 4185215 | 5780277 | 5780782 | 8201792 | 500000 | duck_changed |
| scaffold111 | 3254 | 386540 | 389529 | 450245 | 50000 | duck_changed |
| scaffold111 | 389529 | 450245 | 579552 | 689940 | 50000 | duck_changed |
| scaffold111 | 579552 | 689940 | 885306 | 1121586 | 100000 | duck_changed |
| scaffold137 | 0 | 532209 | 532513 | 744488 | 200000 |  |
| scaffold137 | 532513 | 744488 | 867045 | 1008858 | 100000 |  |
| scaffold137 | 867045 | 1008858 | 1008864 | 1444728 | 100000 |  |
| scaffold137 | 1008864 | 1444728 | 1475658 | 2089583 | 200000 |  |
| scaffold137 | 1475658 | 2089583 | 2095302 | 2564875 | 200000 |  |
| scaffold137 | 2095302 | 2564875 | 2565567 | 2941905 | 200000 |  |
| scaffold65 | 7060866 | 7406258 | 7436540 | 9725669 | 200000 | goose_changed |
| scaffold65 | 7436540 | 9725669 | 9725675 | 9834892 | 100000 | goose_changed |
| scaffold65 | 9725675 | 9834892 | 9835190 | 10161660 | 100000 | goose_changed |
| scaffold65 | 9835190 | 10161660 | 10162174 | 11017805 | 200000 | goose_changed |
| scaffold65 | 10162174 | 11017805 | 11074795 | 11411571 | 200000 | goose_changed |
| scaffold65 | 11074795 | 11411571 | 11411610 | 13101265 | 200000 | goose_changed |
| scaffold65 | 11411610 | 13101265 | 13101276 | 14743648 | 500000 | goose_changed |
| scaffold591 | 182405 | 292768 | 442232 | 746122 | 100000 | goose_changed |
| scaffold141 | 72416 | 398415 | 398438 | 952843 | 200000 | duck_changed |
| scaffold141 | 398438 | 952843 | 952866 | 1516431 | 500000 | duck_changed |
| scaffold141 | 952866 | 1516431 | 1517907 | 2428722 | 500000 | duck_changed |
| scaffold141 | 1517907 | 2428722 | 2429392 | 3760643 | 500000 | duck_changed |
| scaffold141 | 2429392 | 3760643 | 3760747 | 5163730 | 500000 | duck_changed |
| scaffold141 | 3760747 | 5163730 | 5163753 | 5422017 | 200000 | duck_changed |
| scaffold141 | 5163753 | 5422017 | 5422024 | 6199829 | 200000 | duck_changed |
| scaffold141 | 5422024 | 6199829 | 6199834 | 6475730 | 200000 | duck_changed |
| scaffold141 | 6199834 | 6475730 | 6494384 | 6817939 | 200000 | duck_changed |
| scaffold141 | 6494384 | 6817939 | 6818526 | 7172888 | 200000 | duck_changed |
| scaffold141 | 6818526 | 7172888 | 7262984 | 7473459 | 200000 | duck_changed |
| scaffold112 | 160 | 60795 | 61116 | 476008 | 50000 |  |
| scaffold112 | 61116 | 476008 | 477064 | 915153 | 200000 |  |
| scaffold112 | 477064 | 915153 | 915268 | 1046066 | 100000 |  |
| scaffold112 | 915268 | 1046066 | 1050160 | 1944168 | 100000 |  |
| scaffold73 | 18073 | 478359 | 479733 | 1298390 | 200000 | duck_changed |
| scaffold73 | 479733 | 1298390 | 1362622 | 1921998 | 500000 | duck_changed |
| scaffold73 | 1362622 | 1921998 | 1922472 | 2507409 | 500000 | duck_changed |
| scaffold73 | 1922472 | 2507409 | 2507601 | 2585154 | 50000 | duck_changed |
| scaffold73 | 2507601 | 2585154 | 2597722 | 3035002 | 50000 | duck_changed |
| scaffold73 | 2597722 | 3035002 | 3047360 | 3170956 | 100000 | duck_changed |

**Table S11. GO-enrichment profiles in the expanded goose gene family.**

| GO_ID | GO_Term | Gene_num | *P* value |
| --- | --- | --- | --- |
| GO:0006259 | DNA metabolic process | 45 | 1.90E-69 |
| GO:0003964 | RNA-directed DNA polymerase activity | 39 | 4.18E-62 |
| GO:0006278 | RNA-dependent DNA replication | 39 | 4.41E-61 |
| GO:0004984 | Olfactory receptor activity | 44 | 1.32E-50 |
| GO:0006260 | DNA replication | 41 | 1.23E-44 |
| GO:0004888 | Transmembrane signaling receptor activity | 52 | 2.29E-44 |
| GO:0007186 | G-protein coupled receptor signaling pathway | 53 | 3.75E-39 |
| GO:0003723 | RNA binding | 41 | 4.64E-28 |
| GO:0016772 | Transferase activity, transferring phosphorus-containing groups | 52 | 1.36E-22 |
| GO:0016021 | Integral to membrane | 59 | 7.83E-15 |
| GO:0034645 | Cellular macromolecule biosynthetic process | 46 | 8.06E-13 |
| GO:0090304 | Nucleic acid metabolic process | 48 | 1.14E-12 |
| GO:0007165 | Signal transduction | 56 | 7.91E-09 |
| GO:0003676 | Nucleic acid binding | 54 | 1.31E-08 |
| GO:0007154 | Cell communication | 57 | 3.48E-08 |
| GO:0005003 | Ephrin receptor activity | 6 | 1.16E-07 |
| GO:0006807 | Nitrogen compound metabolic process | 51 | 1.29E-07 |
| GO:0015629 | Actin cytoskeleton | 10 | 1.78E-06 |
| GO:0009987 | Cellular process | 130 | 1.89E-06 |
| GO:0016020 | Membrane | 74 | 6.90E-06 |
| GO:0005869 | Dynactin complex | 3 | 1.21E-05 |
| GO:0007040 | Lysosome organization | 3 | 1.21E-05 |
| GO:0004348 | Glucosylceramidase activity | 3 | 4.78E-05 |
| GO:0016459 | Myosin complex | 7 | 5.04E-05 |
| GO:0050794 | Regulation of cellular process | 63 | 8.04E-05 |
| GO:0005764 | Lysosome | 3 | 0.0001174 |
| GO:0005833 | Hemoglobin complex | 3 | 0.0002307 |
| GO:0005853 | Eukaryotic translation elongation factor 1 complex | 2 | 0.0005309 |
| GO:0004861 | Cyclin-dependent protein serine/threonine kinase inhibitor activity | 2 | 0.0005309 |
| GO:0006665 | Sphingolipid metabolic process | 3 | 0.0006243 |
| GO:1901363 | Heterocyclic compound binding | 76 | 0.0006366 |
| GO:0097159 | Organic cyclic compound binding | 76 | 0.0007298 |
| GO:0015671 | Oxygen transport | 3 | 0.0009205 |
| GO:0019825 | Oxygen binding | 3 | 0.0009205 |
| GO:0044430 | Cytoskeletal part | 10 | 0.0011149 |
| GO:0044260 | Cellular macromolecule metabolic process | 59 | 0.0012406 |
| GO:0003746 | Translation elongation factor activity | 2 | 0.0015685 |
| GO:0044763 | Single-organism cellular process | 73 | 0.0024668 |
| GO:0019068 | Virion assembly | 2 | 0.0030892 |
| GO:0044421 | Extracellular region part | 7 | 0.0038864 |
| GO:0003774 | Motor activity | 7 | 0.0059479 |
| GO:0006414 | Translational elongation | 2 | 0.0103277 |
| GO:0007214 | Gamma-aminobutyric acid signaling pathway | 2 | 0.0103277 |
| GO:0044699 | Single-organism process | 77 | 0.0166799 |

**Table S12. Rapidly and slowly evolved GO terms.**

| **Taxa ID** | **Goose-duck ka/ks** | **Chicken-turkey ka/ks** | **Ratio** | **Function description** | **Function class** |
| --- | --- | --- | --- | --- | --- |
| GO:0006413 | 0.07025 | 0.0092 | 7.635869565 | Translational initiation | Biological process |
| GO:0004879 | 0.098 | 0.0409 | 2.39608802 | Ligand-activated sequence-specific DNA binding RNA polymerase II transcription factor activity | Molecular function |
| GO:0004890 | 0.0561 | 0.0308 | 1.821428571 | GABA-A receptor activity | Molecular function |
| GO:0016779 | 0.0792 | 0.04525 | 1.750276243 | Nucleotidyltransferase activity | Molecular function |
| GO:0008378 | 0.1322 | 0.077 | 1.716883117 | Galactosyltransferase activity | Molecular function |
| GO:0000226 | 0.15925 | 0.098 | 1.625 | Microtubule cytoskeleton organization | Biological process |
| GO:0008565 | 0.05305 | 0.0359 | 1.477715877 | Protein transporter activity | Molecular function |
| GO:0006821 | 0.04815 | 0.0333 | 1.445945946 | Chloride transport | Biological process |
| GO:0008146 | 0.0838 | 0.0582 | 1.439862543 | Sulfotransferase activity | Molecular function |
| GO:0008138 | 0.13935 | 0.0982 | 1.41904277 | Protein tyrosine/serine/threonine phosphatase activity | Molecular function |
| GO:0016616 | 0.0994 | 0.0703 | 1.413940256 | Oxidoreductase activity, acting on the CH-OH group of donors, NAD or NADP as acceptor | Molecular function |
| GO:0003924 | 0.03505 | 0.0249 | 1.407630522 | GTPase activity | Molecular function |
| GO:0051082 | 0.07825 | 0.0565 | 1.384955752 | Unfolded protein binding | Molecular function |
| GO:0005923 | 0.1349 | 0.0977 | 1.380757421 | Tight junction | Cellular component |
| GO:0016592 | 0.0474 | 0.03435 | 1.379912664 | Mediator complex | Cellular component |
| GO:0016820 | 0.0666 | 0.0491 | 1.356415479 | Hydrolase activity, acting on acid anhydrides, catalyzing transmembrane movement of substances | Molecular function |
| GO:0003707 | 0.0568 | 0.0423 | 1.342789598 | Steroid hormone receptor activity | Molecular function |
| GO:0007155 | 0.1326 | 0.09965 | 1.330657301 | Cell adhesion | Biological process |
| GO:0008021 | 0.0861 | 0.06485 | 1.32767926 | Synaptic vesicle | Cellular component |
| GO:0015031 | 0.0503 | 0.0381 | 1.320209974 | Protein transport | Biological process |
| GO:0003755 | 0.1076 | 0.0816 | 1.318627451 | Peptidyl-prolyl cis-trans isomerase activity | Molecular function |
| GO:0005694 | 0.2155 | 0.1638 | 1.315628816 | Chromosome | Cellular component |
| GO:0006357 | 0.0467 | 0.0355 | 1.315492958 | Regulation of transcription from RNA polymerase II promoter | Biological process |
| GO:0006260 | 0.144 | 0.1095 | 1.315068493 | DNA replication | Biological process |
| GO:0005874 | 0.1143 | 0.0874 | 1.30778032 | Microtubule | Cellular component |
| GO:0007017 | 0.0151 | 0.0117 | 1.290598291 | Microtubule-based process | Biological process |
| GO:0005230 | 0.06215 | 0.0484 | 1.284090909 | Extracellular ligand-gated ion channel activity | Molecular function |
| GO:0005044 | 0.18385 | 0.14395 | 1.277179576 | Scavenger receptor activity | Molecular function |
| GO:0016757 | 0.111 | 0.0874 | 1.270022883 | Transferase activity, transferring glycosyl groups | Molecular function |
| GO:0004867 | 0.1819 | 0.14535 | 1.251461988 | Serine-type endopeptidase inhibitor activity | Molecular function |
| GO:0006816 | 0.0476 | 0.0384 | 1.239583333 | Calcium ion transport | Biological process |
| GO:0015662 | 0.0832 | 0.0675 | 1.232592593 | ATPase activity, coupled to transmembrane movement of ions, phosphorylative mechanism | Molecular function |
| GO:0003779 | 0.12525 | 0.10205 | 1.227339539 | Actin binding | Molecular function |
| GO:0032012 | 0.0601 | 0.0503 | 1.194831014 | Regulation of ARF protein signal transduction | Biological process |
| GO:0005086 | 0.0601 | 0.0503 | 1.194831014 | ARF guanyl-nucleotide exchange factor activity | Molecular function |
| GO:0015991 | 0.05225 | 0.0439 | 1.190205011 | ATP hydrolysis coupled proton transport | Biological process |
| GO:0006470 | 0.1101 | 0.0943 | 1.167550371 | Protein dephosphorylation | Biological process |
| GO:0030036 | 0.14185 | 0.12155 | 1.167009461 | Actin cytoskeleton organization | Biological process |
| GO:0000166 | 0.1066 | 0.0919 | 1.159956474 | Nucleotide binding | Molecular function |
| GO:0005856 | 0.08325 | 0.07235 | 1.150656531 | Cytoskeleton | Cellular component |
| GO:0006457 | 0.0853 | 0.07445 | 1.145735393 | Protein folding | Biological process |
| GO:0006511 | 0.0649 | 0.0567 | 1.144620811 | Ubiquitin-dependent protein catabolic process | Biological process |
| GO:0005634 | 0.0778 | 0.0681 | 1.142437592 | Nucleus | Cellular component |
| GO:0008233 | 0.12755 | 0.11225 | 1.136302895 | Peptidase activity | Molecular function |
| GO:0006812 | 0.0583 | 0.0514 | 1.134241245 | Cation transport | Biological process |
| GO:0003725 | 0.0787 | 0.0703 | 1.119487909 | Double-stranded RNA binding | Molecular function |
| GO:0004871 | 0.0836 | 0.0752 | 1.111702128 | Signal transducer activity | Molecular function |
| GO:0030001 | 0.13365 | 0.12085 | 1.105916425 | Metal ion transport | Biological process |
| GO:0006418 | 0.1243 | 0.1126 | 1.103907638 | tRNA aminoacylation for protein translation | Biological process |
| GO:0017111 | 0.09895 | 0.08985 | 1.101279911 | Nucleoside-triphosphatase activity | Molecular function |
| GO:0009058 | 0.08525 | 0.07805 | 1.092248559 | Biosynthetic process | Biological process |
| GO:0005328 | 0.1089 | 0.10035 | 1.085201794 | Neurotransmitter:sodium symporter activity | Molecular function |
| GO:0009451 | 0.13225 | 0.1224 | 1.080473856 | RNA modification | Biological process |
| GO:0003723 | 0.0991 | 0.0921 | 1.076004343 | RNA binding | Molecular function |
| GO:0004553 | 0.19835 | 0.18455 | 1.074776483 | Hydrolase activity, hydrolyzing O-glycosyl compounds | Molecular function |
| GO:0020037 | 0.12815 | 0.11945 | 1.072833822 | Heme binding | Molecular function |
| GO:0008289 | 0.1207 | 0.1126 | 1.071936057 | Lipid binding | Molecular function |
| GO:0007160 | 0.1192 | 0.11135 | 1.070498428 | Cell-matrix adhesion | Biological process |
| GO:0016881 | 0.06495 | 0.06085 | 1.0673788 | Acid-amino acid ligase activity | Molecular function |
| GO:0007205 | 0.081 | 0.0759 | 1.067193676 | Activation of protein kinase C activity by G-protein coupled receptor protein signaling pathway | Biological process |
| GO:0004143 | 0.081 | 0.0759 | 1.067193676 | Diacylglycerol kinase activity | Molecular function |
| GO:0008234 | 0.1859 | 0.17535 | 1.060165384 | Cysteine-type peptidase activity | Molecular function |
| GO:0006355 | 0.0729 | 0.0693 | 1.051948052 | Regulation of transcription, DNA-dependent | Biological process |
| GO:0008484 | 0.1434 | 0.1365 | 1.050549451 | Sulfuric ester hydrolase activity | Molecular function |
| GO:0005102 | 0.1265 | 0.1206 | 1.048922056 | Receptor binding | Molecular function |
| GO:0005179 | 0.18315 | 0.17565 | 1.042698548 | Hormone activity | Molecular function |
| GO:0051258 | 0.1139 | 0.1094 | 1.041133455 | Protein polymerization | Biological process |
| GO:0005975 | 0.1333 | 0.1281 | 1.040593286 | Carbohydrate metabolic process | Biological process |
| GO:0005576 | 0.14425 | 0.13875 | 1.03963964 | Extracellular region | Cellular component |
| GO:0003677 | 0.08415 | 0.081 | 1.038888889 | DNA binding | Molecular function |
| GO:0008237 | 0.12055 | 0.1174 | 1.026831346 | Metallopeptidase activity | Molecular function |
| GO:0003700 | 0.071 | 0.0693 | 1.024531025 | Sequence-specific DNA binding transcription factor activity | Molecular function |
| GO:0003676 | 0.0877 | 0.0857 | 1.023337223 | Nucleic acid binding | Molecular function |
| GO:0004435 | 0.05885 | 0.0578 | 1.01816609 | Phosphatidylinositol phospholipase C activity | Molecular function |
| GO:0004725 | 0.0944 | 0.09325 | 1.01233244 | Protein tyrosine phosphatase activity | Molecular function |
| GO:0016791 | 0.1015 | 0.1006 | 1.008946322 | Phosphatase activity | Molecular function |
| GO:0043565 | 0.07225 | 0.0722 | 1.000692521 | Sequence-specific DNA binding | Molecular function |
| GO:0004629 | 0.0563 | 0.0563 | 1 | Phospholipase C activity | Molecular function |
| GO:0016311 | 0.1015 | 0.1021 | 0.994123408 | Dephosphorylation | Biological process |
| GO:0007166 | 0.0642 | 0.0646 | 0.99380805 | Cell surface receptor signaling pathway | Biological process |
| GO:0006886 | 0.05435 | 0.05515 | 0.985494107 | Intracellular protein transport | Biological process |
| GO:0008152 | 0.1118 | 0.1142 | 0.978984238 | Metabolic process | Biological process |
| GO:0008270 | 0.08915 | 0.0911 | 0.978594951 | Zinc ion binding | Molecular function |
| GO:0004386 | 0.0803 | 0.08265 | 0.971566848 | Helicase activity | Molecular function |
| GO:0016021 | 0.1058 | 0.11 | 0.961818182 | Integral to membrane | Cellular component |
| GO:0045211 | 0.0537 | 0.0561 | 0.957219251 | Postsynaptic membrane | Cellular component |
| GO:0007018 | 0.1047 | 0.1094 | 0.957038391 | Microtubule-based movement | Biological process |
| GO:0007165 | 0.0887 | 0.0928 | 0.955818966 | Signal transduction | Biological process |
| GO:0006836 | 0.0934 | 0.0978 | 0.955010225 | Neurotransmitter transport | Biological process |
| GO:0005524 | 0.08 | 0.0839 | 0.953516091 | ATP binding | Molecular function |
| GO:0005085 | 0.0615 | 0.0648 | 0.949074074 | Guanyl-nucleotide exchange factor activity | Molecular function |
| GO:0004674 | 0.048 | 0.05095 | 0.942100098 | Protein serine/threonine kinase activity | Molecular function |
| GO:0005515 | 0.0876 | 0.09305 | 0.941429339 | Protein binding | Molecular function |
| GO:0004222 | 0.1004 | 0.10685 | 0.939635002 | Metalloendopeptidase activity | Molecular function |
| GO:0004672 | 0.0728 | 0.0778 | 0.935732648 | Protein kinase activity | Molecular function |
| GO:0016459 | 0.06065 | 0.06485 | 0.935235158 | Myosin complex | Cellular component |
| GO:0003774 | 0.06065 | 0.06485 | 0.935235158 | Motor activity | Molecular function |
| GO:0006468 | 0.0718 | 0.0768 | 0.934895833 | Protein phosphorylation | Biological process |
| GO:0006396 | 0.1056 | 0.1132 | 0.932862191 | RNA processing | Biological process |
| GO:0003824 | 0.10865 | 0.11735 | 0.925862804 | Catalytic activity | Molecular function |
| GO:0016491 | 0.1286 | 0.139 | 0.925179856 | Oxidoreductase activity | Molecular function |
| GO:0005737 | 0.0965 | 0.1049 | 0.919923737 | Cytoplasm | Cellular component |
| GO:0006486 | 0.0784 | 0.0853 | 0.919109027 | Protein glycosylation | Biological process |
| GO:0005622 | 0.084 | 0.0916 | 0.917030568 | Intracellular | Cellular component |
| GO:0005506 | 0.1294 | 0.1414 | 0.915134371 | Iron ion binding | Molecular function |
| GO:0007264 | 0.0466 | 0.0512 | 0.91015625 | Small GTPase mediated signal transduction | Biological process |
| GO:0003777 | 0.1047 | 0.1155 | 0.906493506 | Microtubule motor activity | Molecular function |
| GO:0004888 | 0.0826 | 0.0912 | 0.905701754 | Transmembrane signaling receptor activity | Molecular function |
| GO:0055085 | 0.0967 | 0.1071 | 0.902894491 | Transmembrane transport | Biological process |
| GO:0046872 | 0.0796 | 0.0882 | 0.902494331 | Metal ion binding | Molecular function |
| GO:0006810 | 0.0742 | 0.08235 | 0.90103218 | Transport | Biological process |
| GO:0005509 | 0.0876 | 0.0973 | 0.900308325 | Calcium ion binding | Molecular function |
| GO:0006811 | 0.05705 | 0.0637 | 0.895604396 | Ion transport | Biological process |
| GO:0016887 | 0.10575 | 0.1182 | 0.894670051 | ATPase activity | Molecular function |
| GO:0007218 | 0.1262 | 0.1415 | 0.891872792 | Neuropeptide signaling pathway | Biological process |
| GO:0016020 | 0.075 | 0.08455 | 0.887049083 | Membrane | Cellular component |
| GO:0005097 | 0.0689 | 0.07795 | 0.883899936 | Rab GTPase activator activity | Molecular function |
| GO:0032313 | 0.0689 | 0.07795 | 0.883899936 | Regulation of Rab GTPase activity | Biological process |
| GO:0008026 | 0.0751 | 0.08545 | 0.878876536 | ATP-dependent helicase activity | Molecular function |
| GO:0008083 | 0.0745 | 0.0848 | 0.878537736 | Growth factor activity | Molecular function |
| GO:0044237 | 0.07725 | 0.0885 | 0.872881356 | Cellular metabolic process | Biological process |
| GO:0000287 | 0.1136 | 0.1302 | 0.87250384 | Magnesium ion binding | Molecular function |
| GO:0005840 | 0.07545 | 0.08685 | 0.868739206 | Ribosome | Cellular component |
| GO:0016788 | 0.12825 | 0.14855 | 0.863345675 | Hydrolase activity, acting on ester bonds | Molecular function |
| GO:0005578 | 0.07205 | 0.0836 | 0.861842105 | Proteinaceous extracellular matrix | Cellular component |
| GO:0004812 | 0.1096 | 0.1275 | 0.859607843 | Aminoacyl-tRNA ligase activity | Molecular function |
| GO:0005198 | 0.0767 | 0.0896 | 0.856026786 | Structural molecule activity | Molecular function |
| GO:0006629 | 0.09115 | 0.1066 | 0.855065666 | Lipid metabolic process | Biological process |
| GO:0030131 | 0.05925 | 0.06945 | 0.853131749 | Clathrin adaptor complex | Cellular component |
| GO:0050660 | 0.12775 | 0.15005 | 0.851382872 | Flavin adenine dinucleotide binding | Molecular function |
| GO:0003735 | 0.081 | 0.0953 | 0.849947534 | Structural constituent of ribosome | Molecular function |
| GO:0035023 | 0.08795 | 0.1036 | 0.848938224 | Regulation of Rho protein signal transduction | Biological process |
| GO:0005089 | 0.08795 | 0.1036 | 0.848938224 | Rho guanyl-nucleotide exchange factor activity | Molecular function |
| GO:0008324 | 0.06 | 0.0708 | 0.847457627 | Cation transmembrane transporter activity | Molecular function |
| GO:0006508 | 0.1149 | 0.13565 | 0.847032805 | Proteolysis | Biological process |
| GO:0005525 | 0.044 | 0.052 | 0.846153846 | GTP binding | Molecular function |
| GO:0006412 | 0.0812 | 0.0962 | 0.844074844 | Translation | Biological process |
| GO:0035556 | 0.06035 | 0.0721 | 0.8370319 | Intracellular signal transduction | Biological process |
| GO:0005488 | 0.07295 | 0.088 | 0.828977273 | Binding | Molecular function |
| GO:0016787 | 0.1176 | 0.1419 | 0.828752643 | Hydrolase activity | Molecular function |
| GO:0004221 | 0.0649 | 0.0784 | 0.827806122 | Ubiquitin thiolesterase activity | Molecular function |
| GO:0008080 | 0.0444 | 0.0537 | 0.826815642 | N-acetyltransferase activity | Molecular function |
| GO:0006464 | 0.06205 | 0.07545 | 0.82239894 | Protein modification process | Biological process |
| GO:0031012 | 0.06845 | 0.08345 | 0.820251648 | Extracellular matrix | Cellular component |
| GO:0031072 | 0.0645 | 0.0787 | 0.81956798 | Heat shock protein binding | Molecular function |
| GO:0006955 | 0.2276 | 0.2788 | 0.816355811 | Immune response | Biological process |
| GO:0009055 | 0.11355 | 0.14115 | 0.804463337 | Electron carrier activity | Molecular function |
| GO:0003887 | 0.13725 | 0.1708 | 0.803571429 | DNA-directed DNA polymerase activity | Molecular function |
| GO:0030170 | 0.1034 | 0.1291 | 0.800929512 | Pyridoxal phosphate binding | Molecular function |
| GO:0004872 | 0.07825 | 0.09795 | 0.798876978 | Receptor activity | Molecular function |
| GO:0032312 | 0.0572 | 0.0722 | 0.792243767 | Regulation of ARF GTPase activity | Biological process |
| GO:0008060 | 0.0572 | 0.0722 | 0.792243767 | ARF GTPase activator activity | Molecular function |
| GO:0055114 | 0.1098 | 0.139 | 0.789928058 | Oxidation-reduction process | Biological process |
| GO:0051056 | 0.0615 | 0.0785 | 0.78343949 | Regulation of small GTPase mediated signal transduction | Biological process |
| GO:0004930 | 0.06015 | 0.0774 | 0.777131783 | G-protein coupled receptor activity | Molecular function |
| GO:0004252 | 0.14525 | 0.1883 | 0.771375465 | Serine-type endopeptidase activity | Molecular function |
| GO:0006139 | 0.14885 | 0.1934 | 0.769648397 | Nucleobase-containing compound metabolic process | Biological process |
| GO:0007186 | 0.0786 | 0.103 | 0.763106796 | G-protein coupled receptor signaling pathway | Biological process |
| GO:0008168 | 0.13605 | 0.18175 | 0.748555708 | Methyltransferase activity | Molecular function |
| GO:0046983 | 0.1083 | 0.1466 | 0.738744884 | Protein dimerization activity | Molecular function |
| GO:0005216 | 0.0469 | 0.0637 | 0.736263736 | Ion channel activity | Molecular function |
| GO:0004181 | 0.1127 | 0.1573 | 0.716465353 | Metallocarboxypeptidase activity | Molecular function |
| GO:0005887 | 0.0699 | 0.0978 | 0.714723926 | Integral to plasma membrane | Cellular component |
| GO:0005215 | 0.0769 | 0.1081 | 0.711378353 | Transporter activity | Molecular function |
| GO:0007275 | 0.04625 | 0.06575 | 0.703422053 | Multicellular organismal development | Biological process |
| GO:0016758 | 0.0908 | 0.1304 | 0.696319018 | Transferase activity, transferring hexosyl groups | Molecular function |
| GO:0006281 | 0.14515 | 0.2101 | 0.690861495 | DNA repair | Biological process |
| GO:0005783 | 0.05665 | 0.08205 | 0.690432663 | Endoplasmic reticulum | Cellular component |
| GO:0003899 | 0.0982 | 0.1427 | 0.688156973 | DNA-directed RNA polymerase activity | Molecular function |
| GO:0007156 | 0.05905 | 0.08905 | 0.663110612 | Homophilic cell adhesion | Biological process |
| GO:0042981 | 0.116 | 0.178 | 0.651685393 | Regulation of apoptotic process | Biological process |
| GO:0030117 | 0.02275 | 0.0355 | 0.64084507 | Membrane coat | Cellular component |
| GO:0042626 | 0.11375 | 0.18065 | 0.629670634 | ATPase activity, coupled to transmembrane movement of substances | Molecular function |
| GO:0006814 | 0.06965 | 0.11095 | 0.627760252 | Sodium ion transport | Biological process |
| GO:0030173 | 0.0642 | 0.1026 | 0.625730994 | Integral to Golgi membrane | Cellular component |
| GO:0008373 | 0.0642 | 0.1026 | 0.625730994 | Sialyltransferase activity | Molecular function |
| GO:0006813 | 0.0382 | 0.06155 | 0.620633631 | Potassium ion transport | Biological process |
| GO:0045454 | 0.10935 | 0.17785 | 0.61484397 | Cell redox homeostasis | Biological process |
| GO:0005249 | 0.0382 | 0.0651 | 0.586789555 | Voltage-gated potassium channel activity | Molecular function |
| GO:0005096 | 0.0472 | 0.0807 | 0.58488228 | GTPase activator activity | Molecular function |
| GO:0008236 | 0.11635 | 0.19955 | 0.583061889 | Serine-type peptidase activity | Molecular function |
| GO:0008081 | 0.0537 | 0.0928 | 0.578663793 | Phosphoric diester hydrolase activity | Molecular function |
| GO:0007154 | 0.05265 | 0.09105 | 0.578253707 | Cell communication | Biological process |
| GO:0015035 | 0.10275 | 0.17785 | 0.577734046 | Protein disulfide oxidoreductase activity | Molecular function |
| GO:0016192 | 0.04855 | 0.0842 | 0.576603325 | Vesicle-mediated transport | Biological process |
| GO:0008076 | 0.0357 | 0.0649 | 0.550077042 | Voltage-gated potassium channel complex | Cellular component |
| GO:0035091 | 0.0534 | 0.0988 | 0.54048583 | Phosphatidylinositol binding | Molecular function |
| GO:0016773 | 0.0636 | 0.11815 | 0.538298773 | Phosphotransferase activity, alcohol group as acceptor | Molecular function |
| GO:0016849 | 0.0625 | 0.1365 | 0.457875458 | Phosphorus-oxygen lyase activity | Molecular function |
| GO:0009190 | 0.0625 | 0.1365 | 0.457875458 | Cyclic nucleotide biosynthetic process | Biological process |
| GO:0004114 | 0.0445 | 0.1631 | 0.272838749 | 3',5'-cyclic-nucleotide phosphodiesterase activity | Molecular function |

**Table S13. Positive-selection genes found in the goose and duck genomes.**

| **Goose gene** | **Duck gene** | ***P* value** | **Symbol** | **Description** |
| --- | --- | --- | --- | --- |
| ACY_000827 | Apl2_04118 | 2.89E-15 | FGFR2 | Fibroblast growth factor receptor 2 |
| ACY_001490 | Apl2_11013 | 9.23E-05 | RP-L36e, RPL36 | Large subunit ribosomal protein L36e |
| ACY_002440 | Apl2_04068 | 2.82E-10 | FMN1 | Formin 1 |
| ACY_003039 | Apl2_07506 | 8.82E-05 | DSG4 | Desmoglein 4 |
| ACY_003164 | Apl2_13320 | 4.06E-05 | IPR009311 | Interferon-induced 6-16 |
| ACY_003410 | Apl2_11812 | 9.04E-12 | CUL5 | Cullin 5 |
| ACY_003640 | Apl2_16648 | 0.00011221 | RNF24 | RING finger protein 24 |
| ACY_004808 | Apl2_14586 | 1.10E-05 | GATA1_2_3 | GATA-binding protein 1/2/3 |
| ACY_004989 | Apl2_01958 | 9.04E-06 | K07579 | Putative methylase |
| ACY_005018 | Apl2_11388 | 7.52E-11 | PIK3R | Phosphoinositide-3-kinase, regulatory subunit |
| ACY_005777 | Apl2_09651 | 0 | MYO18 | Myosin XVIII |
| ACY_006616 | Apl2_06342 | 2.80E-05 | HBZ | Hemoglobin subunit zeta |
| ACY_007158 | Apl2_04093 | 2.39E-08 | eIF-3A, EIF3S10 | Translation initiation factor eIF-3 subunit 10 |
| ACY_009883 | Apl2_05947 | 4.57E-10 | HNRNPABD | Heterogeneous nuclear ribonucleoprotein A/B/D |
| ACY_010069 | Apl2_06223 | 3.69E-05 | PLEKHG1 | Pleckstrin homology domain-containing family G member 1 |
| ACY_010154 | Apl2_08410 | 7.06E-08 | CX40.1 | Connexin 40.1 |
| ACY_010232 | Apl2_00040 | 0.000101457 | eIF-31, EIF3S1 | Translation initiation factor eIF-3 subunit 1 |
| ACY_012289 | Apl2_19571 | 1.61E-07 | IFIT1 | Interferon-induced protein with tetratricopeptide repeats 1 |
| ACY_015034 | Apl2_18162 | 1.27E-07 | HF1 | Complement factor H |
| ACY_015528 | Apl2_11196 | 2.39E-06 | KAI1, CD82 | Kangai 1 |
| ACY_015663 | Apl2_12580 | 3.21E-05 | TIMP3 | Metalloproteinase inhibitor 3 |

***Table S14. MHC region differences between the goose,*** ***duck, and chicken genomes.***

| **Type** | | **MHC** | | |
| --- | --- | --- | --- | --- |
|  |  | **Goose** | **Chicken** | **Duck** |
| Location | | Scaffold* | chr16 |  |
| Length (bp) | | 493,764 | 432,983 |  |
| GC percent (%) | | 53.74 | 53.3 |  |
| Gene density (%) | |  |  |  |
| Copy number | |  |  |  |
| Repeat (bp) | DNA | 45 | 25,869 |  |
|  | LINE | 57,451 | 114,021 |  |
|  | LTR | 13,719 | 234,066 |  |
|  | SINE | 0 | 803 |  |
|  | Tr | 61,600 | 48,755 |  |
|  | Unknown | 1,160 | 1,123 |  |
|  | Total | 74,631 | 236,483 |  |
| Repeat percentage (%) | | 15.11 | 54.62 |  |

The locations shown indicate the MHC distribution regions, wherein ‘Scaffold*’ includes scaffold 315, scaffold 565, scaffold 1044, scaffold 558, C19037983, scaffold 544, and scaffold 400. The lengths shown indicate the total lengths of the MHC regions. The GC percentages shown indicate the GC percentages in the MHC region. The repeat percentage reflects LINE, LTR, SINE, Tr, and unknown repetitive DNA sequences. Tr is located in the genome by TRF. The Tr repeat is a short and large-copy-number repeat. The total shown refers to all repeat length statistics, without overlap.

**Table S15. Gene alignment information for the goose and chicken genome in the MHC region.**

| Chicken | | | | Goose | | | | |
| --- | --- | --- | --- | --- | --- | --- | --- | --- |
| Gene | Strand | Length | Exon | Gene | Strand | Length | Exon | Location |
| ENSGALP00000040429 | - | 591 | 2 | ACY_011405 | + | 27588 | 16 | scaffold558 |
|  |  |  |  | ACY_011406 | + | 10356 | 19 | scaffold558 |
|  |  |  |  | ACY_011309 | - | 2372 | 6 | scaffold544 |
|  |  |  |  | ACY_011310 | + | 2830 | 9 | scaffold544 |
|  |  |  |  | ACY_011311 | + | 333 | 1 | scaffold544 |
| ENSGALP00000000138 | + | 744 | 3 | ACY_011312 | - | 10118 | 4 | scaffold544 |
| ENSGALP00000000233 | - | 1346 | 6 |  |  |  |  |  |
| ENSGALP00000000221 | - | 2196 | 6 |  |  |  |  |  |
| ENSGALP00000000217 | - | 1789 | 5 |  |  |  |  |  |
| ENSGALP00000000213 | - | 2087 | 4 |  |  |  |  |  |
| ENSGALP00000000193 | + | 1353 | 6 | ACY_011482 | - | 8872 | 8 | scaffold565 |
|  |  |  |  | ACY_011483 | - | 4648 | 2 | scaffold565 |
|  |  |  |  | ACY_011484 | - | 8001 | 2 | scaffold565 |
| ENSGALP00000000171 | - | 2398 | 5 | ACY_007807 | + | 4231 | 6 | scaffold315 |
| ENSGALP00000040405 | - | 9622 | 20 |  |  |  |  |  |
| ENSGALP00000000083 | - | 5499 | 7 | ACY_007805 | + | 5099 | 6 | scaffold315 |
| ENSGALP00000000146 | + | 3621 | 7 | ACY_007804 | - | 9950 | 9 | scaffold315 |
| ENSGALP00000040393 | - | 600 | 3 | ACY_007803 | + | 4103 | 7 | scaffold315 |
|  |  |  |  | ACY_007802 | + | 2084 | 2 | scaffold315 |
| ENSGALP00000000151 | + | 3503 | 6 | ACY_000075 | + | 2029 | 2 | C19037983 |
| ENSGALP00000000163 | + | 816 | 3 | ACY_009604 | + | 1385 | 3 | scaffold400 |
| ENSGALP00000000148 | + | 8351 | 7 | ACY_009605 | + | 8579 | 7 | scaffold400 |
| ENSGALP00000000108 | - | 3027 | 6 | ACY_009606 | - | 5295 | 7 | scaffold400 |
|  |  |  |  | ACY_009609 | + | 1886 | 2 | scaffold400 |
|  |  |  |  | ACY_009610 | - | 3801 | 4 | scaffold400 |
| ENSGALP00000019549 | + | 4749 | 7 | ACY_009611 | + | 4814 | 7 | scaffold400 |
|  |  |  |  | ACY_009615 | - | 288 | 1 | scaffold400 |
| ENSGALP00000030879 | - | 7164 | 15 |  |  |  |  |  |
| ENSGALP00000030881 | - | 3205 | 11 |  |  |  |  |  |
| ENSGALP00000040382 | - | 204 | 1 | ACY_000346 | + | 1012 | 1 | scaffold1044 |
| ENSGALP00000002067 | + | 1051 | 2 |  |  |  |  |  |
| ENSGALP00000040364 | + | 1872 | 6 |  |  |  |  |  |
| ENSGALP00000021998 | - | 2058 | 8 |  |  |  |  |  |
| ENSGALP00000028313 | + | 1739 | 5 |  |  |  |  |  |
| ENSGALP00000040357 | - | 1547 | 5 |  |  |  |  |  |
| ENSGALP00000009984 | + | 2064 | 8 |  |  |  |  |  |
| ENSGALP00000023473 | + | 1285 | 4 |  |  |  |  |  |
| ENSGALP00000004106 | + | 1520 | 5 |  |  |  |  |  |
| ENSGALP00000000265 | + | 90 | 1 |  |  |  |  |  |
| ENSGALP00000008911 | + | 823 | 3 |  |  |  |  |  |
| ENSGALP00000028756 | - | 366 | 2 |  |  |  |  |  |
| ENSGALP00000040348 | + | 686 | 4 |  |  |  |  |  |
| ENSGALP00000040347 | - | 999 | 1 |  |  |  |  |  |

MHC gene information is displayed relative to the position on chr16. In the MHC region of chicken Chr16, we identified 56 genes, in which 33 genes were major histocompatibility complex-related genes. Seven goose scaffolds had 41 genes, 23 of which were major histocompatibility complex-related genes. The best alignment of the goose and chicken MHC gene regions is shown, with blank entries representing the absence of homologous genes between the genomes. Strand, alignment strand information; Length, gene length; Exon, exon number in each gene; Location, MHC gene location on the indicated goose scaffolds.

**Table S16. Copy-number variations of innate immunity genes.**

| **Gene family** | **Goose** | **Turkey** | **Chicken** | **Zebra finch** | **Human** | **Lizard** | **Description** |
| --- | --- | --- | --- | --- | --- | --- | --- |
| ABCA1 | 2 | 3 | 2 | 2 | 3 | 2 | ATP-binding cassette, sub-family A (ABC1), member 1 |
| ABCG1 | 21 | 26 | 25 | 30 | 39 | 24 | ATP-binding cassette, sub-family G (WHITE), member 1 |
| ACAP1 | 31 | 37 | 43 | 42 | 60 | 37 | ArfGAP with coiled-coil, ankyrin repeat and PH domains 1 |
| ADAM10 | 14 | 14 | 14 | 14 | 20 | 22 | ADAM metallopeptidase domain 10 |
| ADAR | 4 | 5 | 6 | 7 | 6 | 8 | Adenosine deaminase, RNA-specific |
| ADIPOQ | 38 | 35 | 36 | 27 | 38 | 41 | Adiponectin, C1Q and collagen domain containing |
| ADRB2 | 41 | 40 | 42 | 39 | 45 | 43 | Adrenergic, beta-2-, receptor, surface |
| AGER | 0 | 0 | 0 | 0 | 7 | 0 | Advanced glycosylation end product-specific receptor |
| AIM2 | 0 | 0 | 0 | 0 | 2 | 0 | Absent in melanoma 2 |
| AIMP1 | 2 | 2 | 2 | 2 | 2 | 3 | Aminoacyl tRNA synthetase complex-interacting multifunctional protein 1 |
| AIRE | 13 | 18 | 17 | 17 | 26 | 16 | Autoimmune regulator |
| AKAP10 | 1 | 1 | 1 | 2 | 1 | 1 | A kinase (PRKA) anchor protein 10 |
| AKIRIN2 | 1 | 1 | 1 | 1 | 2 | 1 | Akirin 2 |
| AKNA | 0 | 1 | 0 | 0 | 1 | 1 | AT-hook transcription factor |
| ANXA4 | 9 | 13 | 11 | 15 | 14 | 9 | Annexin A4 |
| AP3B1 | 5 | 5 | 5 | 5 | 5 | 4 | Adaptor-related protein complex 3, beta 1 subunit |
| APCS | 7 | 8 | 7 | 7 | 9 | 13 | Amyloid P component, serum |
| APOA1 | 1 | 3 | 4 | 1 | 3 | 2 | Apolipoprotein A-I |
| APOBEC3A | 2 | 2 | 2 | 4 | 10 | 3 | Apolipoprotein B mRNA editing enzyme, catalytic polypeptide-like 3A |
| APOBEC3B | 2 | 2 | 2 | 4 | 10 | 3 | Apolipoprotein B mRNA editing enzyme, catalytic polypeptide-like 3B |
| APOBEC3G | 2 | 2 | 2 | 4 | 10 | 3 | Apolipoprotein B mRNA editing enzyme, catalytic polypeptide-like 3G |
| APOH | 11 | 14 | 16 | 17 | 35 | 16 | Apolipoprotein H (beta-2-glycoprotein I) |
| APOL1 | 0 | 0 | 0 | 0 | 6 | 2 | Apolipoprotein L, 1 |
| APP | 3 | 2 | 2 | 2 | 3 | 4 | Amyloid beta (A4) precursor protein |
| AQP3 | 7 | 7 | 5 | 5 | 9 | 10 | Aquaporin 3 (Gill blood group) |
| ARF6 | 34 | 37 | 42 | 41 | 52 | 44 | ADP-ribosylation factor 6 |
| ARRB2 | 2 | 2 | 2 | 3 | 4 | 2 | Arrestin, beta 2 |
| ATF1 | 2 | 4 | 5 | 4 | 9 | 4 | Activating transcription factor 1 |
| ATF2 | 2 | 2 | 2 | 3 | 3 | 3 | Activating transcription factor 2 |
| ATF3 | 4 | 7 | 8 | 9 | 12 | 9 | Activating transcription factor 3 |
| ATG12 | 0 | 0 | 1 | 1 | 1 | 1 | ATG12 autophagy related 12 homolog (S. cerevisiae) |
| ATG16L1 | 30 | 43 | 43 | 40 | 51 | 43 | ATG16 autophagy related 16-like 1 (S. cerevisiae) |
| ATG5 | 1 | 1 | 1 | 1 | 1 | 1 | ATG5 autophagy related 5 homolog (S. cerevisiae) |
| AVP | 0 | 2 | 2 | 2 | 2 | 0 | Arginine vasopressin |
| AXL | 31 | 40 | 44 | 42 | 53 | 40 | AXL receptor tyrosine kinase |
| AZI2 | 1 | 1 | 1 | 1 | 2 | 2 | 5-azacytidine induced 2 |
| Adrb2 | 41 | 42 | 43 | 37 | 44 | 43 | Adrenergic receptor, beta 2 |
| Ahr | 12 | 19 | 18 | 17 | 19 | 18 | Aryl-hydrocarbon receptor |
| Aim2 | 0 | 0 | 0 | 0 | 4 | 0 | Absent in melanoma 2 |
| Aimp1 | 2 | 2 | 2 | 2 | 2 | 3 | Aminoacyl tRNA synthetase complex-interacting multifunctional protein 1 |
| Akap10 | 1 | 1 | 1 | 2 | 1 | 1 | A kinase (PRKA) anchor protein 10 |
| Akna | 0 | 1 | 0 | 0 | 1 | 1 | AT-hook transcription factor |
| Akt1 | 34 | 39 | 39 | 43 | 53 | 42 | Thymoma viral proto-oncogene 1 |
| Ap3b1 | 5 | 6 | 6 | 6 | 6 | 5 | Adaptor-related protein complex 3, beta 1 subunit |
| Apcs | 6 | 8 | 7 | 7 | 9 | 13 | Serum amyloid P-component |
| Apoh | 9 | 15 | 18 | 16 | 38 | 16 | Apolipoprotein H |
| Arf6 | 34 | 37 | 42 | 41 | 52 | 44 | ADP-ribosylation factor 6 |
| Arhgap15 | 1 | 1 | 2 | 2 | 3 | 3 | Rho GTPase activating protein 15 |
| Atg12 | 0 | 0 | 1 | 1 | 1 | 1 | Autophagy-related 12 (yeast) |
| Atg5 | 1 | 1 | 1 | 1 | 1 | 1 | Autophagy-related 5 (yeast) |
| Avp | 0 | 2 | 2 | 2 | 2 | 0 | Arginine vasopressin |
| BCAR1 | 3 | 2 | 3 | 3 | 4 | 3 | Breast cancer anti-estrogen resistance 1 |
| BCL10 | 1 | 1 | 1 | 1 | 1 | 1 | B-cell CLL/lymphoma 10 |
| BCL2 | 2 | 3 | 4 | 3 | 8 | 6 | B-cell CLL/lymphoma 2 |
| BCL2A1 | 1 | 3 | 2 | 4 | 4 | 4 | BCL2-related protein A1 |
| BCL2L1 | 4 | 4 | 5 | 5 | 9 | 7 | BCL2-like 1 |
| BCL3 | 36 | 38 | 40 | 34 | 60 | 42 | B-cell CLL/lymphoma 3 |
| BDKRB2 | 40 | 36 | 45 | 38 | 47 | 44 | Bradykinin receptor B2 |
| BECN1 | 1 | 1 | 1 | 1 | 1 | 1 | Beclin 1, autophagy related |
| BID | 1 | 1 | 1 | 1 | 1 | 1 | BH3 interacting domain death agonist |
| BIRC2 | 3 | 5 | 6 | 7 | 7 | 5 | Baculoviral IAP repeat containing 2 |
| BIRC5 | 2 | 2 | 3 | 4 | 3 | 2 | Baculoviral IAP repeat containing 5 |
| BPIFA1 | 0 | 0 | 0 | 0 | 4 | 0 | BPI fold containing family A, member 1 |
| BPIFB3 | 5 | 6 | 8 | 3 | 7 | 5 | BPI fold containing family B, member 3 |
| BST2 | 0 | 0 | 0 | 0 | 1 | 0 | Bone marrow stromal cell antigen 2 |
| BTK | 5 | 5 | 5 | 5 | 5 | 4 | Bruton agammaglobulinemia tyrosine kinase |
| BTN3A2 | 17 | 12 | 24 | 3 | 32 | 4 | Butyrophilin, subfamily 3, member A2 |
| BTN3A3 | 30 | 20 | 29 | 9 | 105 | 57 | Butyrophilin, subfamily 3, member A3 |
| Bcl10 | 1 | 1 | 1 | 1 | 1 | 1 | B-cell leukemia/lymphoma 10 |
| Bcl2 | 3 | 3 | 4 | 5 | 9 | 7 | B-cell leukemia/lymphoma 2 |
| Bcl3 | 39 | 41 | 37 | 35 | 55 | 43 | B-cell leukemia/lymphoma 3 |
| Birc2 | 11 | 11 | 13 | 12 | 9 | 8 | Baculoviral IAP repeat-containing 2 |
| Birc3 | 8 | 9 | 10 | 9 | 9 | 8 | Baculoviral IAP repeat-containing 3 |
| Bst2 | 0 | 0 | 0 | 0 | 1 | 0 | Bone marrow stromal cell antigen 2 |
| C19orf29 | 1 | 1 | 0 | 1 | 1 | 1 | Chromosome 19 open reading frame 29 |
| C1QA | 0 | 0 | 0 | 0 | 1 | 0 | Complement component 1, q subcomponent, A chain |
| C1QB | 40 | 37 | 43 | 32 | 55 | 43 | Complement component 1, q subcomponent, B chain |
| C1QBP | 1 | 0 | 1 | 1 | 1 | 1 | Complement component 1, q subcomponent binding protein |
| C1QC | 36 | 36 | 43 | 33 | 59 | 43 | Complement component 1, q subcomponent, C chain |
| C1QTNF3 | 18 | 15 | 18 | 17 | 26 | 22 | C1q and tumor necrosis factor related protein 3 |
| C1RL | 24 | 35 | 35 | 38 | 64 | 45 | Complement component 1, r subcomponent-like |
| C1S | 28 | 38 | 39 | 38 | 59 | 48 | Complement component 1, s subcomponent |
| C1qb | 39 | 38 | 39 | 34 | 54 | 46 | Complement component 1, q subcomponent, beta polypeptide |
| C1ra | 23 | 38 | 37 | 37 | 63 | 52 | Complement component 1, r subcomponent A |
| C1rb | 23 | 37 | 37 | 37 | 65 | 51 | Complement component 1, r subcomponent B |
| C1rl | 26 | 36 | 36 | 35 | 65 | 52 | Complement component 1, r subcomponent-like |
| C1s | 2 | 2 | 4 | 3 | 2 | 2 | Complement component 1, s subcomponent |
| C2 | 22 | 29 | 28 | 29 | 64 | 36 | Complement component 2 |
| C3 | 13 | 14 | 12 | 15 | 16 | 14 | Complement component 3 |
| C4BPA | 0 | 0 | 0 | 0 | 1 | 0 | Complement component 4 binding protein, alpha |
| C4BPB | 6 | 11 | 12 | 10 | 119 | 12 | Complement component 4 binding protein, beta |
| C4b | 2 | 3 | 1 | 1 | 10 | 4 | Complement component 4B (Childo blood group) |
| C4bp | 11 | 13 | 17 | 16 | 23 | 17 | Complement component 4 binding protein |
| C5 | 12 | 12 | 12 | 13 | 16 | 14 | Complement component 5 |
| C5AR1 | 40 | 36 | 44 | 37 | 43 | 50 | Complement component 5a receptor 1 |
| C6 | 0 | 0 | 0 | 0 | 1 | 0 | Complement component 6 |
| C7 | 4 | 4 | 4 | 5 | 6 | 7 | Complement component 7 |
| C8A | 13 | 13 | 17 | 14 | 18 | 20 | Complement component 8, alpha polypeptide |
| C8B | 10 | 8 | 10 | 12 | 10 | 16 | Complement component 8, beta polypeptide |
| C8a | 9 | 9 | 10 | 9 | 10 | 13 | Complement component 8, alpha polypeptide |
| C9 | 6 | 7 | 5 | 8 | 7 | 11 | Complement component 9 |
| CALCA | 1 | 1 | 1 | 1 | 2 | 1 | Calcitonin-related polypeptide alpha |
| CAMP | 0 | 0 | 1 | 0 | 1 | 2 | Cathelicidin antimicrobial peptide |
| CAPZA2 | 3 | 3 | 3 | 3 | 3 | 4 | Capping protein (actin filament) muscle Z-line, alpha 2 |
| CARD6 | 0 | 0 | 0 | 0 | 3 | 0 | Caspase recruitment domain family, member 6 |
| CARD9 | 3 | 3 | 3 | 4 | 4 | 4 | Caspase recruitment domain family, member 9 |
| CASP1 | 9 | 10 | 9 | 9 | 15 | 9 | Caspase 1, apoptosis-related cysteine peptidase (interleukin 1, beta, convertase) |
| CASP10 | 2 | 3 | 3 | 2 | 3 | 1 | Caspase 10, apoptosis-related cysteine peptidase |
| CASP12 | 8 | 8 | 8 | 8 | 12 | 8 | Caspase 12 (gene/pseudogene) |
| CASP6 | 1 | 1 | 1 | 2 | 1 | 1 | Caspase 6, apoptosis-related cysteine peptidase |
| CASP7 | 9 | 11 | 11 | 11 | 14 | 10 | Caspase 7, apoptosis-related cysteine peptidase |
| CASP8 | 2 | 4 | 4 | 3 | 3 | 2 | Caspase 8, apoptosis-related cysteine peptidase |
| CAV1 | 2 | 2 | 3 | 3 | 3 | 4 | Caveolin 1, caveolae protein, 22kDa |
| CBL | 3 | 3 | 1 | 3 | 3 | 3 | Cas-Br-M (murine) ecotropic retroviral transforming sequence |
| CCBP2 | 41 | 34 | 41 | 41 | 46 | 47 | Chemokine binding protein 2 |
| CCDC88A | 2 | 2 | 2 | 2 | 2 | 2 | Coiled-coil domain containing 88A |
| CCL1 | 3 | 3 | 3 | 2 | 11 | 0 | Chemokine (C-C motif) ligand 1 |
| CCL17 | 4 | 3 | 4 | 6 | 17 | 0 | Chemokine (C-C motif) ligand 17 |
| CCL2 | 4 | 6 | 8 | 5 | 18 | 0 | Chemokine (C-C motif) ligand 2 |
| CCR3 | 45 | 36 | 42 | 39 | 47 | 41 | Chemokine (C-C motif) receptor 3 |
| CCR4 | 41 | 36 | 41 | 40 | 47 | 45 | Chemokine (C-C motif) receptor 4 |
| CCR6 | 40 | 37 | 44 | 42 | 45 | 42 | Chemokine (C-C motif) receptor 6 |
| CCR7 | 39 | 37 | 41 | 40 | 47 | 46 | Chemokine (C-C motif) receptor 7 |
| CD14 | 1 | 2 | 1 | 2 | 1 | 3 | CD14 molecule |
| CD200 | 1 | 2 | 1 | 2 | 1 | 2 | CD200 molecule |
| CD209 | 0 | 0 | 0 | 0 | 1 | 0 | CD209 molecule |
| CD22 | 30 | 35 | 35 | 35 | 75 | 40 | CD22 molecule |
| CD274 | 1 | 1 | 2 | 1 | 3 | 2 | CD274 molecule |
| CD300A | 0 | 0 | 0 | 0 | 1 | 0 | CD300a molecule |
| CD300E | 3 | 4 | 6 | 3 | 14 | 4 | CD300e molecule |
| CD300LF | 3 | 5 | 4 | 2 | 10 | 4 | CD300 molecule-like family member f |
| CD36 | 3 | 3 | 3 | 4 | 3 | 3 | CD36 molecule (thrombospondin receptor) |
| CD37 | 8 | 9 | 9 | 11 | 16 | 16 | CD37 molecule |
| CD46 | 14 | 19 | 19 | 23 | 37 | 19 | CD46 molecule, complement regulatory protein |
| CD53 | 3 | 9 | 13 | 10 | 17 | 14 | CD53 molecule |
| CD55 | 1 | 1 | 2 | 1 | 1 | 1 | CD55 molecule, decay accelerating factor for complement (Cromer blood group) |
| CD63 | 8 | 10 | 14 | 15 | 17 | 13 | CD63 molecule |
| CD8A | 1 | 1 | 1 | 1 | 1 | 0 | CD8a molecule |
| CD97 | 33 | 41 | 41 | 37 | 57 | 41 | CD97 molecule |
| CDK1 | 34 | 38 | 38 | 39 | 53 | 48 | Cyclin-dependent kinase 1 |
| CDK9 | 29 | 38 | 40 | 45 | 52 | 46 | Cyclin-dependent kinase 9 |
| CDKN2A | 0 | 0 | 0 | 0 | 1 | 0 | Cyclin-dependent kinase inhibitor 2A (melanoma, p16, inhibits CDK4) |
| CEACAM1 | 30 | 33 | 37 | 30 | 74 | 46 | Carcinoembryonic antigen-related cell adhesion molecule 1 (biliary glycoprotein) |
| CEBPA | 1 | 1 | 3 | 4 | 4 | 5 | CCAAT/enhancer binding protein (C/EBP), alpha |
| CEBPB | 1 | 1 | 3 | 4 | 4 | 5 | CCAAT/enhancer binding protein (C/EBP), beta |
| CEBPD | 0 | 0 | 0 | 1 | 1 | 1 | CCAAT/enhancer binding protein (C/EBP), delta |
| CEBPE | 1 | 1 | 3 | 4 | 4 | 5 | CCAAT/enhancer binding protein (C/EBP), epsilon |
| CFB | 28 | 34 | 34 | 33 | 74 | 47 | Complement factor B |
| CFD | 18 | 35 | 35 | 32 | 80 | 50 | Complement factor D (adipsin) |
| CFH | 9 | 16 | 17 | 17 | 26 | 17 | Complement factor H |
| CFI | 15 | 17 | 16 | 15 | 16 | 15 | Complement factor I |
| CFLAR | 8 | 8 | 9 | 9 | 9 | 6 | CASP8 and FADD-like apoptosis regulator |
| CFP | 24 | 20 | 20 | 22 | 24 | 21 | Complement factor properdin |
| CFTR | 22 | 26 | 26 | 30 | 47 | 29 | Cystic fibrosis transmembrane conductance regulator (ATP-binding cassette sub-family C, member 7) |
| CHID1 | 1 | 1 | 1 | 1 | 3 | 1 | Chitinase domain containing 1 |
| CISH | 10 | 7 | 11 | 11 | 11 | 12 | Cytokine inducible SH2-containing protein |
| CLEC10A | 19 | 20 | 28 | 20 | 56 | 43 | C-type lectin domain family 10, member A |
| CLEC1B | 10 | 8 | 16 | 7 | 31 | 19 | C-type lectin domain family 1, member B |
| CLEC4A | 0 | 0 | 0 | 0 | 1 | 0 | C-type lectin domain family 4, member A |
| CLEC4C | 14 | 12 | 20 | 14 | 37 | 34 | C-type lectin domain family 4, member C |
| CLEC4D | 17 | 15 | 21 | 18 | 35 | 40 | C-type lectin domain family 4, member D |
| CLEC4M | 1 | 0 | 0 | 1 | 4 | 0 | C-type lectin domain family 4, member M |
| CLEC6A | 11 | 11 | 16 | 12 | 37 | 32 | C-type lectin domain family 6, member A |
| CLEC7A | 0 | 0 | 0 | 0 | 1 | 0 | C-type lectin domain family 7, member A |
| CLU | 1 | 2 | 2 | 1 | 2 | 2 | Clusterin |
| CNOT4 | 1 | 1 | 1 | 1 | 1 | 1 | CCR4-NOT transcription complex, subunit 4 |
| CNPY3 | 0 | 0 | 0 | 0 | 1 | 0 | Canopy 3 homolog (zebrafish) |
| COLEC12 | 34 | 34 | 32 | 30 | 66 | 54 | Collectin sub-family member 12 |
| CORO1A | 20 | 27 | 25 | 32 | 36 | 26 | Coronin, actin binding protein, 1A |
| CORO2A | 15 | 19 | 17 | 18 | 25 | 18 | Coronin, actin binding protein, 2A |
| CR2 | 14 | 18 | 22 | 21 | 43 | 21 | Complement component (3d/Epstein Barr virus) receptor 2 |
| CRCP | 0 | 1 | 1 | 1 | 2 | 0 | CGRP receptor component |
| CREB1 | 2 | 3 | 3 | 3 | 3 | 2 | cAMP responsive element binding protein 1 |
| CREBBP | 2 | 2 | 2 | 2 | 2 | 2 | CREB binding protein |
| CRISP3 | 5 | 9 | 8 | 10 | 14 | 16 | Cysteine-rich secretory protein 3 |
| CRKL | 20 | 25 | 28 | 23 | 27 | 30 | v-crk sarcoma virus CT10 oncogene homolog (avian)-like |
| CRP | 0 | 0 | 0 | 0 | 2 | 0 | C-reactive protein, pentraxin-related |
| CSF2RB | 4 | 2 | 4 | 3 | 3 | 1 | Colony stimulating factor 2 receptor, beta, low-affinity (granulocyte-macrophage) |
| CSK | 35 | 39 | 45 | 43 | 49 | 39 | c-src tyrosine kinase |
| CTCF | 4 | 6 | 7 | 11 | 117 | 105 | CCCTC-binding factor (zinc finger protein) |
| CTLA4 | 0 | 0 | 0 | 0 | 1 | 0 | Cytotoxic T-lymphocyte-associated protein 4 |
| CTNNAL1 | 5 | 6 | 4 | 5 | 4 | 5 | Catenin (cadherin-associated protein), alpha-like 1 |
| CTSB | 1 | 1 | 1 | 1 | 1 | 1 | Cathepsin B |
| CTSD | 9 | 8 | 8 | 8 | 11 | 11 | Cathepsin D |
| CTSG | 18 | 35 | 35 | 29 | 83 | 50 | Cathepsin G |
| CTSH | 2 | 2 | 3 | 2 | 4 | 3 | Cathepsin H |
| CTSK | 7 | 10 | 9 | 7 | 9 | 9 | Cathepsin K |
| CTSL1 | 0 | 0 | 0 | 0 | 2 | 0 | Cathepsin L1 |
| CTSS | 10 | 12 | 12 | 14 | 13 | 14 | Cathepsin S |
| CXCL10 | 1 | 2 | 2 | 2 | 4 | 1 | Chemokine (C-X-C motif) ligand 10 |
| CXCL11 | 0 | 0 | 0 | 0 | 3 | 1 | Chemokine (C-X-C motif) ligand 11 |
| CXCL9 | 1 | 2 | 2 | 2 | 9 | 1 | Chemokine (C-X-C motif) ligand 9 |
| CXCR3 | 44 | 36 | 43 | 38 | 46 | 43 | Chemokine (C-X-C motif) receptor 3 |
| CXCR4 | 43 | 32 | 41 | 38 | 47 | 49 | Chemokine (C-X-C motif) receptor 4 |
| CYBA | 0 | 1 | 0 | 1 | 1 | 1 | Cytochrome b-245, alpha polypeptide |
| CYBB | 6 | 6 | 6 | 6 | 7 | 5 | Cytochrome b-245, beta polypeptide |
| CYLD | 2 | 2 | 2 | 2 | 1 | 5 | Cylindromatosis (turban tumor syndrome) |
| CYTIP | 2 | 2 | 2 | 2 | 5 | 3 | Cytohesin 1 interacting protein |
| Camk2a | 3 | 4 | 3 | 5 | 4 | 4 | Calcium/calmodulin-dependent protein kinase II alpha |
| Camp | 0 | 1 | 1 | 0 | 1 | 2 | Cathelicidin antimicrobial peptide |
| Card9 | 3 | 3 | 3 | 4 | 4 | 4 | Caspase recruitment domain family, member 9 |
| Casp1 | 9 | 9 | 9 | 9 | 15 | 9 | Caspase 1 |
| Casp8 | 9 | 11 | 11 | 11 | 14 | 10 | Caspase 8 |
| Cbl | 2 | 2 | 1 | 2 | 2 | 2 | Casitas B-lineage lymphoma |
| Ccbp2 | 45 | 32 | 39 | 42 | 48 | 44 | Chemokine binding protein 2 |
| Ccdc88a | 2 | 2 | 3 | 2 | 3 | 3 | Coiled coil domain containing 88A |
| Ccl17 | 2 | 3 | 5 | 4 | 16 | 0 | Chemokine (C-C motif) ligand 17 |
| Ccr3 | 43 | 37 | 41 | 40 | 41 | 48 | Chemokine (C-C motif) receptor 3 |
| Ccr6 | 42 | 36 | 41 | 43 | 45 | 43 | Chemokine (C-C motif) receptor 6 |
| Cd14 | 1 | 2 | 3 | 3 | 2 | 4 | CD14 antigen |
| Cd1d2 | 4 | 4 | 15 | 1 | 20 | 9 | CD1d2 antigen |
| Cd209a | 21 | 20 | 30 | 23 | 57 | 46 | CD209a antigen |
| Cd300lf | 4 | 6 | 5 | 3 | 13 | 4 | CD300 antigen like family member F |
| Cd46 | 10 | 16 | 18 | 18 | 22 | 19 | CD46 antigen, complement regulatory protein |
| Cd5l | 16 | 25 | 26 | 18 | 17 | 18 | CD5 antigen-like |
| Cd8a | 1 | 2 | 1 | 1 | 1 | 0 | CD8 antigen, alpha chain |
| Cd97 | 32 | 45 | 43 | 38 | 55 | 37 | CD97 antigen |
| Cdk6 | 30 | 41 | 40 | 36 | 53 | 50 | Cyclin-dependent kinase 6 |
| Cebpa | 1 | 1 | 3 | 4 | 4 | 5 | CCAAT/enhancer binding protein (C/EBP), alpha |
| Cebpb | 1 | 1 | 4 | 4 | 5 | 4 | CCAAT/enhancer binding protein (C/EBP), beta |
| Cebpe | 1 | 1 | 3 | 4 | 5 | 5 | CCAAT/enhancer binding protein (C/EBP), epsilon |
| Cfb | 24 | 33 | 34 | 33 | 61 | 46 | Complement factor B |
| Cfd | 22 | 31 | 34 | 30 | 83 | 50 | Complement factor D (adipsin) |
| Cfi | 25 | 35 | 38 | 36 | 66 | 50 | Complement component factor i |
| Cftr | 11 | 11 | 11 | 10 | 14 | 14 | Cystic fibrosis transmembrane conductance regulator homolog |
| Chga | 1 | 1 | 1 | 1 | 2 | 1 | Chromogranin A |
| Chid1 | 1 | 1 | 1 | 1 | 1 | 1 | Chitinase domain containing 1 |
| Chuk | 27 | 33 | 38 | 52 | 59 | 41 | Conserved helix-loop-helix ubiquitous kinase |
| Clec4a2 | 11 | 8 | 8 | 13 | 31 | 21 | C-type lectin domain family 4, member a2 |
| Clec4d | 14 | 11 | 17 | 14 | 34 | 33 | C-type lectin domain family 4, member d |
| Clec4e | 17 | 15 | 24 | 16 | 42 | 46 | C-type lectin domain family 4, member e |
| Clec5a | 9 | 7 | 13 | 2 | 33 | 16 | C-type lectin domain family 5, member a |
| Clec7a | 10 | 6 | 14 | 3 | 35 | 14 | C-type lectin domain family 7, member a |
| Cltc | 1 | 2 | 2 | 3 | 2 | 2 | Clathrin, heavy polypeptide (Hc) |
| Cnot4 | 1 | 1 | 1 | 1 | 1 | 1 | CCR4-NOT transcription complex, subunit 4 |
| Coro2a | 16 | 18 | 16 | 18 | 26 | 17 | Coronin, actin binding protein 2A |
| Cr1l | 11 | 16 | 18 | 20 | 39 | 19 | Complement component (3b/4b) receptor 1-like |
| Crp | 6 | 8 | 7 | 7 | 9 | 13 | C-reactive protein, pentraxin-related |
| Ctsb | 10 | 10 | 12 | 13 | 12 | 13 | Cathepsin B |
| Ctss | 10 | 12 | 11 | 14 | 13 | 14 | Cathepsin S |
| Cxcl2 | 1 | 2 | 5 | 4 | 9 | 1 | Chemokine (C-X-C motif) ligand 2 |
| Cxcl9 | 1 | 2 | 2 | 2 | 9 | 1 | Chemokine (C-X-C motif) ligand 9 |
| Cxcr3 | 40 | 33 | 42 | 42 | 46 | 47 | Chemokine (C-X-C motif) receptor 3 |
| Cyba | 0 | 1 | 0 | 1 | 1 | 1 | Cytochrome b-245, alpha polypeptide |
| Cyld | 2 | 2 | 2 | 1 | 1 | 2 | Cylindromatosis (turban tumor syndrome) |
| DAB2IP | 8 | 10 | 8 | 11 | 12 | 8 | DAB2 interacting protein |
| DCD | 0 | 0 | 0 | 0 | 1 | 0 | Dermcidin |
| DCN | 24 | 18 | 21 | 15 | 34 | 30 | Decorin |
| DDIT3 | 0 | 0 | 0 | 0 | 1 | 1 | DNA-damage-inducible transcript 3 |
| DDOST | 1 | 1 | 1 | 2 | 1 | 1 | Dolichyl-diphosphooligosaccharide--protein glycosyltransferase |
| DDX1 | 30 | 30 | 31 | 36 | 50 | 36 | DEAD (Asp-Glu-Ala-Asp) box polypeptide 1 |
| DDX21 | 0 | 0 | 0 | 0 | 1 | 0 | DEAD (Asp-Glu-Ala-Asp) box polypeptide 21 |
| DDX3X | 2 | 1 | 2 | 2 | 3 | 2 | DEAD (Asp-Glu-Ala-Asp) box polypeptide 3, X-linked |
| DDX58 | 1 | 0 | 0 | 1 | 1 | 1 | DEAD (Asp-Glu-Ala-Asp) box polypeptide 58 |
| DEFA1 | 0 | 0 | 0 | 0 | 6 | 0 | Defensin, alpha 1 |
| DEFA4 | 0 | 0 | 0 | 0 | 6 | 0 | Defensin, alpha 4, corticostatin |
| DEFA5 | 0 | 0 | 0 | 0 | 6 | 0 | Defensin, alpha 5, Paneth cell-specific |
| DEFA6 | 0 | 0 | 0 | 0 | 6 | 0 | Defensin, alpha 6, Paneth cell-specific |
| DEFB1 | 0 | 0 | 0 | 0 | 1 | 0 | Defensin, beta 1 |
| DEFB103A | 0 | 0 | 0 | 0 | 2 | 0 | Defensin, beta 103A |
| DEFB103B | 0 | 0 | 0 | 0 | 2 | 0 | Defensin, beta 103B |
| DEFB118 | 0 | 0 | 0 | 0 | 3 | 0 | Defensin, beta 118 |
| DEFB127 | 0 | 0 | 0 | 0 | 2 | 0 | Defensin, beta 127 |
| DEFB4A | 0 | 0 | 0 | 0 | 2 | 0 | Defensin, beta 4A |
| DHCR24 | 1 | 1 | 1 | 1 | 1 | 1 | 24-dehydrocholesterol reductase |
| DHX36 | 16 | 16 | 15 | 25 | 25 | 18 | DEAH (Asp-Glu-Ala-His) box polypeptide 36 |
| DHX58 | 5 | 4 | 4 | 5 | 5 | 5 | DEXH (Asp-Glu-X-His) box polypeptide 58 |
| DHX9 | 15 | 15 | 15 | 17 | 25 | 17 | DEAH (Asp-Glu-Ala-His) box polypeptide 9 |
| DLK1 | 5 | 7 | 10 | 10 | 12 | 9 | Delta-like 1 homolog (Drosophila) |
| DMBT1 | 34 | 45 | 48 | 45 | 39 | 39 | Deleted in malignant brain tumors 1 |
| DUOX1 | 23 | 21 | 22 | 26 | 28 | 28 | Dual oxidase 1 |
| DUSP1 | 24 | 26 | 26 | 24 | 27 | 30 | Dual specificity phosphatase 1 |
| DUSP16 | 3 | 3 | 4 | 4 | 4 | 3 | Dual specificity phosphatase 16 |
| DUSP3 | 22 | 24 | 24 | 23 | 25 | 29 | Dual specificity phosphatase 3 |
| DUSP4 | 24 | 26 | 26 | 24 | 27 | 30 | Dual specificity phosphatase 4 |
| DUSP6 | 2 | 1 | 3 | 4 | 4 | 3 | Dual specificity phosphatase 6 |
| DUSP7 | 24 | 26 | 26 | 24 | 27 | 30 | Dual specificity phosphatase 7 |
| Daf2 | 12 | 18 | 21 | 20 | 32 | 19 | Decay accelerating factor 2 |
| Dcn | 36 | 36 | 43 | 38 | 52 | 45 | Decorin |
| Ddx1 | 30 | 30 | 31 | 36 | 50 | 35 | DEAD (Asp-Glu-Ala-Asp) box polypeptide 1 |
| Ddx21 | 30 | 30 | 30 | 36 | 46 | 34 | DEAD (Asp-Glu-Ala-Asp) box polypeptide 21 |
| Ddx41 | 29 | 29 | 30 | 36 | 45 | 34 | DEAD (Asp-Glu-Ala-Asp) box polypeptide 41 |
| Ddx58 | 4 | 3 | 3 | 5 | 5 | 4 | DEAD (Asp-Glu-Ala-Asp) box polypeptide 58 |
| Defa20 | 0 | 0 | 0 | 0 | 6 | 0 | Defensin, alpha, 20 |
| Defb1 | 0 | 0 | 0 | 0 | 1 | 0 | Defensin beta 1 |
| Defb14 | 0 | 0 | 0 | 0 | 2 | 0 | Defensin beta 14 |
| Dhx58 | 6 | 5 | 5 | 6 | 5 | 7 | DEXH (Asp-Glu-X-His) box polypeptide 58 |
| Dlk1 | 1 | 1 | 0 | 1 | 1 | 1 | Delta-like 1 homolog (Drosophila) |
| Duox2 | 1 | 1 | 1 | 1 | 3 | 1 | Dual oxidase 2 |
| E2F1 | 6 | 7 | 7 | 7 | 8 | 8 | E2F transcription factor 1 |
| E2f1 | 6 | 7 | 7 | 7 | 8 | 8 | E2F transcription factor 1 |
| ECSIT | 0 | 0 | 0 | 0 | 1 | 1 | ECSIT homolog (Drosophila) |
| EDN1 | 2 | 1 | 3 | 3 | 3 | 3 | Endothelin 1 |
| EGFR | 4 | 7 | 6 | 6 | 7 | 6 | Epidermal growth factor receptor |
| EGR1 | 1 | 1 | 1 | 1 | 1 | 1 | Early growth response 1 |
| EIF2AK2 | 1 | 2 | 2 | 2 | 2 | 1 | Eukaryotic translation initiation factor 2-alpha kinase 2 |
| ELANE | 23 | 35 | 38 | 36 | 70 | 48 | Elastase, neutrophil expressed |
| ELAVL1 | 32 | 33 | 40 | 43 | 58 | 44 | ELAV (embryonic lethal, abnormal vision, Drosophila)-like 1 (Hu antigen R) |
| ELF1 | 4 | 4 | 4 | 4 | 6 | 4 | E74-like factor 1 (ets domain transcription factor) |
| ELK1 | 17 | 21 | 21 | 18 | 27 | 24 | ELK1, member of ETS oncogene family |
| EP300 | 18 | 23 | 22 | 18 | 23 | 21 | E1A binding protein p300 |
| EPOR | 3 | 2 | 4 | 2 | 8 | 4 | Erythropoietin receptor |
| ERAP1 | 12 | 11 | 12 | 10 | 12 | 11 | Endoplasmic reticulum aminopeptidase 1 |
| ERBB2IP | 40 | 40 | 43 | 41 | 48 | 38 | erbb2 interacting protein |
| ERN1 | 23 | 34 | 40 | 55 | 55 | 43 | Endoplasmic reticulum to nucleus signaling 1 |
| ETS1 | 15 | 21 | 20 | 17 | 27 | 23 | v-ets erythroblastosis virus E26 oncogene homolog 1 (avian) |
| Edn1 | 2 | 1 | 3 | 3 | 3 | 3 | Endothelin 1 |
| Elf1 | 1 | 1 | 1 | 1 | 2 | 1 | E74-like factor 1 |
| Erbb2ip | 41 | 39 | 45 | 40 | 46 | 39 | Erbb2 interacting protein |
| F11 | 27 | 37 | 40 | 37 | 68 | 50 | Coagulation factor XI |
| F2R | 0 | 0 | 0 | 0 | 1 | 0 | Coagulation factor II (thrombin) receptor |
| F2RL1 | 46 | 33 | 49 | 38 | 42 | 42 | Coagulation factor II (thrombin) receptor-like 1 |
| F2RL2 | 40 | 35 | 48 | 38 | 45 | 44 | Coagulation factor II (thrombin) receptor-like 2 |
| F2RL3 | 39 | 34 | 48 | 38 | 46 | 45 | Coagulation factor II (thrombin) receptor-like 3 |
| F2rl1 | 43 | 35 | 52 | 41 | 39 | 40 | Coagulation factor II (thrombin) receptor-like 1 |
| FADD | 3 | 2 | 2 | 3 | 1 | 1 | Fas (TNFRSF6)-associated via death domain |
| FANCC | 1 | 1 | 1 | 1 | 1 | 1 | Fanconi anemia, complementation group C |
| FBXW5 | 1 | 1 | 1 | 0 | 1 | 1 | F-box and WD repeat domain containing 5 |
| FCN1 | 24 | 28 | 27 | 22 | 32 | 33 | Ficolin (collagen/fibrinogen domain containing) 1 |
| FFAR2 | 36 | 30 | 66 | 36 | 38 | 44 | Free fatty acid receptor 2 |
| FLI1 | 15 | 21 | 20 | 19 | 28 | 23 | Friend leukemia virus integration 1 |
| FOS | 4 | 3 | 3 | 4 | 6 | 6 | FBJ murine osteosarcoma viral oncogene homolog |
| FOXA2 | 18 | 18 | 27 | 27 | 47 | 33 | Forkhead box A2 |
| FOXO3 | 3 | 3 | 3 | 3 | 4 | 4 | Forkhead box O3 |
| FREM1 | 3 | 2 | 3 | 3 | 4 | 3 | FRAS1 related extracellular matrix 1 |
| FSTL1 | 1 | 1 | 1 | 1 | 1 | 1 | Follistatin-like 1 |
| FZD1 | 17 | 18 | 19 | 20 | 19 | 19 | Frizzled family receptor 1 |
| Fadd | 3 | 2 | 3 | 1 | 2 | 3 | Fas (TNFRSF6)-associated via death domain |
| Fancc | 1 | 1 | 1 | 1 | 1 | 1 | Fanconi anemia, complementation group C |
| Fcgr1 | 0 | 1 | 1 | 0 | 18 | 1 | Fc receptor, IgG, high affinity I |
| Fcna | 23 | 26 | 27 | 22 | 29 | 29 | Ficolin A |
| Fcnb | 7 | 7 | 3 | 3 | 5 | 1 | Ficolin B |
| Fcrl5 | 14 | 14 | 20 | 14 | 35 | 19 | Fc receptor-like 5 |
| Fgf7 | 12 | 15 | 19 | 18 | 22 | 20 | Fibroblast growth factor 7 |
| Frem1 | 12 | 14 | 13 | 14 | 19 | 14 | Fras1 related extracellular matrix protein 1 |
| Fzd1 | 17 | 18 | 19 | 20 | 19 | 19 | Frizzled homolog 1 (Drosophila) |
| GAS6 | 4 | 2 | 3 | 3 | 5 | 5 | Growth arrest-specific 6 |
| GATA4 | 6 | 6 | 6 | 6 | 7 | 7 | GATA binding protein 4 |
| GATA6 | 6 | 6 | 6 | 6 | 7 | 7 | GATA binding protein 6 |
| GJA1 | 15 | 14 | 15 | 19 | 20 | 18 | Gap junction protein, alpha 1, 43kDa |
| GLI1 | 18 | 19 | 27 | 23 | 113 | 50 | GLI family zinc finger 1 |
| GLRX | 1 | 1 | 3 | 2 | 3 | 2 | Glutaredoxin (thioltransferase) |
| GNAI2 | 16 | 17 | 13 | 15 | 17 | 18 | Guanine nucleotide binding protein (G protein), alpha inhibiting activity polypeptide 2 |
| GNAI3 | 18 | 17 | 13 | 16 | 17 | 19 | Guanine nucleotide binding protein (G protein), alpha inhibiting activity polypeptide 3 |
| GOPC | 16 | 19 | 22 | 24 | 25 | 20 | Golgi-associated PDZ and coiled-coil motif containing |
| GP2 | 9 | 9 | 8 | 8 | 9 | 8 | Glycoprotein 2 (zymogen granule membrane) |
| GPR33 | 38 | 37 | 44 | 42 | 45 | 44 | G protein-coupled receptor 33 (gene/pseudogene) |
| GPR77 | 43 | 33 | 41 | 39 | 45 | 49 | G protein-coupled receptor 77 |
| GPSM1 | 2 | 2 | 2 | 2 | 2 | 1 | G-protein signaling modulator 1 |
| GRK5 | 34 | 39 | 38 | 45 | 54 | 40 | G protein-coupled receptor kinase 5 |
| GRN | 0 | 0 | 0 | 1 | 1 | 1 | Granulin |
| GSK3B | 1 | 1 | 1 | 1 | 2 | 2 | Glycogen synthase kinase 3 beta |
| GZMM | 24 | 33 | 35 | 32 | 80 | 46 | Granzyme M (lymphocyte met-ase 1) |
| Gas6 | 34 | 38 | 41 | 44 | 52 | 41 | Growth arrest specific 6 |
| Gata4 | 6 | 6 | 6 | 6 | 7 | 7 | GATA binding protein 4 |
| Gata6 | 6 | 6 | 6 | 6 | 7 | 7 | GATA binding protein 6 |
| Gbp1 | 3 | 7 | 6 | 2 | 10 | 5 | Guanylate binding protein 1 |
| Gbp10 | 3 | 7 | 6 | 2 | 10 | 6 | Guanylate binding protein 10 |
| Gbp2 | 1 | 4 | 3 | 0 | 7 | 2 | Guanylate binding protein 2 |
| Gbp6 | 2 | 5 | 4 | 0 | 7 | 2 | Guanylate binding protein 6 |
| Gbp7 | 3 | 7 | 6 | 2 | 10 | 6 | Guanylate binding protein 7 |
| Gm20547 | 26 | 35 | 33 | 33 | 76 | 47 | Predicted gene 20547 |
| Gm5077 | 25 | 37 | 40 | 37 | 62 | 49 | Predicted gene 5077 |
| Gnai2 | 17 | 17 | 13 | 15 | 17 | 18 | Guanine nucleotide binding protein (G protein), alpha inhibiting 2 |
| Gnai3 | 18 | 17 | 13 | 16 | 17 | 18 | Guanine nucleotide binding protein (G protein), alpha inhibiting 3 |
| Gp2 | 11 | 12 | 11 | 10 | 11 | 11 | Glycoprotein 2 (zymogen granule membrane) |
| Gpr33 | 38 | 38 | 41 | 40 | 45 | 48 | G protein-coupled receptor 33 |
| Gpr77 | 38 | 34 | 38 | 38 | 45 | 57 | G protein-coupled receptor 77 |
| Gpsm1 | 3 | 3 | 3 | 4 | 4 | 3 | G-protein signaling modulator 1 (AGS3-like, C. elegans) |
| H2-Aa | 4 | 4 | 12 | 11 | 97 | 17 | Histocompatibility 2, class II antigen A, alpha |
| H2-Ab1 | 7 | 9 | 22 | 19 | 141 | 30 | Histocompatibility 2, class II antigen A, beta 1 |
| HAMP | 0 | 0 | 0 | 0 | 1 | 0 | Hepcidin antimicrobial peptide |
| HDAC1 | 6 | 6 | 6 | 6 | 8 | 7 | Histone deacetylase 1 |
| HDAC2 | 8 | 9 | 10 | 9 | 11 | 10 | Histone deacetylase 2 |
| HIF1A | 12 | 13 | 12 | 13 | 13 | 13 | Hypoxia inducible factor 1, alpha subunit (basic helix-loop-helix transcription factor) |
| HMGB2 | 4 | 7 | 5 | 5 | 6 | 5 | High mobility group box 2 |
| HMGB3 | 14 | 15 | 12 | 18 | 21 | 17 | High mobility group box 3 |
| HOXA9 | 25 | 22 | 42 | 47 | 62 | 52 | Homeobox A9 |
| HRAS | 31 | 31 | 44 | 41 | 56 | 47 | v-Ha-ras Harvey rat sarcoma viral oncogene homolog |
| HRH4 | 26 | 25 | 29 | 31 | 28 | 31 | Histamine receptor H4 |
| HSP90AA1 | 4 | 4 | 4 | 5 | 4 | 4 | Heat shock protein 90kDa alpha (cytosolic), class A member 1 |
| HSP90AB1 | 4 | 4 | 3 | 4 | 4 | 4 | Heat shock protein 90kDa alpha (cytosolic), class B member 1 |
| HSP90B1 | 4 | 4 | 4 | 4 | 4 | 4 | Heat shock protein 90kDa beta (Grp94), member 1 |
| HSPA1A | 10 | 10 | 10 | 11 | 26 | 12 | Heat shock 70kDa protein 1A |
| HSPBP1 | 0 | 1 | 0 | 0 | 1 | 1 | HSPA (heat shock 70kDa) binding protein, cytoplasmic cochaperone 1 |
| HSPD1 | 4 | 4 | 5 | 6 | 6 | 5 | Heat shock 60kDa protein 1 (chaperonin) |
| Hamp | 0 | 0 | 0 | 0 | 1 | 0 | Hepcidin antimicrobial peptide |
| Havcr2 | 1 | 2 | 2 | 4 | 3 | 2 | Hepatitis A virus cellular receptor 2 |
| Hmox1 | 1 | 2 | 2 | 2 | 2 | 1 | Heme oxygenase (decycling) 1 |
| Hp | 28 | 33 | 35 | 33 | 69 | 52 | Haptoglobin |
| Hrg | 1 | 0 | 2 | 2 | 2 | 2 | Histidine-rich glycoprotein |
| Hrh4 | 20 | 16 | 18 | 23 | 22 | 20 | Histamine receptor H4 |
| Hsf1 | 4 | 4 | 6 | 4 | 6 | 4 | Heat shock factor 1 |
| Hspbp1 | 0 | 2 | 1 | 1 | 2 | 1 | HSPA (heat shock 70kDa) binding protein, cytoplasmic cochaperone 1 |
| Hspd1 | 4 | 3 | 4 | 5 | 5 | 4 | Heat shock protein 1 (chaperonin) |
| ICAM1 | 1 | 1 | 0 | 0 | 3 | 2 | Intercellular adhesion molecule 1 |
| IFI16 | 0 | 0 | 0 | 0 | 4 | 0 | Interferon, gamma-inducible protein 16 |
| IFI27 | 1 | 0 | 1 | 0 | 3 | 2 | Interferon, alpha-inducible protein 27 |
| IFI6 | 0 | 1 | 2 | 1 | 3 | 2 | Interferon, alpha-inducible protein 6 |
| IFIH1 | 5 | 4 | 4 | 5 | 5 | 6 | Interferon induced with helicase C domain 1 |
| IFIT1 | 2 | 2 | 2 | 2 | 6 | 3 | Interferon-induced protein with tetratricopeptide repeats 1 |
| IFIT2 | 1 | 1 | 1 | 1 | 5 | 2 | Interferon-induced protein with tetratricopeptide repeats 2 |
| IFIT3 | 1 | 1 | 1 | 1 | 5 | 0 | Interferon-induced protein with tetratricopeptide repeats 3 |
| IFITM1 | 1 | 1 | 0 | 0 | 4 | 0 | Interferon-induced transmembrane protein 1 (9-27) |
| IFITM2 | 1 | 0 | 0 | 0 | 3 | 0 | Interferon-induced transmembrane protein 2 (1-8D) |
| IFITM3 | 1 | 0 | 0 | 0 | 4 | 1 | Interferon-induced transmembrane protein 3 |
| IFNA1 | 3 | 1 | 3 | 2 | 17 | 6 | Interferon, alpha 1 |
| IFNA10 | 2 | 1 | 1 | 2 | 17 | 6 | Interferon, alpha 10 |
| IFNA14 | 3 | 1 | 4 | 2 | 17 | 6 | Interferon, alpha 14 |
| IFNA16 | 3 | 1 | 1 | 1 | 17 | 6 | Interferon, alpha 16 |
| IFNA17 | 2 | 1 | 3 | 2 | 17 | 6 | Interferon, alpha 17 |
| IFNA2 | 3 | 1 | 4 | 2 | 17 | 6 | Interferon, alpha 2 |
| IFNA21 | 3 | 1 | 2 | 1 | 17 | 6 | Interferon, alpha 21 |
| IFNA4 | 2 | 1 | 1 | 2 | 17 | 6 | Interferon, alpha 4 |
| IFNA5 | 3 | 1 | 4 | 1 | 17 | 6 | Interferon, alpha 5 |
| IFNA6 | 3 | 1 | 2 | 2 | 17 | 6 | Interferon, alpha 6 |
| IFNA7 | 3 | 1 | 4 | 1 | 17 | 6 | Interferon, alpha 7 |
| IFNA8 | 2 | 1 | 1 | 1 | 17 | 6 | Interferon, alpha 8 |
| IFNAR1 | 5 | 6 | 6 | 6 | 3 | 5 | Interferon (alpha, beta and omega) receptor 1 |
| IFNAR2 | 1 | 1 | 1 | 1 | 2 | 0 | Interferon (alpha, beta and omega) receptor 2 |
| IFNB1 | 3 | 1 | 4 | 1 | 17 | 6 | Interferon, beta 1, fibroblast |
| IFNG | 1 | 1 | 1 | 1 | 1 | 0 | Interferon, gamma |
| IFNGR1 | 2 | 2 | 2 | 2 | 2 | 2 | Interferon gamma receptor 1 |
| IFNGR2 | 0 | 0 | 0 | 0 | 1 | 0 | Interferon gamma receptor 2 (interferon gamma transducer 1) |
| IGF1 | 3 | 3 | 3 | 2 | 2 | 2 | Insulin-like growth factor 1 (somatomedin C) |
| IGKC | 3 | 2 | 2 | 1 | 13 | 4 | Immunoglobulin kappa constant |
| IGKV3-11 | 4 | 6 | 9 | 2 | 104 | 32 | Immunoglobulin kappa variable 3-11 |
| IGKV3-20 | 4 | 6 | 9 | 2 | 106 | 32 | Immunoglobulin kappa variable 3-20 |
| IGKV4-1 | 4 | 5 | 9 | 2 | 93 | 29 | Immunoglobulin kappa variable 4-1 |
| IGKV5-2 | 3 | 3 | 8 | 1 | 84 | 26 | Immunoglobulin kappa variable 5-2 |
| IGLL5 | 0 | 0 | 0 | 0 | 2 | 0 | Immunoglobulin lambda-like polypeptide 5 |
| IKBKB | 2 | 2 | 2 | 3 | 2 | 2 | Inhibitor of kappa light polypeptide gene enhancer in B-cells, kinase beta |
| IKBKE | 34 | 38 | 39 | 44 | 54 | 41 | Inhibitor of kappa light polypeptide gene enhancer in B-cells, kinase epsilon |
| IL12A | 1 | 1 | 1 | 0 | 1 | 0 | Interleukin 12A (natural killer cell stimulatory factor 1, cytotoxic lymphocyte maturation factor 1, p35) |
| IL12B | 3 | 1 | 1 | 3 | 5 | 2 | Interleukin 12B (natural killer cell stimulatory factor 2, cytotoxic lymphocyte maturation factor 2, p40) |
| IL13 | 0 | 0 | 0 | 0 | 1 | 0 | Interleukin 13 |
| IL13RA1 | 0 | 1 | 1 | 1 | 1 | 0 | Interleukin 13 receptor, alpha 1 |
| IL15 | 1 | 1 | 1 | 0 | 1 | 0 | Interleukin 15 |
| IL17A | 2 | 3 | 2 | 5 | 4 | 2 | Interleukin 17A |
| IL17C | 4 | 5 | 4 | 6 | 6 | 3 | Interleukin 17C |
| IL17RE | 0 | 1 | 1 | 1 | 3 | 2 | Interleukin 17 receptor E |
| IL18R1 | 0 | 1 | 1 | 0 | 1 | 1 | Interleukin 18 receptor 1 |
| IL1R1 | 12 | 12 | 11 | 12 | 13 | 11 | Interleukin 1 receptor, type I |
| IL1RAP | 9 | 11 | 13 | 18 | 12 | 19 | Interleukin 1 receptor accessory protein |
| IL1RAPL1 | 8 | 13 | 12 | 15 | 11 | 14 | Interleukin 1 receptor accessory protein-like 1 |
| IL1RAPL2 | 6 | 10 | 10 | 11 | 10 | 8 | Interleukin 1 receptor accessory protein-like 2 |
| IL1RL1 | 4 | 8 | 6 | 8 | 8 | 6 | Interleukin 1 receptor-like 1 |
| IL1RL2 | 8 | 10 | 10 | 12 | 10 | 10 | Interleukin 1 receptor-like 2 |
| IL21 | 1 | 1 | 1 | 1 | 1 | 0 | Interleukin 21 |
| IL22 | 1 | 1 | 1 | 1 | 1 | 1 | Interleukin 22 |
| IL23A | 0 | 0 | 0 | 0 | 1 | 0 | Interleukin 23, alpha subunit p19 |
| IL25 | 1 | 2 | 1 | 2 | 2 | 1 | Interleukin 25 |
| IL27 | 0 | 0 | 0 | 0 | 1 | 0 | Interleukin 27 |
| IL28A | 1 | 1 | 1 | 0 | 3 | 0 | Interleukin 28A (interferon, lambda 2) |
| IL29 | 1 | 1 | 1 | 0 | 3 | 0 | Interleukin 29 (interferon, lambda 1) |
| IL2RB | 0 | 0 | 0 | 0 | 1 | 0 | Interleukin 2 receptor, beta |
| IL2RG | 0 | 1 | 1 | 1 | 1 | 0 | Interleukin 2 receptor, gamma |
| IL31 | 0 | 0 | 0 | 0 | 1 | 0 | Interleukin 31 |
| IL33 | 0 | 0 | 0 | 0 | 1 | 0 | Interleukin 33 |
| IL37 | 0 | 1 | 1 | 1 | 7 | 3 | Interleukin 37 |
| IL3RA | 0 | 4 | 4 | 5 | 3 | 0 | Interleukin 3 receptor, alpha (low affinity) |
| IL4R | 2 | 1 | 1 | 1 | 2 | 1 | Interleukin 4 receptor |
| IL6 | 0 | 1 | 1 | 1 | 1 | 0 | Interleukin 6 (interferon, beta 2) |
| IL6ST | 7 | 8 | 12 | 9 | 15 | 12 | Interleukin 6 signal transducer (gp130, oncostatin M receptor) |
| IL9 | 0 | 0 | 1 | 0 | 1 | 0 | Interleukin 9 |
| ILF3 | 7 | 7 | 8 | 6 | 9 | 5 | Interleukin enhancer binding factor 3, 90kDa |
| IMPDH2 | 1 | 2 | 2 | 2 | 4 | 4 | IMP (inosine 5'-monophosphate) dehydrogenase 2 |
| INPP5D | 9 | 9 | 9 | 11 | 9 | 11 | Inositol polyphosphate-5-phosphatase, 145kDa |
| IP6K1 | 4 | 4 | 4 | 4 | 4 | 4 | Inositol hexakisphosphate kinase 1 |
| IRAK1 | 27 | 39 | 44 | 38 | 62 | 40 | Interleukin-1 receptor-associated kinase 1 |
| IRAK1BP1 | 1 | 1 | 1 | 1 | 1 | 1 | Interleukin-1 receptor-associated kinase 1 binding protein 1 |
| IRAK2 | 27 | 35 | 41 | 43 | 60 | 44 | Interleukin-1 receptor-associated kinase 2 |
| IRAK3 | 31 | 37 | 34 | 40 | 63 | 45 | Interleukin-1 receptor-associated kinase 3 |
| IRAK4 | 27 | 38 | 37 | 46 | 57 | 45 | Interleukin-1 receptor-associated kinase 4 |
| IRF2BP1 | 1 | 1 | 2 | 2 | 3 | 2 | Interferon regulatory factor 2 binding protein 1 |
| IRF3 | 4 | 3 | 4 | 3 | 6 | 3 | Interferon regulatory factor 3 |
| IRF4 | 7 | 8 | 8 | 8 | 9 | 10 | Interferon regulatory factor 4 |
| IRF5 | 5 | 6 | 8 | 7 | 9 | 8 | Interferon regulatory factor 5 |
| IRF6 | 4 | 6 | 5 | 6 | 7 | 6 | Interferon regulatory factor 6 |
| IRF7 | 3 | 5 | 4 | 4 | 5 | 5 | Interferon regulatory factor 7 |
| IRF8 | 6 | 8 | 8 | 8 | 9 | 10 | Interferon regulatory factor 8 |
| IRGM | 0 | 0 | 0 | 0 | 1 | 0 | Immunity-related GTPase family, M |
| ISG15 | 1 | 2 | 3 | 5 | 5 | 4 | ISG15 ubiquitin-like modifier |
| ITCH | 24 | 31 | 33 | 36 | 37 | 30 | Itchy E3 ubiquitin protein ligase homolog (mouse) |
| ITGA3 | 10 | 10 | 10 | 10 | 19 | 18 | Integrin, alpha 3 (antigen CD49C, alpha 3 subunit of VLA-3 receptor) |
| ITGAM | 30 | 27 | 28 | 26 | 37 | 42 | Integrin, alpha M (complement component 3 receptor 3 subunit) |
| ITGAX | 29 | 30 | 27 | 28 | 37 | 42 | Integrin, alpha X (complement component 3 receptor 4 subunit) |
| ITGB1 | 9 | 12 | 11 | 14 | 17 | 12 | Integrin, beta 1 (fibronectin receptor, beta polypeptide, antigen CD29 includes MDF2, MSK12) |
| ITGB2 | 7 | 7 | 7 | 8 | 7 | 8 | Integrin, beta 2 (complement component 3 receptor 3 and 4 subunit) |
| ITPR1 | 5 | 6 | 5 | 7 | 6 | 8 | Inositol 1,4,5-trisphosphate receptor, type 1 |
| ITPR3 | 5 | 7 | 5 | 8 | 6 | 11 | Inositol 1,4,5-trisphosphate receptor, type 3 |
| Ifih1 | 5 | 4 | 4 | 5 | 5 | 6 | Interferon induced with helicase C domain 1 |
| Ifit1 | 2 | 2 | 2 | 2 | 6 | 3 | Interferon-induced protein with tetratricopeptide repeats 1 |
| Ifit2 | 1 | 1 | 1 | 1 | 5 | 2 | Interferon-induced protein with tetratricopeptide repeats 2 |
| Ifit3 | 1 | 1 | 1 | 1 | 5 | 2 | Interferon-induced protein with tetratricopeptide repeats 3 |
| Ifnar1 | 2 | 3 | 3 | 3 | 3 | 0 | Interferon (alpha and beta) receptor 1 |
| Ifnb1 | 1 | 0 | 1 | 1 | 16 | 5 | Interferon beta 1, fibroblast |
| Igf1 | 3 | 3 | 3 | 2 | 2 | 3 | Insulin-like growth factor 1 |
| Iigp1 | 0 | 0 | 0 | 0 | 2 | 7 | Interferon inducible GTPase 1 |
| Ikbkb | 28 | 35 | 43 | 45 | 53 | 46 | Inhibitor of kappaB kinase beta |
| Ikbke | 18 | 20 | 31 | 29 | 41 | 24 | Inhibitor of kappaB kinase epsilon |
| Il10 | 1 | 1 | 2 | 1 | 1 | 1 | Interleukin 10 |
| Il13 | 0 | 0 | 0 | 0 | 1 | 0 | Interleukin 13 |
| Il15 | 1 | 1 | 1 | 0 | 1 | 0 | Interleukin 15 |
| Il17a | 2 | 3 | 2 | 4 | 3 | 2 | Interleukin 17A |
| Il17c | 4 | 5 | 4 | 6 | 6 | 4 | Interleukin 17C |
| Il17re | 0 | 0 | 0 | 0 | 3 | 2 | Interleukin 17 receptor E |
| Il18r1 | 3 | 6 | 7 | 5 | 7 | 5 | Interleukin 18 receptor 1 |
| Il1r1 | 9 | 11 | 10 | 14 | 12 | 12 | Interleukin 1 receptor, type I |
| Il1rapl1 | 9 | 10 | 8 | 12 | 11 | 10 | Interleukin 1 receptor accessory protein-like 1 |
| Il1rapl2 | 12 | 13 | 15 | 17 | 13 | 15 | Interleukin 1 receptor accessory protein-like 2 |
| Il1rl1 | 9 | 12 | 13 | 15 | 12 | 13 | Interleukin 1 receptor-like 1 |
| Il1rl2 | 9 | 11 | 10 | 11 | 11 | 12 | Interleukin 1 receptor-like 2 |
| Il22 | 1 | 1 | 1 | 1 | 1 | 1 | Interleukin 22 |
| Il23a | 0 | 0 | 0 | 0 | 1 | 0 | Interleukin 23, alpha subunit p19 |
| Il23r | 3 | 4 | 5 | 3 | 6 | 4 | Interleukin 23 receptor |
| Il27 | 0 | 0 | 0 | 0 | 1 | 0 | Interleukin 27 |
| Il4 | 0 | 0 | 0 | 0 | 1 | 0 | Interleukin 4 |
| Il4ra | 0 | 0 | 0 | 0 | 1 | 0 | Interleukin 4 receptor, alpha |
| Il6 | 0 | 1 | 1 | 1 | 1 | 0 | Interleukin 6 |
| Il9 | 0 | 0 | 0 | 0 | 1 | 0 | Interleukin 9 |
| Impdh2 | 2 | 2 | 2 | 2 | 4 | 4 | Inosine 5'-phosphate dehydrogenase 2 |
| Ip6k1 | 3 | 3 | 3 | 4 | 3 | 3 | Inositol hexaphosphate kinase 1 |
| Irak1 | 25 | 42 | 43 | 41 | 55 | 44 | Interleukin-1 receptor-associated kinase 1 |
| Irak2 | 1 | 1 | 1 | 1 | 2 | 1 | Interleukin-1 receptor-associated kinase 2 |
| Irak4 | 1 | 1 | 1 | 1 | 1 | 1 | Interleukin-1 receptor-associated kinase 4 |
| Irf1 | 6 | 6 | 8 | 6 | 9 | 8 | Interferon regulatory factor 1 |
| Irf7 | 5 | 7 | 6 | 8 | 9 | 8 | Interferon regulatory factor 7 |
| Irgm1 | 0 | 0 | 0 | 0 | 2 | 7 | Immunity-related GTPase family M member 1 |
| Isg15 | 0 | 2 | 3 | 4 | 4 | 3 | ISG15 ubiquitin-like modifier |
| Itch | 24 | 31 | 33 | 36 | 37 | 30 | Itchy, E3 ubiquitin protein ligase |
| Itgam | 29 | 29 | 28 | 27 | 38 | 41 | Integrin alpha M |
| Itgax | 30 | 29 | 28 | 27 | 38 | 43 | Integrin alpha X |
| Itpr1 | 5 | 5 | 5 | 7 | 6 | 6 | Inositol 1,4,5-trisphosphate receptor 1 |
| Itpr3 | 5 | 7 | 5 | 8 | 6 | 11 | Inositol 1,4,5-triphosphate receptor 3 |
| JAK2 | 30 | 47 | 43 | 38 | 54 | 38 | Janus kinase 2 |
| JAK3 | 2 | 5 | 2 | 3 | 4 | 3 | Janus kinase 3 |
| JAM3 | 7 | 13 | 12 | 10 | 11 | 11 | Junctional adhesion molecule 3 |
| JUN | 3 | 4 | 4 | 5 | 6 | 5 | Jun proto-oncogene |
| Jak2 | 31 | 45 | 43 | 40 | 52 | 39 | Janus kinase 2 |
| Jak3 | 34 | 43 | 42 | 41 | 49 | 41 | Janus kinase 3 |
| Jam3 | 22 | 23 | 20 | 30 | 35 | 28 | Junction adhesion molecule 3 |
| KCNJ8 | 13 | 15 | 14 | 14 | 15 | 17 | Potassium inwardly-rectifying channel, subfamily J, member 8 |
| KDM1A | 5 | 5 | 6 | 5 | 7 | 6 | Lysine (K)-specific demethylase 1A |
| KHSRP | 9 | 10 | 11 | 7 | 12 | 13 | KH-type splicing regulatory protein |
| KLK1 | 30 | 34 | 37 | 35 | 69 | 45 | Kallikrein 1 |
| KLRG1 | 3 | 3 | 12 | 0 | 17 | 13 | Killer cell lectin-like receptor subfamily G, member 1 |
| KPNA1 | 7 | 7 | 7 | 8 | 7 | 7 | Karyopherin alpha 1 (importin alpha 5) |
| Kcnj8 | 13 | 15 | 14 | 14 | 15 | 17 | Potassium inwardly-rectifying channel, subfamily J, member 8 |
| Khsrp | 9 | 11 | 11 | 8 | 13 | 12 | KH-type splicing regulatory protein |
| Kitl | 1 | 1 | 1 | 1 | 1 | 2 | Kit ligand |
| Klrg1 | 3 | 6 | 11 | 1 | 27 | 12 | Killer cell lectin-like receptor subfamily G, member 1 |
| LAIR1 | 0 | 0 | 3 | 0 | 23 | 1 | Leukocyte-associated immunoglobulin-like receptor 1 |
| LAT | 0 | 0 | 0 | 0 | 1 | 0 | Linker for activation of T cells |
| LBP | 6 | 5 | 7 | 5 | 8 | 5 | Lipopolysaccharide binding protein |
| LCN2 | 2 | 4 | 2 | 1 | 5 | 1 | Lipocalin 2 |
| LGALS2 | 0 | 0 | 0 | 0 | 1 | 0 | Lectin, galactoside-binding, soluble, 2 |
| LGALS4 | 7 | 8 | 8 | 6 | 16 | 11 | Lectin, galactoside-binding, soluble, 4 |
| LGALS8 | 8 | 8 | 8 | 7 | 12 | 11 | Lectin, galactoside-binding, soluble, 8 |
| LGALS9 | 4 | 3 | 3 | 3 | 12 | 8 | Lectin, galactoside-binding, soluble, 9 |
| LGMN | 2 | 2 | 2 | 3 | 2 | 1 | Legumain |
| LILRA5 | 0 | 0 | 70 | 1 | 30 | 1 | Leukocyte immunoglobulin-like receptor, subfamily A (with TM domain), member 5 |
| LILRB3 | 0 | 0 | 74 | 1 | 31 | 3 | Leukocyte immunoglobulin-like receptor, subfamily B (with TM and ITIM domains), member 3 |
| LOC100287178 | 28 | 30 | 31 | 39 | 60 | 37 | Ubiquitin carboxyl-terminal hydrolase 17 |
| LPCAT2 | 17 | 16 | 18 | 18 | 19 | 16 | Lysophosphatidylcholine acyltransferase 2 |
| LRRFIP1 | 2 | 2 | 2 | 3 | 2 | 2 | Leucine rich repeat (in FLII) interacting protein 1 |
| LRRFIP2 | 2 | 2 | 2 | 3 | 2 | 2 | Leucine rich repeat (in FLII) interacting protein 2 |
| LTB4R | 40 | 35 | 44 | 40 | 44 | 47 | Leukotriene B4 receptor |
| LTBR | 4 | 3 | 3 | 2 | 4 | 2 | Lymphotoxin beta receptor (TNFR superfamily, member 3) |
| LY86 | 1 | 2 | 2 | 1 | 1 | 0 | Lymphocyte antigen 86 |
| LY9 | 0 | 0 | 0 | 0 | 6 | 0 | Lymphocyte antigen 9 |
| LY96 | 0 | 1 | 1 | 0 | 1 | 1 | Lymphocyte antigen 96 |
| Lair1 | 0 | 0 | 0 | 0 | 1 | 0 | Leukocyte-associated Ig-like receptor 1 |
| Lbp | 3 | 3 | 5 | 3 | 4 | 3 | Lipopolysaccharide binding protein |
| Lgals9 | 7 | 8 | 8 | 7 | 16 | 11 | Lectin, galactose binding, soluble 9 |
| Lilrb3 | 0 | 0 | 80 | 0 | 30 | 3 | Leukocyte immunoglobulin-like receptor, subfamily B (with TM and ITIM domains), member 3 |
| Lrrfip2 | 2 | 2 | 2 | 3 | 2 | 2 | Leucine rich repeat (in FLII) interacting protein 2 |
| Ly86 | 1 | 2 | 2 | 1 | 1 | 0 | Lymphocyte antigen 86 |
| Ly96 | 0 | 1 | 1 | 1 | 1 | 1 | Lymphocyte antigen 96 |
| MAFB | 4 | 5 | 4 | 6 | 7 | 6 | v-maf musculoaponeurotic fibrosarcoma oncogene homolog B (avian) |
| MALT1 | 11 | 16 | 18 | 16 | 23 | 22 | Mucosa associated lymphoid tissue lymphoma translocation gene 1 |
| MAP2K1 | 30 | 36 | 45 | 44 | 51 | 44 | Mitogen-activated protein kinase kinase 1 |
| MAP2K3 | 29 | 39 | 39 | 56 | 48 | 39 | Mitogen-activated protein kinase kinase 3 |
| MAP2K4 | 30 | 39 | 37 | 59 | 44 | 41 | Mitogen-activated protein kinase kinase 4 |
| MAP2K6 | 27 | 40 | 41 | 56 | 48 | 38 | Mitogen-activated protein kinase kinase 6 |
| MAP2K7 | 30 | 38 | 39 | 50 | 50 | 43 | Mitogen-activated protein kinase kinase 7 |
| MAP3K14 | 33 | 33 | 37 | 56 | 42 | 49 | Mitogen-activated protein kinase kinase kinase 14 |
| MAP3K3 | 26 | 43 | 40 | 49 | 46 | 46 | Mitogen-activated protein kinase kinase kinase 3 |
| MAP3K4 | 1 | 1 | 1 | 1 | 1 | 1 | Mitogen-activated protein kinase kinase kinase 4 |
| MAP3K5 | 29 | 36 | 42 | 52 | 49 | 42 | Mitogen-activated protein kinase kinase kinase 5 |
| MAP3K7 | 39 | 43 | 41 | 42 | 49 | 36 | Mitogen-activated protein kinase kinase kinase 7 |
| MAP3K8 | 26 | 38 | 38 | 67 | 43 | 38 | Mitogen-activated protein kinase kinase kinase 8 |
| MAPK1 | 34 | 38 | 39 | 37 | 54 | 48 | Mitogen-activated protein kinase 1 |
| MAPK10 | 32 | 40 | 40 | 39 | 53 | 46 | Mitogen-activated protein kinase 10 |
| MAPK11 | 35 | 38 | 40 | 39 | 51 | 47 | Mitogen-activated protein kinase 11 |
| MAPK3 | 33 | 39 | 39 | 40 | 53 | 46 | Mitogen-activated protein kinase 3 |
| MAPK7 | 35 | 37 | 38 | 39 | 52 | 49 | Mitogen-activated protein kinase 7 |
| MAPK8 | 28 | 35 | 40 | 48 | 54 | 45 | Mitogen-activated protein kinase 8 |
| MAPK9 | 3 | 3 | 3 | 3 | 3 | 3 | Mitogen-activated protein kinase 9 |
| MAPKAPK3 | 16 | 16 | 18 | 20 | 19 | 19 | Mitogen-activated protein kinase-activated protein kinase 3 |
| MARCO | 30 | 42 | 46 | 34 | 46 | 52 | Macrophage receptor with collagenous structure |
| MASP1 | 28 | 39 | 38 | 38 | 56 | 51 | Mannan-binding lectin serine peptidase 1 (C4/C2 activating component of Ra-reactive factor) |
| MAVS | 0 | 0 | 0 | 0 | 1 | 0 | Mitochondrial antiviral signaling protein |
| MEF2A | 4 | 4 | 4 | 3 | 5 | 3 | Myocyte enhancer factor 2A |
| MEF2C | 4 | 4 | 4 | 3 | 5 | 3 | Myocyte enhancer factor 2C |
| MEFV | 9 | 7 | 6 | 2 | 72 | 41 | Mediterranean fever |
| MERTK | 32 | 42 | 45 | 41 | 50 | 40 | c-mer proto-oncogene tyrosine kinase |
| MFN1 | 2 | 2 | 2 | 2 | 2 | 2 | Mitofusin 1 |
| MFN2 | 2 | 2 | 2 | 3 | 2 | 2 | Mitofusin 2 |
| MKNK1 | 7 | 6 | 11 | 12 | 13 | 9 | MAP kinase interacting serine/threonine kinase 1 |
| MLST8 | 18 | 18 | 19 | 17 | 22 | 18 | MTOR associated protein, LST8 homolog (S. cerevisiae) |
| MMP9 | 11 | 13 | 13 | 12 | 20 | 18 | Matrix metallopeptidase 9 (gelatinase B, 92kDa gelatinase, 92kDa type IV collagenase) |
| MRGPRX2 | 19 | 17 | 22 | 16 | 41 | 36 | MAS-related GPR, member X2 |
| MSR1 | 20 | 26 | 22 | 20 | 28 | 29 | Macrophage scavenger receptor 1 |
| MTA1 | 1 | 2 | 2 | 2 | 3 | 3 | Metastasis associated 1 |
| MTOR | 1 | 1 | 1 | 1 | 1 | 1 | Mechanistic target of rapamycin (serine/threonine kinase) |
| MUC1 | 0 | 0 | 0 | 0 | 1 | 0 | Mucin 1, cell surface associated |
| MX1 | 6 | 5 | 5 | 6 | 7 | 7 | Myxovirus (influenza virus) resistance 1, interferon-inducible protein p78 (mouse) |
| MX2 | 3 | 2 | 4 | 4 | 6 | 5 | Myxovirus (influenza virus) resistance 2 (mouse) |
| MYD88 | 0 | 1 | 1 | 1 | 1 | 1 | Myeloid differentiation primary response gene (88) |
| Malt1 | 10 | 13 | 13 | 17 | 13 | 19 | Mucosa associated lymphoid tissue lymphoma translocation gene 1 |
| Mapkapk2 | 32 | 39 | 39 | 44 | 54 | 42 | MAP kinase-activated protein kinase 2 |
| 5-Mar | 1 | 1 | 1 | 2 | 1 | 2 | Membrane-associated ring finger (C3HC4) 5 |
| Marco | 29 | 41 | 47 | 36 | 45 | 52 | Macrophage receptor with collagenous structure |
| Masp1 | 30 | 34 | 39 | 37 | 61 | 49 | Mannan-binding lectin serine peptidase 1 |
| Masp2 | 13 | 15 | 16 | 18 | 19 | 14 | Mannan-binding lectin serine peptidase 2 |
| Mbl1 | 13 | 10 | 10 | 13 | 23 | 16 | Mannose-binding lectin (protein A) 1 |
| Mbl2 | 24 | 29 | 26 | 22 | 33 | 25 | Mannose-binding lectin (protein C) 2 |
| Mfn1 | 2 | 2 | 2 | 3 | 2 | 2 | Mitofusin 1 |
| Mfn2 | 2 | 2 | 2 | 2 | 2 | 1 | Mitofusin 2 |
| Mif | 1 | 1 | 2 | 1 | 3 | 1 | Macrophage migration inhibitory factor |
| Mknk1 | 31 | 37 | 41 | 42 | 56 | 43 | MAP kinase-interacting serine/threonine kinase 1 |
| Msr1 | 20 | 26 | 24 | 20 | 27 | 25 | Macrophage scavenger receptor 1 |
| Mta1 | 4 | 5 | 7 | 8 | 9 | 7 | Metastasis associated 1 |
| Mx1 | 6 | 5 | 5 | 6 | 7 | 7 | Myxovirus (influenza virus) resistance 1 |
| Mx2 | 6 | 5 | 5 | 6 | 7 | 7 | Myxovirus (influenza virus) resistance 2 |
| Myd88 | 4 | 3 | 6 | 5 | 4 | 8 | Myeloid differentiation primary response gene 88 |
| NCF1 | 2 | 2 | 1 | 2 | 3 | 1 | Neutrophil cytosolic factor 1 |
| NCF2 | 12 | 12 | 13 | 15 | 16 | 12 | Neutrophil cytosolic factor 2 |
| NDUFA13 | 0 | 1 | 0 | 0 | 2 | 0 | NADH dehydrogenase (ubiquinone) 1 alpha subcomplex, 13 |
| NEU1 | 0 | 0 | 0 | 0 | 8 | 0 | Sialidase 1 (lysosomal sialidase) |
| NFAT5 | 5 | 4 | 4 | 5 | 5 | 6 | Nuclear factor of activated T-cells 5, tonicity-responsive |
| NFATC2 | 4 | 4 | 4 | 5 | 5 | 5 | Nuclear factor of activated T-cells, cytoplasmic, calcineurin-dependent 2 |
| NFATC3 | 4 | 4 | 4 | 5 | 5 | 5 | Nuclear factor of activated T-cells, cytoplasmic, calcineurin-dependent 3 |
| NFIL3 | 4 | 2 | 3 | 3 | 4 | 4 | Nuclear factor, interleukin 3 regulated |
| NFKB2 | 37 | 38 | 39 | 35 | 58 | 43 | Nuclear factor of kappa light polypeptide gene enhancer in B-cells 2 (p49/p100) |
| NFKBIA | 0 | 0 | 1 | 1 | 1 | 1 | Nuclear factor of kappa light polypeptide gene enhancer in B-cells inhibitor, alpha |
| NFKBIB | 25 | 24 | 23 | 22 | 32 | 29 | Nuclear factor of kappa light polypeptide gene enhancer in B-cells inhibitor, beta |
| NFKBIE | 8 | 10 | 8 | 10 | 13 | 11 | Nuclear factor of kappa light polypeptide gene enhancer in B-cells inhibitor, epsilon |
| NKIRAS1 | 30 | 30 | 40 | 42 | 61 | 47 | NFKB inhibitor interacting Ras-like 1 |
| NKIRAS2 | 2 | 1 | 2 | 2 | 2 | 2 | NFKB inhibitor interacting Ras-like 2 |
| NLRC4 | 0 | 0 | 0 | 0 | 2 | 4 | NLR family, CARD domain containing 4 |
| NLRC5 | 3 | 2 | 2 | 3 | 2 | 1 | NLR family, CARD domain containing 5 |
| NLRP1 | 6 | 9 | 9 | 6 | 20 | 9 | NLR family, pyrin domain containing 1 |
| NLRP10 | 0 | 0 | 0 | 0 | 1 | 0 | NLR family, pyrin domain containing 10 |
| NLRP11 | 8 | 9 | 7 | 5 | 20 | 8 | NLR family, pyrin domain containing 11 |
| NLRP12 | 6 | 8 | 5 | 5 | 19 | 7 | NLR family, pyrin domain containing 12 |
| NLRP13 | 11 | 12 | 12 | 11 | 27 | 17 | NLR family, pyrin domain containing 13 |
| NLRP14 | 12 | 11 | 10 | 11 | 26 | 16 | NLR family, pyrin domain containing 14 |
| NLRP2 | 9 | 9 | 8 | 9 | 27 | 16 | NLR family, pyrin domain containing 2 |
| NLRP3 | 8 | 9 | 10 | 7 | 26 | 12 | NLR family, pyrin domain containing 3 |
| NLRP4 | 9 | 9 | 9 | 9 | 26 | 15 | NLR family, pyrin domain containing 4 |
| NLRP5 | 11 | 11 | 11 | 11 | 29 | 14 | NLR family, pyrin domain containing 5 |
| NLRP7 | 3 | 3 | 3 | 3 | 17 | 5 | NLR family, pyrin domain containing 7 |
| NLRP8 | 9 | 9 | 8 | 7 | 22 | 11 | NLR family, pyrin domain containing 8 |
| NLRP9 | 9 | 8 | 9 | 8 | 22 | 12 | NLR family, pyrin domain containing 9 |
| NOD1 | 1 | 1 | 1 | 1 | 1 | 1 | Nucleotide-binding oligomerization domain containing 1 |
| NOD2 | 8 | 10 | 9 | 8 | 21 | 12 | Nucleotide-binding oligomerization domain containing 2 |
| NOS2 | 3 | 4 | 3 | 2 | 4 | 3 | Nitric oxide synthase 2, inducible |
| NOX1 | 6 | 6 | 6 | 6 | 7 | 5 | NADPH oxidase 1 |
| NOX4 | 5 | 6 | 6 | 6 | 7 | 5 | NADPH oxidase 4 |
| NOXA1 | 2 | 2 | 2 | 4 | 3 | 1 | NADPH oxidase activator 1 |
| NR3C1 | 30 | 31 | 34 | 44 | 51 | 42 | Nuclear receptor subfamily 3, group C, member 1 (glucocorticoid receptor) |
| NR4A3 | 30 | 30 | 34 | 43 | 51 | 40 | Nuclear receptor subfamily 4, group A, member 3 |
| NRAS | 34 | 33 | 44 | 38 | 51 | 50 | Neuroblastoma RAS viral (v-ras) oncogene homolog |
| NUMBL | 8 | 8 | 7 | 9 | 8 | 6 | Numb homolog (Drosophila)-like |
| NUP153 | 2 | 3 | 4 | 3 | 2 | 2 | Nucleoporin 153kDa |
| NUP214 | 1 | 1 | 0 | 1 | 1 | 0 | Nucleoporin 214kDa |
| NXN | 1 | 1 | 1 | 2 | 1 | 1 | Nucleoredoxin |
| Naip2 | 4 | 5 | 6 | 6 | 8 | 6 | NLR family, apoptosis inhibitory protein 2 |
| Naip5 | 4 | 5 | 6 | 7 | 7 | 5 | NLR family, apoptosis inhibitory protein 5 |
| Nfat5 | 5 | 4 | 4 | 5 | 5 | 5 | Nuclear factor of activated T-cells 5 |
| Nfatc2 | 4 | 4 | 4 | 4 | 5 | 5 | Nuclear factor of activated T-cells, cytoplasmic, calcineurin-dependent 2 |
| Nfatc3 | 5 | 4 | 5 | 6 | 6 | 5 | Nuclear factor of activated T-cells, cytoplasmic, calcineurin-dependent 3 |
| Nfatc4 | 4 | 4 | 4 | 5 | 5 | 5 | Nuclear factor of activated T-cells, cytoplasmic, calcineurin-dependent 4 |
| Nfil3 | 4 | 2 | 3 | 3 | 4 | 4 | Nuclear factor, interleukin 3, regulated |
| Nfkb1 | 36 | 39 | 38 | 37 | 56 | 44 | Nuclear factor of kappa light polypeptide gene enhancer in B-cells 1, p105 |
| Nfkbia | 38 | 38 | 42 | 38 | 51 | 43 | Nuclear factor of kappa light polypeptide gene enhancer in B-cells inhibitor, alpha |
| Nlrc4 | 1 | 0 | 0 | 1 | 5 | 3 | NLR family, CARD domain containing 4 |
| Nlrp3 | 7 | 8 | 9 | 10 | 25 | 14 | NLR family, pyrin domain containing 3 |
| Nlrx1 | 5 | 6 | 6 | 6 | 20 | 9 | NLR family member X1 |
| Nod1 | 15 | 13 | 15 | 15 | 29 | 20 | Nucleotide-binding oligomerization domain containing 1 |
| Nod2 | 12 | 13 | 12 | 10 | 30 | 18 | Nucleotide-binding oligomerization domain containing 2 |
| Nos2 | 5 | 7 | 5 | 5 | 6 | 6 | Nitric oxide synthase 2, inducible |
| Notch1 | 36 | 37 | 36 | 40 | 59 | 42 | Notch gene homolog 1 (Drosophila) |
| Nox1 | 6 | 6 | 6 | 6 | 7 | 5 | NADPH oxidase 1 |
| Nr3c1 | 29 | 31 | 34 | 43 | 51 | 42 | Nuclear receptor subfamily 3, group C, member 1 |
| Nras | 34 | 33 | 44 | 38 | 51 | 50 | Neuroblastoma ras oncogene |
| OAS1 | 1 | 0 | 1 | 1 | 4 | 1 | 2'-5'-oligoadenylate synthetase 1, 40/46kDa |
| OAS3 | 1 | 0 | 1 | 1 | 4 | 1 | 2'-5'-oligoadenylate synthetase 3, 100kDa |
| OLFM4 | 12 | 9 | 11 | 10 | 13 | 12 | Olfactomedin 4 |
| ORAI1 | 2 | 2 | 2 | 2 | 3 | 2 | ORAI calcium release-activated calcium modulator 1 |
| OTUB1 | 0 | 0 | 0 | 0 | 2 | 1 | OTU domain, ubiquitin aldehyde binding 1 |
| OTUB2 | 0 | 0 | 0 | 0 | 2 | 1 | OTU domain, ubiquitin aldehyde binding 2 |
| OTUD5 | 1 | 0 | 1 | 4 | 5 | 3 | OTU domain containing 5 |
| OTUD7B | 4 | 4 | 4 | 4 | 4 | 4 | OTU domain containing 7B |
| Oas1a | 1 | 0 | 1 | 1 | 4 | 1 | 2'-5' oligoadenylate synthetase 1A |
| Oas1b | 0 | 0 | 1 | 1 | 4 | 1 | 2'-5' oligoadenylate synthetase 1B |
| Oas1c | 1 | 0 | 1 | 1 | 4 | 1 | 2'-5' oligoadenylate synthetase 1C |
| Oas1d | 1 | 0 | 1 | 1 | 4 | 1 | 2'-5' oligoadenylate synthetase 1D |
| Oas1e | 1 | 0 | 1 | 1 | 4 | 1 | 2'-5' oligoadenylate synthetase 1E |
| Oas1g | 1 | 0 | 1 | 1 | 4 | 1 | 2'-5' oligoadenylate synthetase 1G |
| Oas1h | 1 | 0 | 1 | 1 | 4 | 1 | 2'-5' oligoadenylate synthetase 1H |
| Oas2 | 1 | 0 | 1 | 1 | 4 | 1 | 2'-5' oligoadenylate synthetase 2 |
| Orai1 | 2 | 2 | 2 | 2 | 3 | 2 | ORAI calcium release-activated calcium modulator 1 |
| P2RX7 | 4 | 7 | 6 | 5 | 6 | 5 | Purinergic receptor P2X, ligand-gated ion channel, 7 |
| P2rx7 | 5 | 7 | 6 | 8 | 7 | 5 | Purinergic receptor P2X, ligand-gated ion channel, 7 |
| PALM3 | 0 | 2 | 2 | 1 | 5 | 0 | Paralemmin 3 |
| PANX1 | 3 | 3 | 3 | 4 | 3 | 3 | Pannexin 1 |
| PARK2 | 0 | 2 | 4 | 4 | 5 | 3 | Parkinson protein 2, E3 ubiquitin protein ligase (parkin) |
| PCBP1 | 11 | 12 | 12 | 10 | 14 | 13 | Poly(rC) binding protein 1 |
| PCBP2 | 10 | 11 | 11 | 10 | 14 | 13 | Poly(rC) binding protein 2 |
| PDCD1 | 0 | 1 | 1 | 0 | 1 | 0 | Programmed cell death 1 |
| PDCD1LG2 | 1 | 2 | 2 | 1 | 2 | 0 | Programmed cell death 1 ligand 2 |
| PELI3 | 2 | 2 | 2 | 4 | 3 | 3 | Pellino homolog 3 (Drosophila) |
| PGLYRP1 | 1 | 1 | 0 | 0 | 4 | 2 | Peptidoglycan recognition protein 1 |
| PGLYRP2 | 1 | 2 | 0 | 0 | 4 | 2 | Peptidoglycan recognition protein 2 |
| PGLYRP3 | 1 | 1 | 0 | 0 | 4 | 2 | Peptidoglycan recognition protein 3 |
| PIAS1 | 4 | 4 | 4 | 5 | 6 | 6 | Protein inhibitor of activated STAT, 1 |
| PIAS2 | 4 | 4 | 4 | 5 | 6 | 6 | Protein inhibitor of activated STAT, 2 |
| PIAS4 | 4 | 5 | 4 | 5 | 6 | 6 | Protein inhibitor of activated STAT, 4 |
| PIK3C3 | 13 | 13 | 13 | 17 | 13 | 13 | Phosphoinositide-3-kinase, class 3 |
| PIK3CB | 13 | 14 | 14 | 17 | 14 | 13 | Phosphoinositide-3-kinase, catalytic, beta polypeptide |
| PIK3R1 | 31 | 34 | 30 | 29 | 41 | 36 | Phosphoinositide-3-kinase, regulatory subunit 1 (alpha) |
| PIN1 | 1 | 0 | 0 | 0 | 1 | 0 | Peptidylprolyl cis/trans isomerase, NIMA-interacting 1 |
| PKLR | 1 | 1 | 1 | 1 | 2 | 1 | Pyruvate kinase, liver and RBC |
| PKN1 | 35 | 41 | 39 | 42 | 50 | 43 | Protein kinase N1 |
| PLA2G4A | 0 | 1 | 1 | 1 | 1 | 1 | Phospholipase A2, group IVA (cytosolic, calcium-dependent) |
| PLAUR | 0 | 0 | 0 | 0 | 1 | 2 | Plasminogen activator, urokinase receptor |
| PLCG2 | 40 | 38 | 41 | 45 | 44 | 42 | Phospholipase C, gamma 2 (phosphatidylinositol-specific) |
| PLG | 24 | 35 | 37 | 37 | 67 | 50 | Plasminogen |
| PLK1 | 32 | 38 | 40 | 41 | 59 | 40 | Polo-like kinase 1 |
| PLTP | 6 | 4 | 7 | 4 | 5 | 4 | Phospholipid transfer protein |
| PLXNA4 | 12 | 16 | 16 | 13 | 18 | 20 | Plexin A4 |
| PMAIP1 | 0 | 0 | 0 | 0 | 1 | 0 | Phorbol-12-myristate-13-acetate-induced protein 1 |
| PML | 1 | 1 | 1 | 0 | 1 | 0 | Promyelocytic leukemia |
| POLR3A | 3 | 3 | 3 | 4 | 3 | 3 | Polymerase (RNA) III (DNA directed) polypeptide A, 155kDa |
| POLR3E | 1 | 1 | 1 | 1 | 1 | 1 | Polymerase (RNA) III (DNA directed) polypeptide E (80kD) |
| POLR3H | 1 | 1 | 1 | 1 | 1 | 1 | Polymerase (RNA) III (DNA directed) polypeptide H (22.9kD) |
| PPARG | 30 | 31 | 34 | 43 | 51 | 40 | Peroxisome proliferator-activated receptor gamma |
| PPIA | 12 | 12 | 16 | 17 | 22 | 14 | Peptidylprolyl isomerase A (cyclophilin A) |
| PPP1CA | 10 | 11 | 9 | 16 | 13 | 13 | Protein phosphatase 1, catalytic subunit, alpha isozyme |
| PPP3CA | 10 | 11 | 9 | 16 | 13 | 13 | Protein phosphatase 3, catalytic subunit, alpha isozyme |
| PPP3R1 | 33 | 32 | 38 | 36 | 45 | 35 | Protein phosphatase 3, regulatory subunit B, alpha |
| PPP4C | 10 | 11 | 9 | 16 | 13 | 13 | Protein phosphatase 4, catalytic subunit |
| PRKCA | 37 | 39 | 40 | 39 | 53 | 42 | Protein kinase C, alpha |
| PRKCD | 14 | 14 | 15 | 15 | 18 | 16 | Protein kinase C, delta |
| PRKCSH | 1 | 1 | 1 | 1 | 2 | 2 | Protein kinase C substrate 80K-H |
| PRMT1 | 4 | 5 | 4 | 6 | 8 | 7 | Protein arginine methyltransferase 1 |
| PROCR | 2 | 1 | 2 | 2 | 2 | 1 | Protein C receptor, endothelial |
| PSMA7 | 7 | 7 | 7 | 7 | 8 | 6 | Proteasome (prosome, macropain) subunit, alpha type, 7 |
| PSTPIP1 | 21 | 21 | 24 | 18 | 26 | 25 | Proline-serine-threonine phosphatase interacting protein 1 |
| PTAFR | 42 | 33 | 44 | 39 | 44 | 48 | Platelet-activating factor receptor |
| PTCH1 | 0 | 0 | 0 | 1 | 2 | 1 | Patched 1 |
| PTGES | 2 | 2 | 2 | 2 | 2 | 2 | Prostaglandin E synthase |
| PTGES2 | 1 | 1 | 1 | 1 | 1 | 1 | Prostaglandin E synthase 2 |
| PTK2 | 35 | 41 | 42 | 46 | 47 | 39 | PTK2 protein tyrosine kinase 2 |
| PTK2B | 32 | 44 | 44 | 42 | 46 | 42 | PTK2B protein tyrosine kinase 2 beta |
| PTMA | 0 | 0 | 0 | 0 | 1 | 0 | Prothymosin, alpha |
| PTPN11 | 28 | 30 | 30 | 31 | 33 | 32 | Protein tyrosine phosphatase, non-receptor type 11 |
| PTPN2 | 37 | 37 | 36 | 44 | 39 | 39 | Protein tyrosine phosphatase, non-receptor type 2 |
| PTPN6 | 40 | 41 | 39 | 47 | 41 | 42 | Protein tyrosine phosphatase, non-receptor type 6 |
| PTPRC | 0 | 0 | 0 | 0 | 1 | 0 | Protein tyrosine phosphatase, receptor type, C |
| PTX3 | 7 | 8 | 7 | 7 | 9 | 13 | Pentraxin 3, long |
| PYCARD | 1 | 2 | 0 | 0 | 2 | 1 | PYD and CARD domain containing |
| PYDC1 | 0 | 0 | 0 | 0 | 3 | 2 | PYD (pyrin domain) containing 1 |
| Pcbp1 | 11 | 12 | 12 | 10 | 14 | 13 | Poly(rC) binding protein 1 |
| Pcbp2 | 11 | 12 | 12 | 12 | 15 | 13 | Poly(rC) binding protein 2 |
| Pdcd1 | 0 | 1 | 1 | 0 | 1 | 0 | Programmed cell death 1 |
| Pglyrp1 | 1 | 1 | 0 | 0 | 4 | 2 | Peptidoglycan recognition protein 1 |
| Pglyrp2 | 1 | 1 | 0 | 0 | 1 | 1 | Peptidoglycan recognition protein 2 |
| Pglyrp3 | 1 | 1 | 0 | 0 | 4 | 2 | Peptidoglycan recognition protein 3 |
| Pglyrp4 | 1 | 1 | 0 | 0 | 4 | 2 | Peptidoglycan recognition protein 4 |
| Pik3ap1 | 2 | 3 | 2 | 3 | 2 | 3 | Phosphoinositide-3-kinase adaptor protein 1 |
| Pik3c3 | 5 | 8 | 8 | 7 | 7 | 8 | Phosphoinositide-3-kinase, class 3 |
| Pin1 | 3 | 1 | 3 | 3 | 3 | 2 | Protein (peptidyl-prolyl cis/trans isomerase) NIMA-interacting 1 |
| Plaur | 0 | 0 | 0 | 0 | 2 | 3 | Plasminogen activator, urokinase receptor |
| Plcg2 | 39 | 36 | 38 | 42 | 44 | 40 | Phospholipase C, gamma 2 |
| Plec | 17 | 18 | 20 | 21 | 25 | 25 | Plectin |
| Plg | 25 | 37 | 38 | 36 | 65 | 49 | Plasminogen |
| Pltp | 1 | 2 | 2 | 2 | 3 | 1 | Phospholipid transfer protein |
| Plxna4 | 13 | 16 | 17 | 14 | 19 | 22 | Plexin A4 |
| Pml | 4 | 4 | 5 | 4 | 8 | 4 | Promyelocytic leukemia |
| Polr3a | 3 | 3 | 3 | 4 | 3 | 3 | Polymerase (RNA) III (DNA directed) polypeptide A |
| Polr3b | 3 | 3 | 3 | 3 | 3 | 4 | Polymerase (RNA) III (DNA directed) polypeptide B |
| Polr3c | 0 | 0 | 1 | 1 | 1 | 0 | Polymerase (RNA) III (DNA directed) polypeptide C |
| Polr3d | 1 | 0 | 0 | 0 | 1 | 1 | Polymerase (RNA) III (DNA directed) polypeptide D |
| Polr3e | 1 | 1 | 1 | 1 | 1 | 1 | Polymerase (RNA) III (DNA directed) polypeptide E |
| Polr3h | 1 | 1 | 1 | 1 | 1 | 1 | Polymerase (RNA) III (DNA directed) polypeptide H |
| Pparg | 30 | 34 | 35 | 48 | 51 | 43 | Peroxisome proliferator activated receptor gamma |
| Ppargc1a | 1 | 2 | 2 | 2 | 2 | 2 | Peroxisome proliferative activated receptor, gamma, coactivator 1 alpha |
| Prkcd | 33 | 39 | 40 | 40 | 55 | 43 | Protein kinase C, delta |
| Ptch1 | 2 | 1 | 1 | 1 | 2 | 1 | Patched homolog 1 |
| Ptges | 2 | 2 | 2 | 2 | 2 | 2 | Prostaglandin E synthase |
| Ptx3 | 7 | 8 | 7 | 7 | 9 | 13 | Pentraxin related gene |
| Pycard | 1 | 2 | 0 | 0 | 4 | 2 | PYD and CARD domain containing |
| RAB11A | 33 | 36 | 39 | 41 | 56 | 45 | RAB11A, member RAS oncogene family |
| RAC2 | 8 | 9 | 10 | 10 | 10 | 11 | Ras-related C3 botulinum toxin substrate 2 (rho family, small GTP binding protein Rac2) |
| RAG1 | 1 | 1 | 1 | 1 | 1 | 1 | Recombination activating gene 1 |
| RANBP9 | 2 | 2 | 2 | 3 | 3 | 3 | RAN binding protein 9 |
| RARRES2 | 0 | 0 | 1 | 0 | 1 | 0 | Retinoic acid receptor responder (tazarotene induced) 2 |
| RASGEF1B | 3 | 3 | 3 | 4 | 3 | 3 | RasGEF domain family, member 1B |
| RBCK1 | 1 | 0 | 1 | 0 | 2 | 2 | RanBP-type and C3HC4-type zinc finger containing 1 |
| RCAN1 | 2 | 3 | 3 | 2 | 3 | 2 | Regulator of calcineurin 1 |
| REL | 4 | 3 | 3 | 4 | 5 | 4 | v-rel reticuloendotheliosis viral oncogene homolog (avian) |
| RELA | 4 | 3 | 3 | 4 | 5 | 4 | v-rel reticuloendotheliosis viral oncogene homolog A (avian) |
| RELB | 4 | 4 | 3 | 4 | 5 | 4 | v-rel reticuloendotheliosis viral oncogene homolog B |
| REST | 6 | 12 | 12 | 41 | 121 | 58 | RE1-silencing transcription factor |
| RETNLB | 0 | 0 | 0 | 0 | 2 | 0 | Resistin like beta |
| RFTN1 | 2 | 2 | 2 | 2 | 2 | 2 | Raftlin, lipid raft linker 1 |
| RICTOR | 1 | 1 | 1 | 1 | 1 | 1 | RPTOR independent companion of MTOR, complex 2 |
| RIPK1 | 1 | 2 | 2 | 2 | 1 | 1 | Receptor (TNFRSF)-interacting serine-threonine kinase 1 |
| RIPK2 | 35 | 41 | 41 | 39 | 52 | 42 | Receptor-interacting serine-threonine kinase 2 |
| RNASE7 | 0 | 0 | 0 | 0 | 10 | 1 | Ribonuclease, RNase A family, 7 |
| RNASEL | 2 | 3 | 2 | 3 | 2 | 2 | Ribonuclease L (2',5'-oligoisoadenylate synthetase-dependent) |
| RNF125 | 3 | 4 | 4 | 3 | 7 | 5 | Ring finger protein 125 |
| RNF135 | 14 | 11 | 13 | 10 | 40 | 27 | Ring finger protein 135 |
| RNF31 | 3 | 3 | 3 | 2 | 6 | 6 | Ring finger protein 31 |
| RNF41 | 6 | 4 | 5 | 6 | 6 | 4 | Ring finger protein 41 |
| RNF5 | 2 | 2 | 2 | 2 | 9 | 1 | Ring finger protein 5 |
| RPL19 | 1 | 1 | 1 | 1 | 1 | 2 | Ribosomal protein L19 |
| RPS19 | 0 | 0 | 0 | 0 | 1 | 0 | Ribosomal protein S19 |
| RPS6KA1 | 31 | 41 | 39 | 41 | 53 | 45 | Ribosomal protein S6 kinase, 90kDa, polypeptide 1 |
| RPS6KA2 | 37 | 37 | 37 | 43 | 52 | 44 | Ribosomal protein S6 kinase, 90kDa, polypeptide 2 |
| RPS6KA3 | 38 | 39 | 39 | 43 | 49 | 42 | Ribosomal protein S6 kinase, 90kDa, polypeptide 3 |
| RPS6KA4 | 35 | 41 | 38 | 43 | 52 | 41 | Ribosomal protein S6 kinase, 90kDa, polypeptide 4 |
| RPS6KA5 | 0 | 0 | 0 | 0 | 39 | 0 | Ribosomal protein S6 kinase, 90kDa, polypeptide 5 |
| RSAD2 | 1 | 1 | 1 | 1 | 1 | 1 | Radical S-adenosyl methionine domain containing 2 |
| RUNX3 | 3 | 3 | 3 | 4 | 3 | 3 | Runt-related transcription factor 3 |
| RXRA | 34 | 33 | 38 | 46 | 52 | 47 | Retinoid X receptor, alpha |
| Rac2 | 36 | 32 | 41 | 44 | 49 | 48 | RAS-related C3 botulinum substrate 2 |
| Raet1d | 0 | 0 | 0 | 0 | 2 | 0 | Retinoic acid early transcript delta |
| Raet1e | 0 | 0 | 0 | 0 | 6 | 0 | Retinoic acid early transcript gamma |
| Rag1 | 1 | 1 | 1 | 1 | 1 | 2 | Recombination activating gene 1 |
| Rarres2 | 0 | 0 | 1 | 0 | 1 | 0 | Retinoic acid receptor responder (tazarotene induced) 2 |
| Rela | 3 | 3 | 3 | 4 | 5 | 3 | v-rel reticuloendotheliosis viral oncogene homolog A (avian) |
| Relb | 0 | 0 | 0 | 0 | 1 | 0 | Avian reticuloendotheliosis viral (v-rel) oncogene related B |
| Rgmb | 3 | 2 | 2 | 3 | 3 | 4 | RGM domain family, member B |
| Rictor | 1 | 1 | 1 | 1 | 1 | 1 | RPTOR independent companion of MTOR, complex 2 |
| Ripk2 | 36 | 38 | 41 | 38 | 55 | 42 | Receptor (TNFRSF)-interacting serine-threonine kinase 2 |
| Ripk3 | 34 | 41 | 43 | 37 | 54 | 41 | Receptor-interacting serine-threonine kinase 3 |
| Rnasel | 1 | 1 | 1 | 0 | 2 | 1 | Ribonuclease L (2', 5'-oligoisoadenylate synthetase-dependent) |
| Rnf135 | 2 | 3 | 4 | 4 | 6 | 7 | Ring finger protein 135 |
| Rora | 31 | 36 | 35 | 47 | 51 | 42 | RAR-related orphan receptor alpha |
| Rxra | 34 | 33 | 38 | 46 | 52 | 47 | Retinoid X receptor alpha |
| S100A12 | 5 | 8 | 4 | 5 | 14 | 5 | S100 calcium binding protein A12 |
| S100A7 | 2 | 2 | 0 | 0 | 4 | 1 | S100 calcium binding protein A7 |
| S100A9 | 5 | 9 | 5 | 5 | 15 | 5 | S100 calcium binding protein A9 |
| S100B | 7 | 10 | 4 | 6 | 21 | 6 | S100 calcium binding protein B |
| S100a10 | 4 | 8 | 4 | 7 | 11 | 5 | S100 calcium binding protein A10 (calpactin) |
| S100a8 | 4 | 6 | 4 | 5 | 13 | 4 | S100 calcium binding protein A8 (calgranulin A) |
| S100a9 | 5 | 9 | 5 | 5 | 16 | 5 | S100 calcium binding protein A9 (calgranulin B) |
| SAMHD1 | 1 | 1 | 1 | 1 | 1 | 1 | SAM domain and HD domain 1 |
| SARM1 | 1 | 1 | 1 | 1 | 1 | 1 | Sterile alpha and TIR motif containing 1 |
| SCAMP5 | 4 | 2 | 4 | 5 | 4 | 4 | Secretory carrier membrane protein 5 |
| SCARF1 | 30 | 35 | 37 | 30 | 44 | 31 | Scavenger receptor class F, member 1 |
| SELE | 15 | 20 | 21 | 22 | 51 | 27 | Selectin E |
| SELK | 1 | 1 | 1 | 1 | 1 | 0 | Selenoprotein K |
| SENP2 | 4 | 4 | 5 | 5 | 6 | 6 | SUMO1/sentrin/SMT3 specific peptidase 2 |
| SERPINB9 | 20 | 24 | 25 | 27 | 35 | 28 | Serpin peptidase inhibitor, clade B (ovalbumin), member 9 |
| SERPINE1 | 19 | 22 | 22 | 22 | 33 | 25 | Serpin peptidase inhibitor, clade E (nexin, plasminogen activator inhibitor type 1), member 1 |
| SERPING1 | 0 | 0 | 0 | 0 | 1 | 0 | Serpin peptidase inhibitor, clade G (C1 inhibitor), member 1 |
| SFTPA1 | 11 | 13 | 11 | 15 | 22 | 16 | Surfactant protein A1 |
| SFTPA2 | 29 | 34 | 26 | 26 | 36 | 32 | Surfactant protein A2 |
| SFTPD | 34 | 37 | 39 | 41 | 49 | 50 | Surfactant protein D |
| SH2D1A | 3 | 4 | 3 | 4 | 4 | 1 | SH2 domain containing 1A |
| SIAH1 | 2 | 3 | 3 | 2 | 4 | 2 | Seven in absentia homolog 1 (Drosophila) |
| SIGIRR | 6 | 9 | 9 | 9 | 9 | 10 | Single immunoglobulin and toll-interleukin 1 receptor (TIR) domain |
| SIGLEC1 | 30 | 37 | 37 | 34 | 74 | 38 | Sialic acid binding Ig-like lectin 1, sialoadhesin |
| SIGLEC10 | 20 | 22 | 24 | 21 | 60 | 35 | Sialic acid binding Ig-like lectin 10 |
| SIGLEC11 | 16 | 21 | 22 | 20 | 45 | 29 | Sialic acid binding Ig-like lectin 11 |
| SIGLEC15 | 1 | 1 | 1 | 1 | 1 | 1 | Sialic acid binding Ig-like lectin 15 |
| SIGLEC5 | 6 | 3 | 6 | 5 | 25 | 14 | Sialic acid binding Ig-like lectin 5 |
| SIGLEC6 | 3 | 2 | 2 | 1 | 17 | 8 | Sialic acid binding Ig-like lectin 6 |
| SIGLEC7 | 1 | 0 | 0 | 0 | 13 | 6 | Sialic acid binding Ig-like lectin 7 |
| SIGLEC8 | 1 | 0 | 0 | 0 | 18 | 8 | Sialic acid binding Ig-like lectin 8 |
| SIGLEC9 | 1 | 0 | 0 | 0 | 15 | 7 | Sialic acid binding Ig-like lectin 9 |
| SIRPA | 2 | 4 | 5 | 5 | 8 | 8 | Signal-regulatory protein alpha |
| SIRT1 | 4 | 5 | 4 | 4 | 6 | 4 | Sirtuin 1 |
| SIVA1 | 0 | 0 | 1 | 1 | 1 | 1 | SIVA1, apoptosis-inducing factor |
| SLAMF1 | 1 | 1 | 2 | 1 | 7 | 0 | Signaling lymphocytic activation molecule family member 1 |
| SLAMF6 | 0 | 0 | 0 | 0 | 3 | 0 | SLAM family member 6 |
| SLAMF7 | 0 | 0 | 0 | 0 | 5 | 0 | SLAM family member 7 |
| SLC2A11 | 14 | 16 | 18 | 20 | 15 | 18 | Solute carrier family 2 (facilitated glucose transporter), member 11 |
| SMAD6 | 8 | 5 | 8 | 8 | 8 | 8 | SMAD family member 6 |
| SMAD7 | 8 | 6 | 8 | 9 | 8 | 7 | SMAD family member 7 |
| SMARCA2 | 6 | 8 | 8 | 7 | 8 | 6 | SWI/SNF related, matrix associated, actin dependent regulator of chromatin, subfamily a, member 2 |
| SMARCA4 | 30 | 33 | 33 | 37 | 35 | 34 | SWI/SNF related, matrix associated, actin dependent regulator of chromatin, subfamily a, member 4 |
| SMARCE1 | 4 | 5 | 3 | 5 | 6 | 5 | SWI/SNF related, matrix associated, actin dependent regulator of chromatin, subfamily e, member 1 |
| SNAP23 | 2 | 2 | 2 | 3 | 2 | 4 | Synaptosomal-associated protein, 23kDa |
| SNCA | 2 | 3 | 3 | 2 | 3 | 3 | Synuclein, alpha (non A4 component of amyloid precursor) |
| SOCS1 | 8 | 5 | 8 | 9 | 8 | 9 | Suppressor of cytokine signaling 1 |
| SOCS2 | 9 | 5 | 9 | 9 | 8 | 9 | Suppressor of cytokine signaling 2 |
| SOCS3 | 8 | 4 | 8 | 8 | 7 | 9 | Suppressor of cytokine signaling 3 |
| SOCS5 | 8 | 5 | 8 | 8 | 7 | 7 | Suppressor of cytokine signaling 5 |
| SOCS6 | 11 | 8 | 11 | 11 | 11 | 13 | Suppressor of cytokine signaling 6 |
| SPHK1 | 4 | 3 | 4 | 5 | 5 | 6 | Sphingosine kinase 1 |
| SPI1 | 1 | 1 | 1 | 1 | 1 | 1 | Spleen focus forming virus (SFFV) proviral integration oncogene spi1 |
| SPON2 | 4 | 3 | 3 | 5 | 2 | 4 | Spondin 2, extracellular matrix protein |
| SPP1 | 0 | 1 | 1 | 1 | 1 | 1 | Secreted phosphoprotein 1 |
| SQSTM1 | 1 | 1 | 1 | 2 | 1 | 1 | Sequestosome 1 |
| SRC | 37 | 41 | 45 | 42 | 47 | 38 | v-src sarcoma (Schmidt-Ruppin A-2) viral oncogene homolog (avian) |
| SREBF1 | 2 | 2 | 2 | 4 | 5 | 4 | Sterol regulatory element binding transcription factor 1 |
| SREBF2 | 1 | 1 | 1 | 2 | 2 | 2 | Sterol regulatory element binding transcription factor 2 |
| SRPK1 | 19 | 25 | 23 | 24 | 29 | 29 | SRSF protein kinase 1 |
| STAP2 | 1 | 1 | 1 | 0 | 2 | 1 | Signal transducing adaptor family member 2 |
| STAT1 | 1 | 4 | 3 | 3 | 6 | 4 | Signal transducer and activator of transcription 1, 91kDa |
| STAT2 | 1 | 2 | 2 | 2 | 3 | 3 | Signal transducer and activator of transcription 2, 113kDa |
| STAT3 | 2 | 6 | 3 | 4 | 7 | 6 | Signal transducer and activator of transcription 3 (acute-phase response factor) |
| STAT4 | 1 | 1 | 0 | 0 | 1 | 0 | Signal transducer and activator of transcription 4 |
| STAT5A | 1 | 5 | 3 | 4 | 7 | 6 | Signal transducer and activator of transcription 5A |
| STAT5B | 1 | 4 | 3 | 3 | 6 | 4 | Signal transducer and activator of transcription 5B |
| STAT6 | 1 | 5 | 3 | 4 | 7 | 5 | Signal transducer and activator of transcription 6, interleukin-4 induced |
| STUB1 | 16 | 18 | 17 | 19 | 17 | 13 | STIP1 homology and U-box containing protein 1, E3 ubiquitin protein ligase |
| SUGT1 | 2 | 2 | 2 | 2 | 2 | 1 | SGT1, suppressor of G2 allele of SKP1 (S. cerevisiae) |
| SYK | 35 | 40 | 43 | 42 | 52 | 38 | Spleen tyrosine kinase |
| SYP | 1 | 2 | 2 | 4 | 4 | 5 | Synaptophysin |
| Samhd1 | 1 | 1 | 1 | 1 | 1 | 1 | SAM domain and HD domain, 1 |
| Sema3a | 13 | 11 | 13 | 16 | 18 | 15 | Sema domain, immunoglobulin domain (Ig), short basic domain, secreted, (semaphorin) 3A |
| Senp2 | 4 | 4 | 5 | 5 | 6 | 6 | SUMO/sentrin specific peptidase 2 |
| Serpinb9 | 20 | 24 | 25 | 27 | 35 | 28 | Serine (or cysteine) peptidase inhibitor, clade B, member 9 |
| Serpine1 | 20 | 24 | 25 | 25 | 35 | 28 | Serine (or cysteine) peptidase inhibitor, clade E, member 1 |
| Sftpa1 | 29 | 37 | 32 | 28 | 43 | 37 | Surfactant associated protein A1 |
| Sftpd | 13 | 13 | 13 | 17 | 22 | 16 | Surfactant associated protein D |
| Sigirr | 7 | 8 | 9 | 9 | 9 | 10 | Single immunoglobulin and toll-interleukin 1 receptor (TIR) domain |
| Siglec1 | 30 | 34 | 37 | 32 | 78 | 39 | Sialic acid binding Ig-like lectin 1, sialoadhesin |
| Siglec5 | 5 | 10 | 9 | 9 | 28 | 15 | Sialic acid binding Ig-like lectin 5 |
| Siglece | 1 | 0 | 0 | 1 | 17 | 8 | Sialic acid binding Ig-like lectin E |
| Siglech | 1 | 0 | 0 | 0 | 15 | 6 | Sialic acid binding Ig-like lectin H |
| Sirt1 | 4 | 5 | 4 | 4 | 6 | 5 | Sirtuin 1 (silent mating type information regulation 2, homolog) 1 (S. cerevisiae) |
| Slc15a4 | 1 | 2 | 2 | 2 | 4 | 2 | Solute carrier family 15, member 4 |
| Snca | 2 | 3 | 3 | 2 | 3 | 3 | Synuclein, alpha |
| Socs1 | 8 | 5 | 8 | 9 | 8 | 8 | Suppressor of cytokine signaling 1 |
| Spon2 | 3 | 2 | 2 | 4 | 1 | 3 | Spondin 2, extracellular matrix protein |
| Spp1 | 0 | 0 | 0 | 0 | 1 | 1 | Secreted phosphoprotein 1 |
| Srebf1 | 1 | 3 | 2 | 4 | 4 | 4 | Sterol regulatory element binding transcription factor 1 |
| Srebf2 | 2 | 3 | 2 | 4 | 6 | 5 | Sterol regulatory element binding factor 2 |
| Srxn1 | 0 | 0 | 0 | 1 | 1 | 0 | Sulfiredoxin 1 homolog (S. cerevisiae) |
| Stat1 | 1 | 5 | 3 | 4 | 7 | 5 | Signal transducer and activator of transcription 1 |
| Stat4 | 2 | 6 | 3 | 4 | 7 | 6 | Signal transducer and activator of transcription 4 |
| Stat6 | 0 | 0 | 0 | 0 | 1 | 0 | Signal transducer and activator of transcription 6 |
| Stim1 | 2 | 2 | 3 | 2 | 2 | 2 | Stromal interaction molecule 1 |
| Stub1 | 15 | 18 | 16 | 19 | 17 | 14 | STIP1 homology and U-Box containing protein 1 |
| TAB2 | 1 | 2 | 2 | 2 | 2 | 2 | TGF-beta activated kinase 1/MAP3K7 binding protein 2 |
| TAB3 | 1 | 2 | 2 | 2 | 2 | 2 | TGF-beta activated kinase 1/MAP3K7 binding protein 3 |
| TANK | 1 | 1 | 1 | 2 | 1 | 1 | TRAF family member-associated NFKB activator |
| TAX1BP1 | 2 | 2 | 2 | 1 | 2 | 2 | Tax1 (human T-cell leukemia virus type I) binding protein 1 |
| TBKBP1 | 1 | 1 | 1 | 1 | 2 | 2 | TBK1 binding protein 1 |
| TCEB1 | 1 | 1 | 1 | 1 | 47 | 1 | Transcription elongation factor B (SIII), polypeptide 1 (15kDa, elongin C) |
| TCF4 | 3 | 2 | 2 | 2 | 3 | 4 | Transcription factor 4 |
| TECPR1 | 2 | 2 | 2 | 2 | 2 | 1 | Tectonin beta-propeller repeat containing 1 |
| TICAM1 | 1 | 1 | 0 | 1 | 3 | 1 | Toll-like receptor adaptor molecule 1 |
| TICAM2 | 3 | 5 | 5 | 4 | 11 | 7 | Toll-like receptor adaptor molecule 2 |
| TIRAP | 2 | 2 | 2 | 2 | 1 | 2 | Toll-interleukin 1 receptor (TIR) domain containing adaptor protein |
| TLR1 | 15 | 16 | 18 | 13 | 20 | 27 | Toll-like receptor 1 |
| TLR10 | 19 | 17 | 22 | 23 | 28 | 31 | Toll-like receptor 10 |
| TLR2 | 36 | 34 | 39 | 38 | 51 | 52 | Toll-like receptor 2 |
| TLR3 | 38 | 35 | 39 | 40 | 47 | 51 | Toll-like receptor 3 |
| TLR5 | 35 | 37 | 42 | 36 | 54 | 46 | Toll-like receptor 5 |
| TLR6 | 10 | 9 | 11 | 8 | 15 | 18 | Toll-like receptor 6 |
| TLR8 | 37 | 36 | 37 | 38 | 49 | 53 | Toll-like receptor 8 |
| TLR9 | 35 | 33 | 41 | 40 | 48 | 53 | Toll-like receptor 9 |
| TNF | 3 | 3 | 5 | 3 | 26 | 4 | Tumor necrosis factor |
| TNFAIP3 | 3 | 4 | 4 | 4 | 4 | 3 | Tumor necrosis factor, alpha-induced protein 3 |
| TNFAIP8L2 | 3 | 3 | 3 | 3 | 4 | 3 | Tumor necrosis factor, alpha-induced protein 8-like 2 |
| TNFRSF12A | 0 | 0 | 0 | 0 | 1 | 0 | Tumor necrosis factor receptor superfamily, member 12A |
| TNFRSF18 | 5 | 2 | 2 | 3 | 3 | 2 | Tumor necrosis factor receptor superfamily, member 18 |
| TNFRSF1B | 0 | 0 | 0 | 0 | 1 | 0 | Tumor necrosis factor receptor superfamily, member 1B |
| TNFSF11 | 5 | 6 | 6 | 6 | 4 | 3 | Tumor necrosis factor (ligand) superfamily, member 11 |
| TNFSF9 | 0 | 0 | 0 | 0 | 1 | 0 | Tumor necrosis factor (ligand) superfamily, member 9 |
| TNIP1 | 2 | 3 | 2 | 2 | 2 | 2 | TNFAIP3 interacting protein 1 |
| TNIP3 | 2 | 2 | 2 | 2 | 2 | 2 | TNFAIP3 interacting protein 3 |
| TOLLIP | 1 | 1 | 1 | 1 | 1 | 1 | Toll interacting protein |
| TOMM70A | 18 | 17 | 19 | 18 | 18 | 21 | Translocase of outer mitochondrial membrane 70 homolog A (S. cerevisiae) |
| TP53 | 1 | 2 | 2 | 2 | 3 | 2 | Tumor protein p53 |
| TP63 | 2 | 2 | 2 | 2 | 3 | 4 | Tumor protein p63 |
| TPSB2 | 24 | 35 | 38 | 35 | 71 | 47 | Tryptase beta 2 (gene/pseudogene) |
| TPST1 | 2 | 2 | 2 | 3 | 2 | 0 | Tyrosylprotein sulfotransferase 1 |
| TRAF1 | 6 | 5 | 5 | 5 | 6 | 7 | TNF receptor-associated factor 1 |
| TRAF2 | 7 | 7 | 7 | 5 | 6 | 7 | TNF receptor-associated factor 2 |
| TRAF3 | 7 | 7 | 7 | 7 | 8 | 7 | TNF receptor-associated factor 3 |
| TRAF5 | 6 | 5 | 5 | 5 | 6 | 6 | TNF receptor-associated factor 5 |
| TRAFD1 | 2 | 1 | 2 | 2 | 2 | 1 | TRAF-type zinc finger domain containing 1 |
| TREM1 | 0 | 0 | 0 | 0 | 1 | 0 | Triggering receptor expressed on myeloid cells 1 |
| TREM2 | 2 | 2 | 2 | 1 | 3 | 1 | Triggering receptor expressed on myeloid cells 2 |
| TREML1 | 0 | 0 | 0 | 1 | 1 | 0 | Triggering receptor expressed on myeloid cells-like 1 |
| TREML2 | 2 | 2 | 2 | 1 | 3 | 0 | Triggering receptor expressed on myeloid cells-like 2 |
| TRIB2 | 5 | 6 | 6 | 5 | 10 | 11 | Tribbles homolog 2 (Drosophila) |
| TRIB3 | 34 | 38 | 38 | 41 | 55 | 44 | Tribbles homolog 3 (Drosophila) |
| TRIM21 | 27 | 16 | 20 | 10 | 109 | 68 | Tripartite motif containing 21 |
| TRIM22 | 15 | 10 | 14 | 5 | 83 | 47 | Tripartite motif containing 22 |
| TRIM23 | 0 | 0 | 0 | 0 | 1 | 0 | Tripartite motif containing 23 |
| TRIM25 | 27 | 19 | 27 | 19 | 90 | 68 | Tripartite motif containing 25 |
| TRIM32 | 12 | 8 | 12 | 8 | 57 | 29 | Tripartite motif containing 32 |
| TRIM5 | 20 | 18 | 23 | 12 | 115 | 62 | Tripartite motif containing 5 |
| TRPM2 | 1 | 2 | 2 | 1 | 2 | 2 | Transient receptor potential cation channel, subfamily M, member 2 |
| TYK2 | 33 | 41 | 43 | 40 | 51 | 42 | Tyrosine kinase 2 |
| TYRO3 | 28 | 43 | 47 | 40 | 51 | 41 | TYRO3 protein tyrosine kinase |
| Tank | 1 | 1 | 2 | 3 | 1 | 2 | TRAF family member-associated Nf-kappa B activator |
| Tbk1 | 29 | 35 | 44 | 40 | 59 | 43 | TANK-binding kinase 1 |
| Tbkbp1 | 1 | 1 | 1 | 1 | 2 | 2 | TBK1 binding protein 1 |
| Tbx21 | 14 | 14 | 14 | 14 | 17 | 16 | T-box 21 |
| Ticam1 | 1 | 1 | 0 | 1 | 3 | 1 | Toll-like receptor adaptor molecule 1 |
| Tirap | 2 | 2 | 2 | 1 | 1 | 4 | Toll-interleukin 1 receptor (TIR) domain-containing adaptor protein |
| Tlr1 | 21 | 22 | 28 | 19 | 28 | 29 | Toll-like receptor 1 |
| Tlr11 | 36 | 38 | 40 | 38 | 53 | 45 | Toll-like receptor 11 |
| Tlr13 | 38 | 38 | 40 | 39 | 47 | 48 | Toll-like receptor 13 |
| Tlr3 | 33 | 39 | 42 | 38 | 47 | 51 | Toll-like receptor 3 |
| Tlr5 | 34 | 38 | 43 | 37 | 50 | 48 | Toll-like receptor 5 |
| Tlr6 | 25 | 22 | 27 | 23 | 26 | 32 | Toll-like receptor 6 |
| Tlr8 | 35 | 33 | 42 | 39 | 51 | 50 | Toll-like receptor 8 |
| Tlr9 | 35 | 34 | 42 | 43 | 46 | 50 | Toll-like receptor 9 |
| Tmem173 | 1 | 1 | 1 | 1 | 1 | 1 | Transmembrane protein 173 |
| Tnf | 2 | 2 | 3 | 2 | 26 | 2 | Tumor necrosis factor |
| Tnfaip3 | 1 | 1 | 1 | 1 | 1 | 1 | Tumor necrosis factor, alpha-induced protein 3 |
| Tnfaip8l2 | 3 | 3 | 3 | 3 | 4 | 3 | Tumor necrosis factor, alpha-induced protein 8-like 2 |
| Tnfrsf1a | 1 | 1 | 1 | 1 | 1 | 0 | Tumor necrosis factor receptor superfamily, member 1a |
| Tnfrsf9 | 7 | 6 | 7 | 5 | 6 | 1 | Tumor necrosis factor receptor superfamily, member 9 |
| Tnfsf11 | 5 | 6 | 5 | 6 | 4 | 3 | Tumor necrosis factor (ligand) superfamily, member 11 |
| Tnfsf4 | 0 | 0 | 0 | 0 | 1 | 0 | Tumor necrosis factor (ligand) superfamily, member 4 |
| Tnfsf9 | 0 | 0 | 0 | 0 | 1 | 0 | Tumor necrosis factor (ligand) superfamily, member 9 |
| Tnip1 | 2 | 3 | 2 | 2 | 2 | 2 | TNFAIP3 interacting protein 1 |
| Tollip | 1 | 1 | 1 | 1 | 1 | 1 | Toll interacting protein |
| Traf3 | 8 | 7 | 7 | 8 | 9 | 7 | TNF receptor-associated factor 3 |
| Treml2 | 1 | 2 | 2 | 1 | 3 | 0 | Triggering receptor expressed on myeloid cells-like 2 |
| Trib3 | 36 | 36 | 38 | 40 | 53 | 47 | Tribbles homolog 3 (Drosophila) |
| Tril | 41 | 40 | 42 | 34 | 49 | 44 | TLR4 interactor with leucine-rich repeats |
| Trim25 | 2 | 2 | 3 | 2 | 3 | 5 | Tripartite motif-containing 25 |
| Trim30a | 12 | 11 | 19 | 9 | 104 | 63 | Tripartite motif-containing 30A |
| Trim56 | 10 | 11 | 15 | 13 | 21 | 22 | Tripartite motif-containing 56 |
| Trp53 | 2 | 2 | 2 | 2 | 3 | 3 | Transformation related protein 53 |
| Trp63 | 2 | 2 | 2 | 2 | 3 | 4 | Transformation related protein 63 |
| Trpm2 | 9 | 13 | 12 | 13 | 12 | 15 | Transient receptor potential cation channel, subfamily M, member 2 |
| Tyk2 | 33 | 41 | 41 | 40 | 53 | 42 | Tyrosine kinase 2 |
| UBA52 | 4 | 5 | 6 | 10 | 29 | 14 | Ubiquitin A-52 residue ribosomal protein fusion product 1 |
| UBB | 4 | 5 | 6 | 9 | 21 | 11 | Ubiquitin B |
| UBC | 3 | 4 | 5 | 7 | 18 | 9 | Ubiquitin C |
| UBD | 1 | 3 | 2 | 3 | 11 | 3 | Ubiquitin D |
| UBE2D3 | 17 | 26 | 24 | 28 | 32 | 25 | Ubiquitin-conjugating enzyme E2D 3 |
| UBE2L6 | 14 | 20 | 20 | 21 | 28 | 21 | Ubiquitin-conjugating enzyme E2L 6 |
| UBE2N | 13 | 23 | 23 | 23 | 29 | 24 | Ubiquitin-conjugating enzyme E2N |
| UBE2V1 | 2 | 2 | 2 | 2 | 4 | 2 | Ubiquitin-conjugating enzyme E2 variant 1 |
| UBQLN1 | 3 | 2 | 2 | 2 | 5 | 2 | Ubiquilin 1 |
| UNC5CL | 5 | 5 | 5 | 7 | 6 | 5 | unc-5 homolog C (C. elegans)-like |
| UNC93B1 | 2 | 2 | 2 | 2 | 3 | 3 | unc-93 homolog B1 (C. elegans) |
| USP7 | 22 | 24 | 24 | 28 | 48 | 28 | Ubiquitin specific peptidase 7 (herpes virus-associated) |
| Ubqln1 | 3 | 2 | 2 | 2 | 5 | 2 | Ubiquilin 1 |
| Unc5cl | 0 | 0 | 0 | 0 | 1 | 0 | unc-5 homolog C (C. elegans)-like |
| Unc93b1 | 2 | 2 | 2 | 2 | 3 | 3 | unc-93 homolog B1 (C. elegans) |
| Usp4 | 26 | 27 | 28 | 39 | 58 | 43 | Ubiquitin specific peptidase 4 (proto-oncogene) |
| VASP | 4 | 4 | 5 | 4 | 7 | 7 | Vasodilator-stimulated phosphoprotein |
| VDR | 19 | 23 | 24 | 30 | 33 | 33 | Vitamin D (1,25- dihydroxyvitamin D3) receptor |
| VENTX | 20 | 24 | 44 | 43 | 69 | 50 | VENT homeobox |
| VNN1 | 3 | 3 | 3 | 4 | 4 | 2 | Vanin 1 |
| Vegfa | 4 | 4 | 3 | 4 | 5 | 4 | Vascular endothelial growth factor A |
| Vldlr | 39 | 40 | 41 | 41 | 46 | 43 | Very low density lipoprotein receptor |
| Vnn1 | 4 | 3 | 4 | 5 | 4 | 2 | Vanin 1 |
| WDR34 | 1 | 1 | 1 | 1 | 1 | 0 | WD repeat domain 34 |
| WDR62 | 8 | 8 | 9 | 7 | 12 | 13 | WD repeat domain 62 |
| XBP1 | 1 | 1 | 1 | 1 | 1 | 0 | X-box binding protein 1 |
| XIAP | 7 | 8 | 10 | 9 | 9 | 7 | X-linked inhibitor of apoptosis |
| Xbp1 | 1 | 1 | 1 | 1 | 1 | 0 | X-box binding protein 1 |
| Xiap | 7 | 8 | 10 | 9 | 9 | 7 | X-linked inhibitor of apoptosis |
| YWHAE | 7 | 7 | 6 | 8 | 7 | 6 | Tyrosine 3-monooxygenase/tryptophan 5-monooxygenase activation protein, epsilon polypeptide |
| Yy1 | 7 | 6 | 15 | 27 | 88 | 107 | YY1 transcription factor |
| ZBP1 | 0 | 0 | 0 | 0 | 1 | 0 | Z-DNA binding protein 1 |
| ZC3HAV1 | 2 | 4 | 3 | 3 | 2 | 4 | Zinc finger CCCH-type, antiviral 1 |
| ZFPM1 | 27 | 26 | 26 | 30 | 85 | 56 | Zinc finger protein, multitype 1 |
| ZFPM2 | 3 | 2 | 3 | 3 | 3 | 2 | Zinc finger protein, multitype 2 |
| ZMYND11 | 2 | 2 | 2 | 3 | 2 | 2 | Zinc finger, MYND-type containing 11 |
| Zbp1 | 1 | 1 | 0 | 0 | 1 | 0 | Z-DNA binding protein 1 |
| Zc3hav1 | 3 | 3 | 3 | 3 | 3 | 3 | Zinc finger CCCH type, antiviral 1 |
| Zfpm1 | 27 | 28 | 30 | 28 | 89 | 48 | Zinc finger protein, multitype 1 |
| Zfpm2 | 6 | 3 | 4 | 4 | 8 | 5 | Zinc finger protein, multitype 2 |
| Total | 2,838 | 3,119 | 3,382 | 3,469 | 5,208 | 3,955 |  |

**Table S17. RIG-I gene alignment results for six species.**

| **Species** | **NCBI (RIG-I)** | **Gene (prediction)** | **Identity** | **E value** | **Bit score** |
| --- | --- | --- | --- | --- | --- |
| Goose | JF804977 | ACY_005623 | 98.88 | 0 | 1834 |
| Duck | EU363349 | Apl2_17430 | 98.13 | 0 | 1651 |
| Zebra finch | XM_002194524 | ENSTGUP00000001567 | 98.68 | 0 | 1815 |
| Human | AF038963 | ENSP00000369213 | 99.89 | 0 | 1919 |
| Chicken | - | ENSGALP00000018045 | 34.18 | 3e-100 | 362 |
| Turkey | - | ENSMGAP00000011342 | 33.74 | 3e-104 | 375 |

RIG-I gene sequences of six species were downloaded from NCBI. The RIG-I gene sequences of chicken and turkey were not found. A Blast alignment was performed with an E value cutoff of 1e-5 to align the genes, and the best alignments were selected.

**Table S18. Alignment information for the goose, duck, and zebra-finch RIG-I genes.**

| **Type** | **RIG-I** | | |
| --- | --- | --- | --- |
|  | **Zebra finch** | **Goose** | **Duck** |
| Gene | ENSTGUP00000001567 | ACY_005623 | Apl2_17430 |
| Gene length (bp) | 21,043 | 21,024 | 19,165 |
| CDS length (bp) | 2,730 | 2,673 | 2,545 |
| Exon number | 20 | 17 | 17 |
| Protein IPR domain | IPR014001:Helicase, superfamily 1/2, ATP-binding domain | IPR014001:Helicase, superfamily 1/2, ATP-binding domain | IPR014001; Helicase, superfamily 1/2, ATP-binding domain |
|  | IPR001650:Helicase, C-terminal | IPR001650:Helicase, C-terminal | IPR001650; Helicase, C-terminal |
|  | IPR021673:C-terminal domain of RIG-I | IPR021673:C-terminal domain of RIG-I | IPR021673; C-terminal domain of RIG-I |
|  | IPR011545:DNA/RNA helicase, DEAD/DEAH box type, N-terminal | IPR011545:DNA/RNA helicase, DEAD/DEAH box type, N-terminal | IPR011545; DNA/RNA helicase, DEAD/DEAH box type, N-terminal |
|  | IPR006935:Helicase/UvrB domain |  |  |

**Table S19. Alignment information for RIG-I gene fragments of chicken and turkey, using blast.**

| **Species** | **Target ID** | **JF804977** | | **XM_002194524** | | **EU363349** | | **Target start** |
| --- | --- | --- | --- | --- | --- | --- | --- | --- |
|  |  | **Match length** | **Align length** | **Match length** | **Align length** | **Match length** | **Align length** |  |
| Chicken | 7 | 43 | 28,15,14, | 22 | 34, | 44 | 30,15,14, | 22623253 |
| Turkey | 7 | 32 | 28,16, | 39 | 34,25, | 34 | 30,16, | 22090527 |

Alignment information for chicken and turkey sequence fragments with the RIG-I gene sequences of goose, human, and zebra finch. The chicken and turkey sequences that aligned with the indicated RIG-I gene sequences appear to represent residual fragments of the RIG-I gene.

**Table S20. Carcass traits of geese after overfeeding.**

| Group | Body weight on day 90 (kg) | Body weight on day 110 (kg) | Liver weight (g) | Liver weight relative to body weight (%) |
| --- | --- | --- | --- | --- |
| Control | 3.802±0.375 | 4.414±0.400 | 143.93±40.77 | 3.26±0.25 |
| Overfed | 3.574±0.465 | 6.652±0.638 | 561.33±210.03 | 8.44±0.31 |
| *P* value | 0.117 >0.05 | 0.00 <0.01 | 0.00 <0.01 | 0.00 <0.01 |

**Table S21. Plasma parameters of geese after overfeeding.**

| Group | Plasma glucose on day 90 (mmol/L) | Plasma glucose on day 110 (mmol/L) | TC (mmol/L) | TG (mmol/L) | HDL (mmol/L) | VLDL (mmol/L) | [Lipoprotein](javascript:void(0);) (mg/L) | [Free](javascript:void(0);) [fatty](javascript:void(0);) [acid](javascript:void(0);) (mmol/L) | Phospholipid (mg/L) |
| --- | --- | --- | --- | --- | --- | --- | --- | --- | --- |
| Control | 10.69±1.52 | 11.03±1.26 | 0.94±0.04 | 1.27±0.06 | 0.63±0.05 | 0.34±0.06 | 13.96±1.50 | 82.36±5.83 | 132.48±7.00 |
| Overfed | 10.07±0.46 | 13.36±3.55 | 0.99±0.04 | 1.43±0.11 | 0.63±0.04 | 0.36±0.07 | 13.13±1.20 | 87.44±4.08 | 134.94±3.59 |
| *P* value | 0.16 | 0.02^a^ | 0.01^a^ | 0.00^b^ | 0.84 | 0.56 | 0.13 | 0.01^a^ | 0.25 |

HDL, high-density lipoprotein; TC, total cholesterol; TG, triglyceride; VLDL, low-density lipoprotein

^a^Differences between groups significant at *P* <0.05.

^b^Differences between groups significant at *P* <0.01.

**Table S22. Copy-number variation of glucolipid metabolism-related genes in geese and other animals.**

| **Gene symbol** | **Lizard** | **Goose** | **Duck** | **Chicken** | **Turkey** | **Zebra finch** | **Description** |
| --- | --- | --- | --- | --- | --- | --- | --- |
| ABCA1 | 62 | 63 | 65 | 60 | 64 | 63 | ATP-binding cassette sub-family A member 1 |
| ABCG1 | 34 | 33 | 40 | 40 | 37 | 46 | ATP-binding cassette sub-family G member 1 |
| ACACA | 8 | 9 | 7 | 7 | 6 | 7 | Acetyl-CoA carboxylase |
| ALDOB | 3 | 2 | 2 | 1 | 2 | 1 | Fructose-bisphosphate aldolase B |
| APOA1 | 4 | 1 | 3 | 8 | 4 | 1 | Apolipoprotein A-I preproprotein |
| APOB | 1 | 4 | 2 | 4 | 3 | 2 | Apolipoprotein B |
| DGAT2 | 4 | 4 | 4 | 4 | 4 | 4 | Diacylglycerol O-acyltransferase 2 |
| ELOVL1 | 6 | 8 | 8 | 7 | 5 | 7 | Elongation of very long chain fatty acids protein 1 |
| FADS1 | 8 | 6 | 4 | 6 | 4 | 8 | Fatty acid desaturase 1 |
| FADS2 | 9 | 5 | 5 | 7 | 5 | 9 | Fatty acid desaturase 2 |
| FATP1 | 12 | 6 | 9 | 7 | 9 | 7 | Long-chain fatty acid transport protein 1 |
| FN1 | 140 | 171 | 193 | 145 | 153 | 128 | Fibronectin precursor |
| HK1 | 8 | 11 | 11 | 6 | 9 | 8 | Hexokinase-1 |
| HMG-CoA | 2 | 3 | 2 | 2 | 2 | 2 | Hydroxymethylglutaryl CoA Synthase |
| LDHA | 3 | 3 | 4 | 4 | 4 | 3 | L-lactate dehydrogenase A chain |
| LDLR | 158 | 123 | 159 | 132 | 133 | 132 | Low density lipoprotein receptor |
| LEPR | 8 | 7 | 6 | 9 | 6 | 6 | Leptin receptor |
| LPL | 7 | 6 | 9 | 8 | 8 | 10 | Lipoprotein lipase |
| LXR | 41 | 25 | 31 | 33 | 24 | 36 | Liver X receptor |
| MDH1 | 2 | 2 | 2 | 2 | 2 | 3 | Malate dehydrogenase |
| PPARA | 34 | 26 | 33 | 31 | 27 | 38 | Peroxisome proliferator-activated receptor alpha |
| SREBP-1c | 8 | 4 | 6 | 4 | 6 | 8 | Sterol regulatory element-binding protein 1 |
| cs | 1 | 1 | 2 | 0 | 1 | 0 | Citrate synthase |
| gpi | 1 | 1 | 1 | 1 | 1 | 1 | Glucose-6-phosphate isomerase |
| pdh | 7 | 5 | 5 | 6 | 3 | 6 | Pyruvate dehydrogenase |
| pfkm | 5 | 6 | 7 | 4 | 5 | 8 | Phosphofructokinase |
| acly | 2 | 2 | 2 | 2 | 2 | 2 | ATP citrate lyase |
| fasn | 9 | 10 | 11 | 11 | 9 | 10 | Fatty acid synthase |
| me1 | 4 | 3 | 4 | 3 | 4 | 4 | Malic enzyme 1 |
| pksg | 2 | 1 | 2 | 2 | 2 | 2 | Hydroxymethylglutaryl-CoA synthase |
| scd | 1 | 2 | 2 | 1 | 2 | 2 | Stearoyl-CoA desaturase (delta-9 desaturase) |
| Total | 594 | 553 | 641 | 557 | 546 | 564 |  |

**Table S23. Comparison of the chicken, duck, goose, human, and mouse *lep* gene sequences.**

| **Species** | **NCBI (Lep)** | **Gene (prediction)** | **Identity** | **E value** | **Bit score** |
| --- | --- | --- | --- | --- | --- |
| Chicken | AF012727 | - | - | - | - |
| Goose | - | - | - | - | - |
| Duck | - | - | - | - | - |
| Human | BC069527.1 | ENSP00000312652 | 100.00 | 2e-94 | 340 |
| Mouse | NM_008493.3 | ENSMUSP00000067046 | 100.00 | 1e-83 | 305 |

The Accession Numbers for the chicken (AF012727), human (BC069527.1), and mouse (NM_008493.3) *lep* genes were retrieved from NCBI, along with their gene numbers. Gene predictions involved comparisons of the corresponding *lep* gene with each species (BLASTP: E value cutoff set at 1e-5) to the species’ own set of genes. For the goose *lep* gene, the *lep* genes of the other three species were used as reference genes. No significant alignments were found between chicken and goose sequences, even when the E value was set to 1e-2, whereas a comparison of the human and mouse *lef* genes produced only one result each.

**Table S24. miRNA information corresponding to glucolipid metabolism-related genes.**

| **Name** | **Target geneID** | **miR-name** | **C-1A-total-reads** | **T-1A-total-reads** | **C-1A-expressed** | **T-1A-expressed** | **C-1A-std** | **T-1A-std** | **Fold-change** | ***P* value** |
| --- | --- | --- | --- | --- | --- | --- | --- | --- | --- | --- |
| lpl | ACY_000097 | let-7a | 10396977 | 11321273 | 634 | 122 | 60.9793 | 10.7762 | -2.50047106 | 3.12E-94 |
|  | ACY_000097 | miR-3150b-3p | 10396977 | 11321273 | 0 | 20 | 0.01 | 1.7666 | 7.46483161 | 2.29E-06 |
|  | ACY_000097 | miR-4900b | 10396977 | 11321273 | 0 | 17 | 0.01 | 1.5016 | 7.23035675 | 1.62E-05 |
|  | ACY_000097 | miR-920 | 10396977 | 11321273 | 3691 | 23 | 355.007 | 2.0316 | -7.44908727 | 0 |
|  | ACY_000097 | miR-92b-3p | 10396977 | 11321273 | 2033 | 2777 | 195.5376 | 245.2904 | 0.32704472 | 6.10E-15 |
| fasn | ACY_000256 | miR-17-3p | 10396977 | 11321273 | 87 | 59 | 8.3678 | 5.2114 | -0.68317738 | 0.0046399 |
|  | ACY_000256 | miR-210 | 10396977 | 11321273 | 64 | 56 | 6.1556 | 4.9464 | -0.31552058 | 0.2315817 |
|  | ACY_000256 | miR-425 | 10396977 | 11321273 | 407 | 533 | 39.146 | 47.0795 | 0.2662341 | 0.004963 |
|  | ACY_000256 | miR-425-5p | 10396977 | 11321273 | 385 | 503 | 37.03 | 44.4296 | 0.2628266 | 0.0070245 |
|  | ACY_000256 | miR-4485 | 10396977 | 11321273 | 14 | 33 | 1.3465 | 2.9149 | 1.11423216 | 0.0130214 |
|  | ACY_000256 | miR-4608 | 10396977 | 11321273 | 12 | 14 | 1.1542 | 1.2366 | 0.09948567 | 0.8723425 |
|  | ACY_000256 | miR-92a | 10396977 | 11321273 | 2052 | 2784 | 197.3651 | 245.9087 | 0.31725588 | 3.17E-14 |
| fads2 | ACY_002170 | miR-1386 | 10396977 | 11321273 | 85 | 99 | 8.1755 | 8.7446 | 0.09708543 | 0.6528166 |
|  | ACY_002170 | miR-558 | 10396977 | 11321273 | 0 | 15 | 0.01 | 1.3249 | 7.04973966 | 5.95E-05 |
| fads1 | ACY_002171 | miR-1352 | 10396977 | 11321273 | 153558 | 141592 | 14769.485 | 12506.721 | -0.23991599 | 0 |
|  | ACY_002171 | miR-1412 | 10396977 | 11321273 | 0 | 46 | 0.01 | 4.0631 | 8.66643706 | 1.01E-13 |
|  | ACY_002171 | miR-181b | 10396977 | 11321273 | 406 | 544 | 39.0498 | 48.0511 | 0.2992543 | 0.0015134 |
|  | ACY_002171 | miR-181b-5p | 10396977 | 11321273 | 405 | 537 | 38.9536 | 47.4328 | 0.28412837 | 0.0026984 |
|  | ACY_002171 | miR-34 | 10396977 | 11321273 | 5565 | 116 | 535.2517 | 10.2462 | -5.70705661 | 0 |
|  | ACY_002171 | miR-4499 | 10396977 | 11321273 | 150 | 128 | 14.4273 | 11.3061 | -0.35169997 | 0.0424531 |
|  | ACY_002171 | miR-758-5p | 10396977 | 11321273 | 12592 | 11287 | 1211.1213 | 996.9727 | -0.28071746 | 4.94E-51 |
| pfkm | ACY_003451 | miR-215 | 10396977 | 11321273 | 199 | 253 | 19.1402 | 22.3473 | 0.22349463 | 0.1021677 |
|  | ACY_003451 | miR-301a* | 10396977 | 11321273 | 38 | 35 | 3.6549 | 3.0915 | -0.24152493 | 0.4740529 |
|  | ACY_003451 | miR-301a-5p | 10396977 | 11321273 | 40 | 37 | 3.8473 | 3.2682 | -0.23535006 | 0.4736182 |
|  | ACY_003451 | miR-4256 | 10396977 | 11321273 | 0 | 12 | 0.01 | 1.06 | 6.72792045 | 0.0004197 |
|  | ACY_003451 | miR-4699-5p | 10396977 | 11321273 | 220 | 13 | 21.16 | 1.1483 | -4.20376812 | 6.355E-54 |
|  | ACY_005198 | miR-92b | 10396977 | 11321273 | 118 | 174 | 11.3495 | 15.3693 | 0.43742272 | 0.0106493 |
|  | ACY_005198 | miR-92c | 10396977 | 11321273 | 7 | 86 | 0.6733 | 7.5963 | 3.49597551 | 3.13E-17 |
|  | ACY_005198 | miR-988* | 10396977 | 11321273 | 0 | 28 | 0.01 | 2.4732 | 7.9502351 | 1.25E-08 |
| dgat2 | ACY_005750 | miR-126* | 10396977 | 11321273 | 530 | 706 | 50.9764 | 62.3605 | 0.290803 | 0.0004352 |
|  | ACY_005750 | miR-126-5p | 10396977 | 11321273 | 539 | 712 | 51.842 | 62.8905 | 0.27872073 | 0.0006911 |
|  | ACY_005750 | miR-203-3p | 10396977 | 11321273 | 395 | 2104 | 37.9918 | 185.8448 | 2.29033835 | 3.58E-250 |
|  | ACY_005750 | miR-203a | 10396977 | 11321273 | 0 | 420 | 0.01 | 37.0983 | 11.85713736 | 1.54E-119 |
|  | ACY_005750 | miR-279d-5p | 10396977 | 11321273 | 57 | 83 | 5.4824 | 7.3313 | 0.41926145 | 0.0910563 |
|  | ACY_005750 | miR-3676-5p | 10396977 | 11321273 | 17 | 30 | 1.6351 | 2.6499 | 0.69655905 | 0.1117596 |
| gpi | ACY_006828 | miR-1587 | 10396977 | 11321273 | 1673 | 1191 | 160.9122 | 105.2002 | -0.61313626 | 1.29E-29 |
|  | ACY_006828 | miR-3182 | 10396977 | 11321273 | 4685 | 7383 | 450.6117 | 652.1351 | 0.5332861 | 4.64E-89 |
|  | ACY_006828 | miR-323a-5p | 10396977 | 11321273 | 0 | 33 | 0.01 | 2.9149 | 8.18730258 | 4.80E-10 |
|  | ACY_006828 | miR-327 | 10396977 | 11321273 | 0 | 39 | 0.01 | 3.4448 | 8.42827641 | 9.63E-12 |
|  | ACY_006828 | miR-3602 | 10396977 | 11321273 | 0 | 3609 | 0.01 | 318.7804 | 14.96027531 | 0 |
|  | ACY_006828 | miR-4020b-5p | 10396977 | 11321273 | 0 | 17 | 0.01 | 1.5016 | 7.23035675 | 1.62E-05 |
|  | ACY_006828 | miR-4206-3p | 10396977 | 11321273 | 12751 | 14607 | 1226.4142 | 1290.2259 | 0.07317738 | 2.83E-05 |
| pdh | ACY_008259 | miR-129-2-3p | 10396977 | 11321273 | 16 | 28 | 1.5389 | 2.4732 | 0.68447942 | 0.1307823 |
|  | ACY_008259 | miR-204 | 10396977 | 11321273 | 0 | 14 | 0.01 | 1.2366 | 6.9502351 | 0.000114 |
|  | ACY_008259 | miR-21 | 10396977 | 11321273 | 36615 | 43446 | 3521.6967 | 3837.5543 | 0.1239165 | 8.28E-34 |
|  | ACY_008259 | miR-222 | 10396977 | 11321273 | 31358 | 97929 | 3016.069 | 8649.999 | 1.52003053 | 0 |
|  | ACY_008259 | miR-222-3p | 10396977 | 11321273 | 31166 | 97503 | 2997.6021 | 8612.3707 | 1.52260152 | 0 |
|  | ACY_008259 | miR-31 | 10396977 | 11321273 | 50 | 77 | 4.8091 | 6.8014 | 0.50006482 | 0.0556967 |
|  | ACY_008259 | miR-3135b | 10396977 | 11321273 | 0 | 3733 | 0.01 | 329.7332 | 15.00901154 | 0 |
|  | ACY_008259 | miR-4507 | 10396977 | 11321273 | 1685 | 1266 | 162.0663 | 111.8249 | -0.53534266 | 1.06E-23 |
|  | ACY_008259 | miR-5102 | 10396977 | 11321273 | 0 | 100 | 0.01 | 8.8329 | 9.78674337 | 5.31E-29 |
|  | ACY_008259 | miR-720 | 10396977 | 11321273 | 30 | 44 | 2.8855 | 3.8865 | 0.42965019 | 0.210992 |
|  | ACY_008259 | miR-72-5p | 10396977 | 11321273 | 21 | 63 | 2.0198 | 5.5647 | 1.46209147 | 1.97E-05 |
| acc | ACY_008600 | miR-126-3p | 10396977 | 11321273 | 240 | 208 | 23.0836 | 18.3725 | -0.32932029 | 0.015818 |
|  | ACY_008600 | miR-126a | 10396977 | 11321273 | 225 | 207 | 21.6409 | 18.2842 | -0.24316299 | 0.0798627 |
|  | ACY_008600 | miR-128 | 10396977 | 11321273 | 18231 | 19879 | 1753.4905 | 1755.8979 | 0.00197934 | 0.8937941 |
|  | ACY_008600 | miR-128-3p | 10396977 | 11321273 | 19381 | 21000 | 1864.0995 | 1854.9151 | -0.00712571 | 0.6198052 |
|  | ACY_008600 | miR-1765 | 10396977 | 11321273 | 25 | 22 | 2.4045 | 1.9432 | -0.30730253 | 0.466077 |
|  | ACY_008600 | miR-221-3p | 10396977 | 11321273 | 21789 | 44747 | 2095.7053 | 3952.4707 | 0.91531891 | 0 |
|  | ACY_008600 | miR-23* | 10396977 | 11321273 | 84 | 109 | 8.0793 | 9.6279 | 0.25299086 | 0.2286267 |
|  | ACY_008600 | miR-2388 | 10396977 | 11321273 | 0 | 24 | 0.01 | 2.1199 | 7.7278524 | 1.69E-07 |
|  | ACY_008600 | miR-23a* | 10396977 | 11321273 | 11 | 21 | 1.058 | 1.8549 | 0.81000178 | 0.1321787 |
|  | ACY_008600 | miR-23b-5p | 10396977 | 11321273 | 87 | 112 | 8.3678 | 9.8929 | 0.24154512 | 0.24301 |
|  | ACY_008600 | miR-24 | 10396977 | 11321273 | 2009 | 1891 | 193.2292 | 167.0307 | -0.21019983 | 5.39E-06 |
|  | ACY_008600 | miR-24-1* | 10396977 | 11321273 | 19 | 20 | 1.8275 | 1.7666 | -0.04889599 | 0.9099074 |
|  | ACY_008600 | miR-24-1-5p | 10396977 | 11321273 | 17 | 20 | 1.6351 | 1.7666 | 0.11159655 | 0.824456 |
|  | ACY_008600 | miR-24-3p | 10396977 | 11321273 | 2002 | 1904 | 192.556 | 168.179 | -0.19528051 | 2.34E-05 |
|  | ACY_008600 | miR-24b-3p | 10396977 | 11321273 | 2156 | 2062 | 207.368 | 182.135 | -0.1871851 | 2.52E-05 |
|  | ACY_008600 | miR-2981 | 10396977 | 11321273 | 96 | 2220 | 9.2335 | 196.091 | 4.4085019 | 0 |
|  | ACY_008600 | miR-3178 | 10396977 | 11321273 | 270 | 518 | 25.9691 | 45.7546 | 0.81712078 | 1.18E-14 |
|  | ACY_008600 | miR-3540 | 10396977 | 11321273 | 71 | 58 | 6.8289 | 5.1231 | -0.41463616 | 0.1038323 |
|  | ACY_008600 | miR-3592 | 10396977 | 11321273 | 11 | 23 | 1.058 | 2.0316 | 0.94127675 | 0.0729616 |
|  | ACY_008600 | miR-4454 | 10396977 | 11321273 | 128 | 162 | 12.3113 | 14.3093 | 0.21696999 | 0.2044406 |
|  | ACY_008600 | miR-4634 | 10396977 | 11321273 | 174 | 330 | 16.7356 | 29.1487 | 0.80051127 | 1.45E-09 |
|  | ACY_008600 | miR-4707-5p | 10396977 | 11321273 | 0 | 13 | 0.01 | 1.1483 | 6.84335579 | 0.0002188 |
|  | ACY_008600 | miR-4792 | 10396977 | 11321273 | 1710 | 2310 | 164.4709 | 204.0407 | 0.31102461 | 1.15E-11 |
|  | ACY_008600 | miR-491 | 10396977 | 11321273 | 11 | 14 | 1.058 | 1.2366 | 0.22503928 | 0.7127739 |
|  | ACY_008600 | miR-5109 | 10396977 | 11321273 | 146 | 194 | 14.0425 | 17.1359 | 0.28722216 | 0.0690795 |
| me1 | ACY_010499 | miR-1013 | 10396977 | 11321273 | 515 | 508 | 49.5336 | 44.8713 | -0.14261449 | 0.1137449 |
|  | ACY_010499 | miR-1196-3p | 10396977 | 11321273 | 0 | 17 | 0.01 | 1.5016 | 7.23035675 | 1.62E-05 |
|  | ACY_010499 | miR-20a-5p | 10396977 | 11321273 | 51 | 79 | 4.9053 | 6.978 | 0.50847223 | 0.0490405 |
|  | ACY_010499 | miR-20b | 10396977 | 11321273 | 13 | 11 | 1.2504 | 0.9716 | -0.36395529 | 0.5393611 |
|  | ACY_010499 | miR-223 | 10396977 | 11321273 | 175 | 284 | 16.8318 | 25.0855 | 0.57566423 | 2.71E-05 |
|  | ACY_010499 | miR-223-3p | 10396977 | 11321273 | 123 | 207 | 11.8304 | 18.2842 | 0.62809865 | 0.0001084 |
|  | ACY_010499 | miR-256 | 10396977 | 11321273 | 0 | 88 | 0.01 | 7.773 | 9.60232771 | 1.32E-25 |
|  | ACY_010499 | miR-26-1* | 10396977 | 11321273 | 404 | 287 | 38.8574 | 25.3505 | -0.61617517 | 2.46E-08 |
|  | ACY_010499 | miR-429-3p | 10396977 | 11321273 | 41 | 37 | 3.9435 | 3.2682 | -0.27098037 | 0.4069492 |
|  | ACY_010499 | miR-4419b | 10396977 | 11321273 | 1 | 35 | 0.0962 | 3.0915 | 5.0061263 | 2.38E-09 |
| mdh1 | ACY_010783 | miR-125b-5p | 10396977 | 11321273 | 1650 | 1139 | 158.7 | 100.6071 | -0.65757001 | 6.84E-33 |
|  | ACY_010783 | miR-125c | 10396977 | 11321273 | 1527 | 1069 | 146.8696 | 94.424 | -0.6373103 | 5.33E-29 |
|  | ACY_010783 | miR-145 | 10396977 | 11321273 | 639 | 459 | 61.4602 | 40.5431 | -0.60019606 | 7.33E-12 |
|  | ACY_010783 | miR-145-5p | 10396977 | 11321273 | 655 | 472 | 62.9991 | 41.6914 | -0.5955814 | 5.64E-12 |
|  | ACY_010783 | miR-21* | 10396977 | 11321273 | 354 | 451 | 34.0484 | 39.8365 | 0.22650389 | 0.0268779 |
|  | ACY_010783 | miR-22 | 10396977 | 11321273 | 580 | 1 | 55.7854 | 0.0883 | -9.30325846 | 8.16E-184 |
|  | ACY_010783 | miR-2962 | 10396977 | 11321273 | 0 | 34 | 0.01 | 3.0032 | 8.23035675 | 2.50E-10 |
|  | ACY_010783 | miR-320 | 10396977 | 11321273 | 0 | 12 | 0.01 | 1.06 | 6.72792045 | 0.0004197 |
|  | ACY_010783 | miR-320a | 10396977 | 11321273 | 8 | 16 | 0.7695 | 1.4133 | 0.87707451 | 0.1628855 |
|  | ACY_010783 | miR-33 | 10396977 | 11321273 | 2281 | 3442 | 219.3907 | 304.0294 | 0.47070847 | 3.48E-34 |
|  | ACY_010783 | miR-33a-5p | 10396977 | 11321273 | 2236 | 3399 | 215.0625 | 300.2313 | 0.4813184 | 4.07E-35 |
|  | ACY_010783 | miR-347 | 10396977 | 11321273 | 10 | 12 | 0.9618 | 1.06 | 0.14025543 | 0.8340986 |
|  | ACY_010783 | miR-4324 | 10396977 | 11321273 | 0 | 19 | 0.01 | 1.6783 | 7.39085681 | 4.39E-06 |
|  | ACY_010783 | miR-604 | 10396977 | 11321273 | 21 | 13 | 2.0198 | 1.1483 | -0.81471284 | 0.1079113 |
| pksG | ACY_011039 | let-7-5p | 10396977 | 11321273 | 626649 | 608291 | 60272.231 | 53729.912 | -0.165768 | 0 |
|  | ACY_011039 | let-7b | 10396977 | 11321273 | 51754 | 59454 | 4977.7931 | 5251.5296 | 0.07723143 | 5.13E-19 |
|  | ACY_011039 | let-7b-5p | 10396977 | 11321273 | 45812 | 52515 | 4406.2808 | 4638.6126 | 0.07413193 | 8.87E-16 |
|  | ACY_011039 | let-7c | 10396977 | 11321273 | 20216 | 20291 | 1944.4113 | 1792.2896 | -0.11752966 | 2.47E-16 |
|  | ACY_011039 | let-7d | 10396977 | 11321273 | 601 | 710 | 57.8053 | 62.7138 | 0.11758116 | 0.1416649 |
|  | ACY_011039 | let-7e | 10396977 | 11321273 | 121 | 143 | 11.638 | 12.6311 | 0.11813713 | 0.5101325 |
|  | ACY_011039 | let-7f-5p | 10396977 | 11321273 | 1105121 | 975410 | 106292.53 | 86157.272 | -0.30299575 | 0 |
|  | ACY_011039 | let-7g | 10396977 | 11321273 | 63934 | 53028 | 6149.2874 | 4683.9256 | -0.39270108 | 0 |
|  | ACY_011039 | let-7g-5p | 10396977 | 11321273 | 64225 | 53385 | 6177.2763 | 4715.4591 | -0.38957262 | 0 |
|  | ACY_011039 | miR-10d | 10396977 | 11321273 | 14 | 40 | 1.3465 | 3.5332 | 1.39176119 | 0.0011109 |
|  | ACY_011039 | miR-122-3p | 10396977 | 11321273 | 1666 | 2354 | 160.2389 | 207.9271 | 0.37585338 | 2.73E-16 |
|  | ACY_011039 | miR-1343 | 10396977 | 11321273 | 78 | 7 | 7.5022 | 0.6183 | -3.60093481 | 6.50E-18 |
|  | ACY_011039 | miR-1357 | 10396977 | 11321273 | 123 | 131 | 11.8304 | 11.5711 | -0.03197283 | 0.8574725 |
|  | ACY_011039 | miR-139 | 10396977 | 11321273 | 119 | 150 | 11.4456 | 13.2494 | 0.21113393 | 0.2344876 |
|  | ACY_011039 | miR-139-5p | 10396977 | 11321273 | 101 | 142 | 9.7144 | 12.5428 | 0.36866265 | 0.0492649 |
|  | ACY_011039 | miR-144 | 10396977 | 11321273 | 1353 | 1154 | 130.134 | 101.932 | -0.35239091 | 1.01E-09 |
|  | ACY_011039 | miR-144-3p | 10396977 | 11321273 | 1378 | 1180 | 132.5385 | 104.2286 | -0.34666029 | 1.28E-09 |
|  | ACY_011039 | miR-146a-3p | 10396977 | 11321273 | 61 | 1857 | 5.8671 | 164.0275 | 4.80514632 | 0 |
|  | ACY_011039 | miR-146c | 10396977 | 11321273 | 3553 | 2045 | 341.7339 | 180.6334 | -0.91980869 | 1.40E-121 |
|  | ACY_011039 | miR-1579 | 10396977 | 11321273 | 1938 | 3003 | 186.4003 | 265.2529 | 0.50896434 | 2.16E-34 |
|  | ACY_011039 | miR-200b | 10396977 | 11321273 | 214 | 138 | 20.5829 | 12.1894 | -0.75581915 | 1.19E-06 |
|  | ACY_011039 | miR-200b-3p | 10396977 | 11321273 | 170 | 119 | 16.3509 | 10.5112 | -0.63744267 | 0.0001945 |
|  | ACY_011039 | miR-2013 | 10396977 | 11321273 | 6 | 17 | 0.5771 | 1.5016 | 1.37960732 | 0.0382114 |
|  | ACY_011039 | miR-203 | 10396977 | 11321273 | 376 | 2046 | 36.1644 | 180.7217 | 2.32112763 | 2.89E-247 |
|  | ACY_011039 | miR-223-5p | 10396977 | 11321273 | 34 | 50 | 3.2702 | 4.4165 | 0.43352464 | 0.178105 |
|  | ACY_011039 | miR-2305 | 10396977 | 11321273 | 49 | 4 | 4.7129 | 0.3533 | -3.73764941 | 5.07E-12 |
|  | ACY_011039 | miR-2476 | 10396977 | 11321273 | 19950 | 25562 | 1918.827 | 2257.8733 | 0.23473989 | 7.75E-67 |
|  | ACY_011039 | miR-26 | 10396977 | 11321273 | 12499 | 10414 | 1202.1764 | 919.8612 | -0.38616051 | 5.76E-91 |
|  | ACY_011039 | miR-26a | 10396977 | 11321273 | 12397 | 10202 | 1192.3658 | 901.1354 | -0.4040111 | 5.26E-98 |
|  | ACY_011039 | miR-26a-5p | 10396977 | 11321273 | 10555 | 8802 | 1015.1989 | 777.4744 | -0.38489533 | 1.18E-76 |
|  | ACY_011039 | miR-26b | 10396977 | 11321273 | 35 | 31 | 3.3664 | 2.7382 | -0.29797878 | 0.4020366 |
|  | ACY_011039 | miR-26b-5p | 10396977 | 11321273 | 33 | 31 | 3.174 | 2.7382 | -0.2130743 | 0.5536026 |
|  | ACY_011039 | miR-27a | 10396977 | 11321273 | 1205 | 1573 | 115.8991 | 138.942 | 0.26161341 | 2.03E-06 |
|  | ACY_011039 | miR-27b | 10396977 | 11321273 | 1221 | 1588 | 117.438 | 140.2669 | 0.2562753 | 2.87E-06 |
|  | ACY_011039 | miR-2820 | 10396977 | 11321273 | 0 | 20 | 0.01 | 1.7666 | 7.46483161 | 2.29E-06 |
|  | ACY_011039 | miR-2986 | 10396977 | 11321273 | 8 | 14 | 0.7695 | 1.2366 | 0.68438568 | 0.2944652 |
|  | ACY_011039 | miR-352 | 10396977 | 11321273 | 29 | 1 | 2.7893 | 0.0883 | -4.98134586 | 8.41E-09 |
|  | ACY_011039 | miR-3594-5p | 10396977 | 11321273 | 0 | 15 | 0.01 | 1.3249 | 7.04973966 | 5.95E-05 |
|  | ACY_011039 | miR-429 | 10396977 | 11321273 | 40 | 36 | 3.8473 | 3.1799 | -0.27486493 | 0.4063914 |
|  | ACY_011039 | miR-4508 | 10396977 | 11321273 | 5685 | 10387 | 546.7936 | 917.4763 | 0.74667453 | 8.46E-225 |
|  | ACY_011039 | miR-455* | 10396977 | 11321273 | 28 | 19 | 2.6931 | 1.6783 | -0.68226718 | 0.1103978 |
|  | ACY_011039 | miR-4618 | 10396977 | 11321273 | 0 | 44 | 0.01 | 3.8865 | 8.60232771 | 3.71E-13 |
|  | ACY_011039 | miR-494 | 10396977 | 11321273 | 726 | 882 | 69.828 | 77.9064 | 0.1579362 | 0.0288375 |
|  | ACY_011039 | miR-5093 | 10396977 | 11321273 | 9 | 31 | 0.8656 | 2.7382 | 1.66145542 | 0.001139 |
|  | ACY_011039 | miR-696 | 10396977 | 11321273 | 278 | 326 | 26.7385 | 28.7953 | 0.10691482 | 0.365269 |
|  | ACY_011039 | miR-716b | 10396977 | 11321273 | 2997 | 2912 | 288.2569 | 257.2149 | -0.16438092 | 1.19E-05 |
|  | ACY_011039 | miR-87a-3p | 10396977 | 11321273 | 110 | 97 | 10.58 | 8.5679 | -0.30432608 | 0.1295047 |
| acly | ACY_011179 | lin-4 | 10396977 | 11321273 | 0 | 25 | 0.01 | 2.2082 | 7.78672703 | 8.81E-08 |
|  | ACY_011179 | miR-101b-3p | 10396977 | 11321273 | 48 | 24 | 4.6167 | 2.1199 | -1.12286578 | 0.0014028 |
|  | ACY_011179 | miR-103a-3p | 10396977 | 11321273 | 33481 | 40821 | 3220.263 | 3605.6899 | 0.16309681 | 3.20E-53 |
|  | ACY_011179 | miR-10a | 10396977 | 11321273 | 5007 | 3911 | 481.5823 | 345.4559 | -0.47928082 | 4.04E-55 |
|  | ACY_011179 | miR-10a-5p | 10396977 | 11321273 | 4870 | 3823 | 468.4054 | 337.6829 | -0.47208858 | 3.06E-52 |
|  | ACY_011179 | miR-1207-5p | 10396977 | 11321273 | 6 | 25 | 0.5771 | 2.2082 | 1.93597761 | 0.0012459 |
|  | ACY_011179 | miR-125b-2-3p | 10396977 | 11321273 | 20 | 14 | 1.9236 | 1.2366 | -0.63742992 | 0.2050123 |
|  | ACY_011179 | miR-1410 | 10396977 | 11321273 | 13 | 37 | 1.2504 | 3.2682 | 1.38610659 | 0.0017892 |
|  | ACY_011179 | miR-1570 | 10396977 | 11321273 | 0 | 12 | 0.01 | 1.06 | 6.72792045 | 0.0004197 |
|  | ACY_011179 | miR-17 | 10396977 | 11321273 | 123 | 10 | 11.8304 | 0.8833 | -3.74345153 | 2.49E-28 |
|  | ACY_011179 | miR-18 | 10396977 | 11321273 | 6 | 14 | 0.5771 | 1.2366 | 1.09948567 | 0.1174559 |
|  | ACY_011179 | miR-181a | 10396977 | 11321273 | 1216 | 1330 | 116.9571 | 117.478 | 0.00641117 | 0.9117348 |
|  | ACY_011179 | miR-181a-5p | 10396977 | 11321273 | 1163 | 1309 | 111.8594 | 115.623 | 0.04774191 | 0.4121392 |
|  | ACY_011179 | miR-18a | 10396977 | 11321273 | 11 | 11 | 1.058 | 0.9716 | -0.12290523 | 0.8364573 |
|  | ACY_011179 | miR-1907 | 10396977 | 11321273 | 91 | 59 | 8.7525 | 5.2114 | -0.74802416 | 0.0017182 |
|  | ACY_011179 | miR-199* | 10396977 | 11321273 | 43402 | 37617 | 4174.4826 | 3322.6829 | -0.32924878 | 5.45E-231 |
|  | ACY_011179 | miR-199-3p | 10396977 | 11321273 | 339 | 356 | 32.6056 | 31.4452 | -0.05227996 | 0.6318024 |
|  | ACY_011179 | miR-217 | 10396977 | 11321273 | 1679 | 251 | 161.4892 | 22.1707 | -2.86471146 | 2.12E-286 |
|  | ACY_011179 | miR-25 | 10396977 | 11321273 | 163 | 163 | 15.6776 | 14.3977 | -0.12286636 | 0.4409861 |
|  | ACY_011179 | miR-2976 | 10396977 | 11321273 | 4 | 17 | 0.3847 | 1.5016 | 1.96469482 | 0.0078869 |
|  | ACY_011179 | miR-301b-5p | 10396977 | 11321273 | 26 | 40 | 2.5007 | 3.5332 | 0.49864342 | 0.1718711 |
|  | ACY_011179 | miR-3141 | 10396977 | 11321273 | 39 | 36 | 3.7511 | 3.1799 | -0.23833233 | 0.4738708 |
|  | ACY_011179 | miR-3535 | 10396977 | 11321273 | 19 | 6 | 1.8275 | 0.53 | -1.78580714 | 0.0048992 |
|  | ACY_011179 | miR-3539 | 10396977 | 11321273 | 32 | 38 | 3.0778 | 3.3565 | 0.12505816 | 0.7248927 |
|  | ACY_011179 | miR-3662 | 10396977 | 11321273 | 18 | 4 | 1.7313 | 0.3533 | -2.29289008 | 0.0013207 |
|  | ACY_011179 | miR-3665 | 10396977 | 11321273 | 33 | 150 | 3.174 | 13.2494 | 2.061553 | 4.08E-17 |
|  | ACY_011179 | miR-3886-5p | 10396977 | 11321273 | 1522 | 1059 | 146.3887 | 93.5407 | -0.64613806 | 1.38E-29 |
|  | ACY_011179 | miR-4069-3p | 10396977 | 11321273 | 6179 | 7054 | 594.3074 | 623.0748 | 0.06819602 | 0.0066563 |
|  | ACY_011179 | miR-4110-5p | 10396977 | 11321273 | 5652 | 5017 | 543.6196 | 443.148 | -0.29480887 | 5.24E-26 |
|  | ACY_011179 | miR-4205-5p | 10396977 | 11321273 | 1934 | 1978 | 186.0156 | 174.7153 | -0.09041766 | 0.0499887 |
|  | ACY_011179 | miR-4492 | 10396977 | 11321273 | 2334 | 4466 | 224.4883 | 394.4786 | 0.81330678 | 4.99E-113 |
|  | ACY_011179 | miR-455b | 10396977 | 11321273 | 78 | 78 | 7.5022 | 6.8897 | -0.12287256 | 0.5931005 |
|  | ACY_011179 | miR-458 | 10396977 | 11321273 | 62 | 29 | 5.9633 | 2.5615 | -1.21912203 | 0.0001038 |
|  | ACY_011179 | miR-4770 | 10396977 | 11321273 | 0 | 19 | 0.01 | 1.6783 | 7.39085681 | 4.39E-06 |
|  | ACY_011179 | miR-4791 | 10396977 | 11321273 | 22 | 6 | 2.116 | 0.53 | -1.99727536 | 0.0010683 |
|  | ACY_011179 | miR-4865 | 10396977 | 11321273 | 0 | 5622 | 0.01 | 496.5873 | 15.59975975 | 0 |
|  | ACY_011179 | miR-596 | 10396977 | 11321273 | 492 | 625 | 47.3214 | 55.2058 | 0.22232709 | 0.0104545 |
|  | ACY_011179 | miR-638 | 10396977 | 11321273 | 0 | 214 | 0.01 | 18.9025 | 10.88436134 | 2.96E-61 |
|  | ACY_011179 | miR-92a-2-5p | 10396977 | 11321273 | 61 | 10 | 5.8671 | 0.8833 | -2.73167217 | 2.71E-11 |
| fatp | ACY_011453 | let-7 | 10396977 | 11321273 | 76 | 88 | 7.3098 | 7.773 | 0.08863958 | 0.6989297 |
|  | ACY_011453 | let-7d-5p | 10396977 | 11321273 | 3973 | 4347 | 382.1303 | 383.9674 | 0.00691917 | 0.8275211 |
|  | ACY_011453 | let-7g* | 10396977 | 11321273 | 0 | 41 | 0.01 | 3.6215 | 8.50044357 | 2.62E-12 |
|  | ACY_011453 | miR-1261 | 10396977 | 11321273 | 91 | 123 | 8.7525 | 10.8645 | 0.31185472 | 0.1182796 |
|  | ACY_011453 | miR-132* | 10396977 | 11321273 | 11 | 16 | 1.058 | 1.4133 | 0.41772811 | 0.4728264 |
|  | ACY_011453 | miR-132-5p | 10396977 | 11321273 | 10 | 16 | 0.9618 | 1.4133 | 0.55525891 | 0.3505508 |
|  | ACY_011453 | miR-1349 | 10396977 | 11321273 | 6 | 16 | 0.5771 | 1.4133 | 1.2921745 | 0.0562373 |
|  | ACY_011453 | miR-1451 | 10396977 | 11321273 | 62 | 71 | 5.9633 | 6.2714 | 0.07267662 | 0.7765687 |
|  | ACY_011453 | miR-146b | 10396977 | 11321273 | 19702 | 23664 | 1894.9739 | 2090.2243 | 0.14147979 | 2.41E-24 |
|  | ACY_011453 | miR-146b-5p | 10396977 | 11321273 | 265 | 306 | 25.4882 | 27.0288 | 0.08466797 | 0.485979 |
|  | ACY_011453 | miR-1599 | 10396977 | 11321273 | 47 | 85 | 4.5205 | 7.508 | 0.7319463 | 0.0046848 |
|  | ACY_011453 | miR-1711 | 10396977 | 11321273 | 0 | 12 | 0.01 | 1.06 | 6.72792045 | 0.0004197 |
|  | ACY_011453 | miR-1845 | 10396977 | 11321273 | 591 | 760 | 56.8434 | 67.1303 | 0.23997124 | 0.0023751 |
|  | ACY_011453 | miR-1961 | 10396977 | 11321273 | 105 | 22 | 10.0991 | 1.9432 | -2.37772043 | 5.38E-16 |
|  | ACY_011453 | miR-214-3p | 10396977 | 11321273 | 294 | 251 | 28.2775 | 22.1707 | -0.35100026 | 0.004568 |
|  | ACY_011453 | miR-21-5p | 10396977 | 11321273 | 38130 | 44731 | 3667.4122 | 3951.0574 | 0.10747638 | 1.05E-26 |
|  | ACY_011453 | miR-2288 | 10396977 | 11321273 | 96 | 34 | 9.2335 | 3.0032 | -1.62037706 | 1.94E-09 |
|  | ACY_011453 | miR-2315 | 10396977 | 11321273 | 0 | 71 | 0.01 | 6.2714 | 9.29264373 | 8.51E-21 |
|  | ACY_011453 | miR-2354 | 10396977 | 11321273 | 109 | 7 | 10.4838 | 0.6183 | -4.08371092 | 7.09E-27 |
|  | ACY_011453 | miR-279c* | 10396977 | 11321273 | 0 | 4190 | 0.01 | 370.0997 | 15.17562635 | 0 |
|  | ACY_011453 | miR-27b-5p | 10396977 | 11321273 | 134 | 205 | 12.8884 | 18.1075 | 0.4905142 | 0.0020591 |
|  | ACY_011453 | miR-2886 | 10396977 | 11321273 | 20 | 102 | 1.9236 | 9.0096 | 2.22765422 | 3.66E-13 |
|  | ACY_011453 | miR-2966 | 10396977 | 11321273 | 304 | 509 | 29.2393 | 44.9596 | 0.62072043 | 1.79E-09 |
|  | ACY_011453 | miR-2974 | 10396977 | 11321273 | 0 | 35 | 0.01 | 3.0915 | 8.2721632 | 1.30E-10 |
|  | ACY_011453 | miR-2982 | 10396977 | 11321273 | 0 | 12 | 0.01 | 1.06 | 6.72792045 | 0.0004197 |
|  | ACY_011453 | miR-320d | 10396977 | 11321273 | 23 | 4 | 2.2122 | 0.3533 | -2.64651617 | 7.38E-05 |
|  | ACY_011453 | miR-338-3p | 10396977 | 11321273 | 11 | 11 | 1.058 | 0.9716 | -0.12290523 | 0.8364573 |
|  | ACY_011453 | miR-3488 | 10396977 | 11321273 | 4842 | 6169 | 465.7123 | 544.9034 | 0.22656151 | 2.40E-16 |
|  | ACY_011453 | miR-3584-3p | 10396977 | 11321273 | 0 | 4839 | 0.01 | 427.4254 | 15.38338502 | 0 |
|  | ACY_011453 | miR-363* | 10396977 | 11321273 | 30 | 26 | 2.8855 | 2.2966 | -0.32932173 | 0.3941849 |
|  | ACY_011453 | miR-3652 | 10396977 | 11321273 | 140 | 2462 | 13.4655 | 217.4667 | 4.0134548 | 0 |
|  | ACY_011453 | miR-3856-5p | 10396977 | 11321273 | 0 | 702 | 0.01 | 62.0072 | 12.59822003 | 2.52E-199 |
|  | ACY_011453 | miR-3962 | 10396977 | 11321273 | 18 | 1 | 1.7313 | 0.0883 | -4.29329849 | 1.82E-05 |
|  | ACY_011453 | miR-4006d-5p | 10396977 | 11321273 | 10138 | 6795 | 975.0911 | 600.1975 | -0.7000997 | 4.59E-215 |
|  | ACY_011453 | miR-4006f-5p | 10396977 | 11321273 | 0 | 6975 | 0.01 | 616.0968 | 15.91086942 | 0 |
|  | ACY_011453 | miR-4048-3p | 10396977 | 11321273 | 0 | 41 | 0.01 | 3.6215 | 8.50044357 | 2.62E-12 |
|  | ACY_011453 | miR-4154-3p | 10396977 | 11321273 | 8103 | 8463 | 779.3612 | 747.5308 | -0.06015909 | 0.0073023 |
|  | ACY_011453 | miR-4466 | 10396977 | 11321273 | 1160 | 2331 | 111.5709 | 205.8956 | 0.88395221 | 1.00E-68 |
|  | ACY_011453 | miR-4489 | 10396977 | 11321273 | 0 | 19 | 0.01 | 1.6783 | 7.39085681 | 4.39E-06 |
|  | ACY_011453 | miR-454-3p | 10396977 | 11321273 | 36 | 56 | 3.4625 | 4.9464 | 0.51456484 | 0.0948554 |
|  | ACY_011453 | miR-454b | 10396977 | 11321273 | 40 | 58 | 3.8473 | 5.1231 | 0.41317072 | 0.1647456 |
|  | ACY_011453 | miR-462 | 10396977 | 11321273 | 14708 | 16816 | 1414.642 | 1485.3453 | 0.07036136 | 1.55E-05 |
|  | ACY_011453 | miR-4800-5p | 10396977 | 11321273 | 0 | 833 | 0.01 | 73.5783 | 12.84506463 | 2.18E-236 |
|  | ACY_011453 | miR-5105 | 10396977 | 11321273 | 275 | 312 | 26.45 | 27.5587 | 0.05924011 | 0.6214352 |
|  | ACY_011453 | miR-548an | 10396977 | 11321273 | 2 | 28 | 0.1924 | 2.4732 | 3.68419821 | 1.43E-06 |
|  | ACY_011453 | miR-5595-3p | 10396977 | 11321273 | 22 | 13 | 2.116 | 1.1483 | -0.88184002 | 0.0783303 |
| scd | ACY_012322 | miR-101c | 10396977 | 11321273 | 17080 | 12225 | 1642.7852 | 1079.8256 | -0.60534553 | 3.66E-279 |
|  | ACY_012322 | miR-1268a | 10396977 | 11321273 | 3881 | 4909 | 373.2816 | 433.6085 | 0.21612864 | 2.74E-12 |
|  | ACY_012322 | miR-130b* | 10396977 | 11321273 | 20 | 11 | 1.9236 | 0.9716 | -0.98537444 | 0.0659523 |
|  | ACY_012322 | miR-1335 | 10396977 | 11321273 | 253 | 812 | 24.334 | 71.7234 | 1.55947039 | 4.71E-59 |
|  | ACY_012322 | miR-1388* | 10396977 | 11321273 | 232 | 176 | 22.3142 | 15.546 | -0.52141866 | 0.0002796 |
|  | ACY_012322 | miR-1421g | 10396977 | 11321273 | 0 | 6303 | 0.01 | 556.7395 | 15.76471482 | 0 |
|  | ACY_012322 | miR-142-3p | 10396977 | 11321273 | 24 | 31 | 2.3084 | 2.7382 | 0.24633459 | 0.5380845 |
|  | ACY_012322 | miR-144* | 10396977 | 11321273 | 162 | 232 | 15.5815 | 20.4924 | 0.39525483 | 0.0072169 |
|  | ACY_012322 | miR-146 | 10396977 | 11321273 | 61 | 10 | 5.8671 | 0.8833 | -2.73167217 | 2.71E-11 |
|  | ACY_012322 | miR-146b* | 10396977 | 11321273 | 12 | 20 | 1.1542 | 1.7666 | 0.61408218 | 0.2498041 |
|  | ACY_012322 | miR-1696 | 10396977 | 11321273 | 103 | 59 | 9.9067 | 5.2114 | -0.92673357 | 6.13E-05 |
|  | ACY_012322 | miR-1777a | 10396977 | 11321273 | 22 | 48 | 2.116 | 4.2398 | 1.00265658 | 0.0057333 |
|  | ACY_012322 | miR-194-5p | 10396977 | 11321273 | 0 | 14 | 0.01 | 1.2366 | 6.9502351 | 0.000114 |
|  | ACY_012322 | miR-199a-5p | 10396977 | 11321273 | 992 | 1172 | 95.4123 | 103.5219 | 0.11768883 | 0.0586287 |
|  | ACY_012322 | miR-214 | 10396977 | 11321273 | 230 | 191 | 22.1218 | 16.8709 | -0.39093184 | 0.0055319 |
|  | ACY_012322 | miR-22-5p | 10396977 | 11321273 | 80 | 83 | 7.6945 | 7.3313 | -0.06975854 | 0.7551195 |
|  | ACY_012322 | miR-2374 | 10396977 | 11321273 | 14 | 38 | 1.3465 | 3.3565 | 1.31774341 | 0.0023141 |
|  | ACY_012322 | miR-2955 | 10396977 | 11321273 | 363 | 6 | 34.914 | 0.53 | -6.04166948 | 4.94E-106 |
|  | ACY_012322 | miR-2970 | 10396977 | 11321273 | 1176 | 1164 | 113.1098 | 102.8153 | -0.13766896 | 0.0209803 |
|  | ACY_012322 | miR-2989 | 10396977 | 11321273 | 86 | 59 | 8.2716 | 5.2114 | -0.66649543 | 0.005889 |
|  | ACY_012322 | miR-30c-1-3p | 10396977 | 11321273 | 1790 | 1968 | 172.1654 | 173.832 | 0.01389845 | 0.7687543 |
|  | ACY_012322 | miR-3128 | 10396977 | 11321273 | 302 | 190 | 29.0469 | 16.7826 | -0.79141796 | 1.88E-09 |
|  | ACY_012322 | miR-341 | 10396977 | 11321273 | 33 | 38 | 3.174 | 3.3565 | 0.08065551 | 0.8208214 |
|  | ACY_012322 | miR-3526 | 10396977 | 11321273 | 0 | 14 | 0.01 | 1.2366 | 6.9502351 | 0.000114 |
|  | ACY_012322 | miR-3612 | 10396977 | 11321273 | 15 | 58 | 1.4427 | 5.1231 | 1.82824572 | 1.60E-06 |
|  | ACY_012322 | miR-363-5p | 10396977 | 11321273 | 25 | 52 | 2.4045 | 4.5931 | 0.93373127 | 0.0066731 |
|  | ACY_012322 | miR-3875-5p | 10396977 | 11321273 | 0 | 23 | 0.01 | 2.0316 | 7.66647257 | 3.24E-07 |
|  | ACY_012322 | miR-456 | 10396977 | 11321273 | 2060 | 2443 | 198.1345 | 215.7885 | 0.12313787 | 0.0043011 |
|  | ACY_012322 | miR-5133 | 10396977 | 11321273 | 15 | 34 | 1.4427 | 3.0032 | 1.05772922 | 0.0156087 |
|  | ACY_012322 | miR-726 | 10396977 | 11321273 | 311 | 291 | 29.9125 | 25.7038 | -0.21876683 | 0.062825 |
|  | ACY_012322 | miR-877-5p | 10396977 | 11321273 | 2 | 34 | 0.1924 | 3.0032 | 3.96431985 | 4.06E-08 |
| hk1 | ACY_012418 | let-7a-1-3p | 10396977 | 11321273 | 13 | 13 | 1.2504 | 1.1483 | -0.12289008 | 0.8233032 |
|  | ACY_012418 | let-7a-3p | 10396977 | 11321273 | 17 | 17 | 1.6351 | 1.5016 | -0.12287832 | 0.7997279 |
|  | ACY_012418 | let-7e-5p | 10396977 | 11321273 | 77 | 133 | 7.406 | 11.7478 | 0.66562416 | 0.0011065 |
|  | ACY_012418 | let-7f-1* | 10396977 | 11321273 | 1 | 14 | 0.0962 | 1.2366 | 3.68419821 | 0.000933 |
|  | ACY_012418 | let-7i | 10396977 | 11321273 | 43273 | 49199 | 4162.0752 | 4345.7127 | 0.06228977 | 5.61E-11 |
|  | ACY_012418 | let-7i-5p | 10396977 | 11321273 | 41844 | 47695 | 4024.6314 | 4212.8655 | 0.0659452 | 8.66E-12 |
|  | ACY_012418 | miR-101 | 10396977 | 11321273 | 46381 | 40985 | 4461.0082 | 3620.1759 | -0.30131 | 6.46E-209 |
|  | ACY_012418 | miR-1013-3p | 10396977 | 11321273 | 53 | 58 | 5.0976 | 5.1231 | 0.00719888 | 0.9834724 |
|  | ACY_012418 | miR-101a | 10396977 | 11321273 | 45101 | 40206 | 4337.8955 | 3551.3674 | -0.28862068 | 1.99E-187 |
|  | ACY_012418 | miR-101a-3p | 10396977 | 11321273 | 45926 | 40842 | 4417.2455 | 3607.5448 | -0.29212971 | 3.34E-195 |
|  | ACY_012418 | miR-103 | 10396977 | 11321273 | 35692 | 42985 | 3432.9209 | 3796.8345 | 0.1453605 | 4.46E-45 |
|  | ACY_012418 | miR-107 | 10396977 | 11321273 | 56741 | 77374 | 5457.4517 | 6834.3904 | 0.3245852 | 0 |
|  | ACY_012418 | miR-107b | 10396977 | 11321273 | 5834 | 10389 | 561.1246 | 917.653 | 0.70962756 | 6.40E-206 |
|  | ACY_012418 | miR-1268 | 10396977 | 11321273 | 326 | 589 | 31.3553 | 52.026 | 0.73052347 | 7.90E-14 |
|  | ACY_012418 | miR-1273f | 10396977 | 11321273 | 4 | 32 | 0.3847 | 2.8265 | 2.87721096 | 3.65E-06 |
|  | ACY_012418 | miR-129b* | 10396977 | 11321273 | 16 | 30 | 1.5389 | 2.6499 | 0.78403843 | 0.0779102 |
|  | ACY_012418 | miR-1329 | 10396977 | 11321273 | 43 | 100 | 4.1358 | 8.8329 | 1.09472076 | 1.64E-05 |
|  | ACY_012418 | miR-135a | 10396977 | 11321273 | 2 | 14 | 0.1924 | 1.2366 | 2.68419821 | 0.0040694 |
|  | ACY_012418 | miR-135a-5p | 10396977 | 11321273 | 9 | 14 | 0.8656 | 1.2366 | 0.51460651 | 0.4175346 |
|  | ACY_012418 | miR-135b | 10396977 | 11321273 | 1 | 14 | 0.0962 | 1.2366 | 3.68419821 | 0.000933 |
|  | ACY_012418 | miR-1388 | 10396977 | 11321273 | 176 | 203 | 16.928 | 17.9308 | 0.08302832 | 0.5786431 |
|  | ACY_012418 | miR-1388-3p | 10396977 | 11321273 | 194 | 249 | 18.6593 | 21.994 | 0.23721514 | 0.085994 |
|  | ACY_012418 | miR-143 | 10396977 | 11321273 | 17090 | 15590 | 1643.747 | 1377.0536 | -0.25540355 | 1.36E-57 |
|  | ACY_012418 | miR-143-3p | 10396977 | 11321273 | 16628 | 14729 | 1599.311 | 1301.0021 | -0.29782722 | 1.54E-74 |
|  | ACY_012418 | miR-1434 | 10396977 | 11321273 | 10 | 40 | 0.9618 | 3.5332 | 1.87716659 | 5.41E-05 |
|  | ACY_012418 | miR-145-3p | 10396977 | 11321273 | 123 | 87 | 11.8304 | 7.6846 | -0.62245678 | 0.0019254 |
|  | ACY_012418 | miR-146a | 10396977 | 11321273 | 1563 | 749 | 150.3322 | 66.1586 | -1.18415345 | 1.26E-81 |
|  | ACY_012418 | miR-146a-5p | 10396977 | 11321273 | 1611 | 779 | 154.9489 | 68.8085 | -1.17113381 | 1.23E-82 |
|  | ACY_012418 | miR-1481 | 10396977 | 11321273 | 0 | 46 | 0.01 | 4.0631 | 8.66643706 | 1.01E-13 |
|  | ACY_012418 | miR-150 | 10396977 | 11321273 | 156 | 123 | 15.0044 | 10.8645 | -0.46576385 | 0.0072227 |
|  | ACY_012418 | miR-150-5p | 10396977 | 11321273 | 179 | 134 | 17.2165 | 11.8361 | -0.54059809 | 0.000976 |
|  | ACY_012418 | miR-1559 | 10396977 | 11321273 | 189 | 220 | 18.1784 | 19.4324 | 0.09623887 | 0.5032524 |
|  | ACY_012418 | miR-1607 | 10396977 | 11321273 | 27 | 58 | 2.5969 | 5.1231 | 0.98022659 | 0.0028233 |
|  | ACY_012418 | miR-1648 | 10396977 | 11321273 | 58 | 9 | 5.5785 | 0.795 | -2.81085048 | 4.32E-11 |
|  | ACY_012418 | miR-1662 | 10396977 | 11321273 | 106 | 73 | 10.1953 | 6.448 | -0.66098058 | 0.0023951 |
|  | ACY_012418 | miR-1692 | 10396977 | 11321273 | 1387 | 796 | 133.4042 | 70.3101 | -0.92400024 | 6.02E-49 |
|  | ACY_012418 | miR-1763 | 10396977 | 11321273 | 14 | 21 | 1.3465 | 1.8549 | 0.46212718 | 0.3621419 |
|  | ACY_012418 | miR-191* | 10396977 | 11321273 | 33 | 62 | 3.174 | 5.4764 | 0.7869257 | 0.0102925 |
|  | ACY_012418 | miR-193* | 10396977 | 11321273 | 361 | 776 | 34.7216 | 68.5435 | 0.98118643 | 2.86E-28 |
|  | ACY_012418 | miR-193a-5p | 10396977 | 11321273 | 321 | 719 | 30.8744 | 63.5088 | 1.04054541 | 8.14E-29 |
|  | ACY_012418 | miR-199 | 10396977 | 11321273 | 45178 | 39242 | 4345.3015 | 3466.218 | -0.32609389 | 4.60E-236 |
|  | ACY_012418 | miR-199a* | 10396977 | 11321273 | 32860 | 29497 | 3160.5341 | 2605.4491 | -0.27863631 | 2.36E-128 |
|  | ACY_012418 | miR-199a-3p | 10396977 | 11321273 | 39353 | 35234 | 3785.0425 | 3112.1942 | -0.28237741 | 3.28E-157 |
|  | ACY_012418 | miR-199b | 10396977 | 11321273 | 37568 | 32731 | 3613.358 | 2891.106 | -0.32171869 | 9.43E-192 |
|  | ACY_012418 | miR-199b-3p | 10396977 | 11321273 | 15 | 15 | 1.4427 | 1.3249 | -0.12288786 | 0.8111191 |
|  | ACY_012418 | miR-204-5p | 10396977 | 11321273 | 3 | 13 | 0.2885 | 1.1483 | 1.99285638 | 0.0203913 |
|  | ACY_012418 | miR-20b* | 10396977 | 11321273 | 20 | 27 | 1.9236 | 2.3849 | 0.31011994 | 0.4749198 |
|  | ACY_012418 | miR-20b-3p | 10396977 | 11321273 | 7 | 12 | 0.6733 | 1.06 | 0.65474289 | 0.3537898 |
|  | ACY_012418 | miR-2184 | 10396977 | 11321273 | 71 | 106 | 6.8289 | 9.3629 | 0.45530224 | 0.0390111 |
|  | ACY_012418 | miR-2188 | 10396977 | 11321273 | 594 | 663 | 57.132 | 58.5623 | 0.03567318 | 0.6628587 |
|  | ACY_012418 | miR-219-2* | 10396977 | 11321273 | 0 | 13 | 0.01 | 1.1483 | 6.84335579 | 0.0002188 |
|  | ACY_012418 | miR-222a* | 10396977 | 11321273 | 28 | 44 | 2.6931 | 3.8865 | 0.52920372 | 0.129823 |
|  | ACY_012418 | miR-2325b | 10396977 | 11321273 | 10 | 61 | 0.9618 | 5.3881 | 2.4859678 | 2.34E-09 |
|  | ACY_012418 | miR-2426 | 10396977 | 11321273 | 8 | 5671 | 0.7695 | 500.9154 | 9.34642992 | 0 |
|  | ACY_012418 | miR-2428 | 10396977 | 11321273 | 8 | 26 | 0.7695 | 2.2966 | 1.57750637 | 0.0042156 |
|  | ACY_012418 | miR-2526 | 10396977 | 11321273 | 395 | 337 | 37.9918 | 29.767 | -0.35197424 | 0.0009807 |
|  | ACY_012418 | miR-2-5p | 10396977 | 11321273 | 0 | 56 | 0.01 | 4.9464 | 8.9502351 | 1.49E-16 |
|  | ACY_012418 | miR-2812 | 10396977 | 11321273 | 147 | 166 | 14.1387 | 14.6627 | 0.05250131 | 0.7508003 |
|  | ACY_012418 | miR-2885 | 10396977 | 11321273 | 390 | 663 | 37.5109 | 58.5623 | 0.64266234 | 1.41E-12 |
|  | ACY_012418 | miR-2954 | 10396977 | 11321273 | 972 | 407 | 93.4887 | 35.95 | -1.37880023 | 8.80E-65 |
|  | ACY_012418 | miR-2964 | 10396977 | 11321273 | 134 | 145 | 12.8884 | 12.8077 | -0.00906175 | 0.9556248 |
|  | ACY_012418 | miR-2993 | 10396977 | 11321273 | 55 | 9 | 5.29 | 0.795 | -2.73424096 | 2.57E-10 |
|  | ACY_012418 | miR-30b-3p | 10396977 | 11321273 | 526 | 520 | 50.5916 | 45.9312 | -0.13942339 | 0.1179412 |
|  | ACY_012418 | miR-30c* | 10396977 | 11321273 | 44 | 144 | 4.232 | 12.7194 | 1.58761908 | 5.58E-12 |
|  | ACY_012418 | miR-30c-2-3p | 10396977 | 11321273 | 2447 | 1995 | 235.3569 | 176.2169 | -0.41749786 | 6.35E-22 |
|  | ACY_012418 | miR-30d-3p | 10396977 | 11321273 | 16 | 2 | 1.5389 | 0.1767 | -3.12252554 | 0.0003747 |
|  | ACY_012418 | miR-30e-3p | 10396977 | 11321273 | 122 | 147 | 11.7342 | 12.9844 | 0.14605986 | 0.4107773 |
|  | ACY_012418 | miR-3120-3p | 10396977 | 11321273 | 10 | 17 | 0.9618 | 1.5016 | 0.64269172 | 0.2714313 |
|  | ACY_012418 | miR-33-5p | 10396977 | 11321273 | 2060 | 2738 | 198.1345 | 241.8456 | 0.28760618 | 6.88E-12 |
|  | ACY_012418 | miR-33a | 10396977 | 11321273 | 2378 | 3560 | 228.7203 | 314.4523 | 0.45925677 | 8.20E-34 |
|  | ACY_012418 | miR-346-3p | 10396977 | 11321273 | 75 | 1 | 7.2136 | 0.0883 | -6.35216218 | 3.94E-23 |
|  | ACY_012418 | miR-3615 | 10396977 | 11321273 | 0 | 2515 | 0.01 | 222.1482 | 14.43923483 | 0 |
|  | ACY_012418 | miR-363-3p | 10396977 | 11321273 | 440 | 602 | 42.32 | 53.1742 | 0.3293868 | 0.0002581 |
|  | ACY_012418 | miR-3645-5p | 10396977 | 11321273 | 0 | 542 | 0.01 | 47.8745 | 12.2250417 | 4.68E-154 |
|  | ACY_012418 | miR-365-2-5p | 10396977 | 11321273 | 71 | 160 | 6.8289 | 14.1327 | 1.049312 | 1.32E-07 |
|  | ACY_012418 | miR-365a-5p | 10396977 | 11321273 | 11 | 7 | 1.058 | 0.6183 | -0.77496072 | 0.2694896 |
|  | ACY_012418 | miR-365b-5p | 10396977 | 11321273 | 76 | 175 | 7.3098 | 15.4576 | 1.0804125 | 1.58E-08 |
|  | ACY_012418 | miR-3725 | 10396977 | 11321273 | 0 | 68 | 0.01 | 6.0064 | 9.23035675 | 6.01E-20 |
|  | ACY_012418 | miR-383 | 10396977 | 11321273 | 7 | 341 | 0.6733 | 30.1203 | 5.48334286 | 2.40E-85 |
|  | ACY_012418 | miR-3896-3p | 10396977 | 11321273 | 0 | 39 | 0.01 | 3.4448 | 8.42827641 | 9.63E-12 |
|  | ACY_012418 | miR-3963 | 10396977 | 11321273 | 92 | 27 | 8.8487 | 2.3849 | -1.89153674 | 5.82E-11 |
|  | ACY_012418 | miR-3966 | 10396977 | 11321273 | 2607 | 3097 | 250.746 | 273.5558 | 0.12560846 | 0.001045 |
|  | ACY_012418 | miR-3970 | 10396977 | 11321273 | 2 | 16 | 0.1924 | 1.4133 | 2.87688703 | 0.0013698 |
|  | ACY_012418 | miR-3971 | 10396977 | 11321273 | 59 | 48 | 5.6747 | 4.2398 | -0.42054791 | 0.1331362 |
|  | ACY_012418 | miR-4006b-5p | 10396977 | 11321273 | 14 | 39 | 1.3465 | 3.4448 | 1.35520599 | 0.0016078 |
|  | ACY_012418 | miR-4112-3p | 10396977 | 11321273 | 1 | 159 | 0.0962 | 14.0444 | 7.18974238 | 8.36E-44 |
|  | ACY_012418 | miR-4123-3p | 10396977 | 11321273 | 0 | 103 | 0.01 | 9.0979 | 9.82938977 | 7.52E-30 |
|  | ACY_012418 | miR-4130-3p | 10396977 | 11321273 | 209 | 228 | 20.102 | 20.1391 | 0.00266017 | 0.9868314 |
|  | ACY_012418 | miR-425* | 10396977 | 11321273 | 107 | 215 | 10.2915 | 18.9908 | 0.88384741 | 1.11E-07 |
|  | ACY_012418 | miR-425-3p | 10396977 | 11321273 | 106 | 211 | 10.1953 | 18.6375 | 0.87030412 | 2.10E-07 |
|  | ACY_012418 | miR-4257 | 10396977 | 11321273 | 0 | 20 | 0.01 | 1.7666 | 7.46483161 | 2.29E-06 |
|  | ACY_012418 | miR-4289 | 10396977 | 11321273 | 0 | 80 | 0.01 | 7.0663 | 9.46481119 | 2.42E-23 |
|  | ACY_012418 | miR-4299 | 10396977 | 11321273 | 1 | 7237 | 0.0962 | 639.2391 | 12.69803114 | 0 |
|  | ACY_012418 | miR-4301 | 10396977 | 11321273 | 744 | 723 | 71.5593 | 63.8621 | -0.16417928 | 0.0292758 |
|  | ACY_012418 | miR-4311 | 10396977 | 11321273 | 107 | 5 | 10.2915 | 0.4416 | -4.54256929 | 3.17E-28 |
|  | ACY_012418 | miR-4448 | 10396977 | 11321273 | 262 | 390 | 25.1996 | 34.4484 | 0.45103614 | 8.13E-05 |
|  | ACY_012418 | miR-455-5p | 10396977 | 11321273 | 85 | 91 | 8.1755 | 8.038 | -0.02447039 | 0.907397 |
|  | ACY_012418 | miR-499 | 10396977 | 11321273 | 65 | 155 | 6.2518 | 13.691 | 1.13088429 | 3.44E-08 |
|  | ACY_012418 | miR-5112 | 10396977 | 11321273 | 155 | 136 | 14.9082 | 12.0128 | -0.31153362 | 0.0657582 |
|  | ACY_012418 | miR-5125 | 10396977 | 11321273 | 7 | 41 | 0.6733 | 3.6215 | 2.42726601 | 1.56E-06 |
|  | ACY_012418 | miR-5405* | 10396977 | 11321273 | 0 | 110 | 0.01 | 9.7162 | 9.92424838 | 7.87E-32 |
|  | ACY_012418 | miR-5423 | 10396977 | 11321273 | 12 | 33 | 1.1542 | 2.9149 | 1.33655315 | 0.0041853 |
|  | ACY_012418 | miR-71 | 10396977 | 11321273 | 471 | 26 | 45.3016 | 2.2966 | -4.3019905 | 1.59E-115 |
|  | ACY_012418 | miR-718 | 10396977 | 11321273 | 0 | 4031 | 0.01 | 356.0554 | 15.11981411 | 0 |
|  | ACY_012418 | miR-762 | 10396977 | 11321273 | 101 | 208 | 9.7144 | 18.3725 | 0.91935115 | 6.78E-08 |
|  | ACY_012418 | miR-9 | 10396977 | 11321273 | 7 | 16 | 0.6733 | 1.4133 | 1.06974637 | 0.1000144 |
|  | ACY_012418 | miR-92a-3p | 10396977 | 11321273 | 2107 | 2852 | 202.6551 | 251.9151 | 0.31391112 | 2.81E-14 |
|  | ACY_012418 | miR-980 | 10396977 | 11321273 | 0 | 24 | 0.01 | 2.1199 | 7.7278524 | 1.69E-07 |
|  | ACY_012418 | miR-9a-5p | 10396977 | 11321273 | 6 | 13 | 0.5771 | 1.1483 | 0.99260637 | 0.1663184 |
| cs | ACY_012710 | let-7k | 10396977 | 11321273 | 2357 | 2234 | 226.7005 | 197.3276 | -0.20019481 | 2.59E-06 |
|  | ACY_012710 | miR-10 | 10396977 | 11321273 | 4913 | 3851 | 472.5412 | 340.1561 | -0.47424316 | 4.10E-53 |
|  | ACY_012710 | miR-10-5p | 10396977 | 11321273 | 4699 | 3690 | 451.9583 | 325.9351 | -0.47160494 | 2.23E-50 |
|  | ACY_012710 | miR-122* | 10396977 | 11321273 | 2222 | 3279 | 213.716 | 289.6317 | 0.43852459 | 7.34E-29 |
|  | ACY_012710 | miR-126 | 10396977 | 11321273 | 202 | 181 | 19.4287 | 15.9876 | -0.28123599 | 0.056581 |
|  | ACY_012710 | miR-129-1* | 10396977 | 11321273 | 18 | 32 | 1.7313 | 2.8265 | 0.70716096 | 0.0956412 |
|  | ACY_012710 | miR-1306 | 10396977 | 11321273 | 17 | 16 | 1.6351 | 1.4133 | -0.21031113 | 0.673622 |
|  | ACY_012710 | miR-1306-3p | 10396977 | 11321273 | 10 | 13 | 0.9618 | 1.1483 | 0.25569077 | 0.6887866 |
|  | ACY_012710 | miR-139* | 10396977 | 11321273 | 256 | 570 | 24.6225 | 50.3477 | 1.03194862 | 6.96E-23 |
|  | ACY_012710 | miR-139-3p | 10396977 | 11321273 | 254 | 565 | 24.4302 | 49.906 | 1.0305476 | 1.19E-22 |
|  | ACY_012710 | miR-143-5p | 10396977 | 11321273 | 775 | 883 | 74.5409 | 77.9948 | 0.0653457 | 0.3581566 |
|  | ACY_012710 | miR-145* | 10396977 | 11321273 | 140 | 101 | 13.4655 | 8.9213 | -0.59394194 | 0.0015056 |
|  | ACY_012710 | miR-1560* | 10396977 | 11321273 | 0 | 13 | 0.01 | 1.1483 | 6.84335579 | 0.0002188 |
|  | ACY_012710 | miR-1576 | 10396977 | 11321273 | 13 | 35 | 1.2504 | 3.0915 | 1.30591732 | 0.0037282 |
|  | ACY_012710 | miR-1589 | 10396977 | 11321273 | 0 | 1725 | 0.01 | 152.368 | 13.89527232 | 0 |
|  | ACY_012710 | miR-1649 | 10396977 | 11321273 | 0 | 141 | 0.01 | 12.4544 | 10.28243981 | 1.33E-40 |
|  | ACY_012710 | miR-1777b | 10396977 | 11321273 | 87 | 92 | 8.3678 | 8.1263 | -0.04224974 | 0.8419389 |
|  | ACY_012710 | miR-1790 | 10396977 | 11321273 | 4 | 38 | 0.3847 | 3.3565 | 3.12515191 | 1.34E-07 |
|  | ACY_012710 | miR-1796 | 10396977 | 11321273 | 0 | 17 | 0.01 | 1.5016 | 7.23035675 | 1.62E-05 |
|  | ACY_012710 | miR-181a-3p | 10396977 | 11321273 | 23 | 34 | 2.2122 | 3.0032 | 0.44101873 | 0.2618025 |
|  | ACY_012710 | miR-190 | 10396977 | 11321273 | 13 | 9 | 1.2504 | 0.795 | -0.65336292 | 0.2988086 |
|  | ACY_012710 | miR-190a | 10396977 | 11321273 | 13 | 9 | 1.2504 | 0.795 | -0.65336292 | 0.2988086 |
|  | ACY_012710 | miR-193b-5p | 10396977 | 11321273 | 5 | 16 | 0.4809 | 1.4133 | 1.55525891 | 0.0284058 |
|  | ACY_012710 | miR-1957 | 10396977 | 11321273 | 178 | 268 | 17.1204 | 23.6722 | 0.46747738 | 0.0007417 |
|  | ACY_012710 | miR-210* | 10396977 | 11321273 | 40 | 67 | 3.8473 | 5.9181 | 0.62128774 | 0.0300764 |
|  | ACY_012710 | miR-210-5p | 10396977 | 11321273 | 10 | 63 | 0.9618 | 5.5647 | 2.53249508 | 8.48E-10 |
|  | ACY_012710 | miR-212* | 10396977 | 11321273 | 26 | 32 | 2.5007 | 2.8265 | 0.1766847 | 0.6508082 |
|  | ACY_012710 | miR-212-5p | 10396977 | 11321273 | 26 | 36 | 2.5007 | 3.1799 | 0.3466494 | 0.3562982 |
|  | ACY_012710 | miR-2131 | 10396977 | 11321273 | 21 | 21 | 2.0198 | 1.8549 | -0.12287103 | 0.7788413 |
|  | ACY_012710 | miR-22* | 10396977 | 11321273 | 83 | 90 | 7.9831 | 7.9496 | -0.00606681 | 0.9746255 |
|  | ACY_012710 | miR-221* | 10396977 | 11321273 | 827 | 1770 | 79.5424 | 156.3428 | 0.97491678 | 1.13E-61 |
|  | ACY_012710 | miR-221-5p | 10396977 | 11321273 | 857 | 1805 | 82.4278 | 159.4344 | 0.95176005 | 1.82E-60 |
|  | ACY_012710 | miR-222b | 10396977 | 11321273 | 24 | 25 | 2.3084 | 2.2082 | -0.06402239 | 0.8718061 |
|  | ACY_012710 | miR-22-3p | 10396977 | 11321273 | 1314 | 1246 | 126.3829 | 110.0583 | -0.19953333 | 0.0004675 |
|  | ACY_012710 | miR-2270 | 10396977 | 11321273 | 0 | 115 | 0.01 | 10.1579 | 9.98838646 | 3.03E-33 |
|  | ACY_012710 | miR-2360 | 10396977 | 11321273 | 0 | 147 | 0.01 | 12.9844 | 10.34256363 | 2.68E-42 |
|  | ACY_012710 | miR-24c | 10396977 | 11321273 | 0 | 21 | 0.01 | 1.8549 | 7.5351976 | 1.19E-06 |
|  | ACY_012710 | miR-27a-3p | 10396977 | 11321273 | 1260 | 1617 | 121.1891 | 142.8285 | 0.23702394 | 1.17E-05 |
|  | ACY_012710 | miR-27b-3p | 10396977 | 11321273 | 1246 | 1608 | 119.8425 | 142.0335 | 0.24509162 | 6.40E-06 |
|  | ACY_012710 | miR-27c | 10396977 | 11321273 | 1081 | 1411 | 103.9725 | 124.6326 | 0.26147949 | 6.90E-06 |
|  | ACY_012710 | miR-27d | 10396977 | 11321273 | 1162 | 1410 | 111.7633 | 124.5443 | 0.15621247 | 0.0062337 |
|  | ACY_012710 | miR-27d-3p | 10396977 | 11321273 | 1481 | 1840 | 142.4453 | 162.5259 | 0.19026162 | 0.0001545 |
|  | ACY_012710 | miR-2861 | 10396977 | 11321273 | 71 | 226 | 6.8289 | 19.9624 | 1.54756007 | 2.15E-17 |
|  | ACY_012710 | miR-2881 | 10396977 | 11321273 | 25 | 67 | 2.4045 | 5.9181 | 1.29939715 | 5.64E-05 |
|  | ACY_012710 | miR-2890 | 10396977 | 11321273 | 606 | 1149 | 58.2862 | 101.4904 | 0.80011702 | 1.37E-29 |
|  | ACY_012710 | miR-2900 | 10396977 | 11321273 | 75 | 145 | 7.2136 | 12.8077 | 0.82822009 | 3.79E-05 |
|  | ACY_012710 | miR-2954* | 10396977 | 11321273 | 126 | 243 | 12.1189 | 21.464 | 0.8246602 | 1.03E-07 |
|  | ACY_012710 | miR-309b-3p | 10396977 | 11321273 | 0 | 749 | 0.01 | 66.1586 | 12.69171299 | 1.27E-212 |
|  | ACY_012710 | miR-30a | 10396977 | 11321273 | 9863 | 6083 | 948.6411 | 537.3071 | -0.82011547 | 5.51E-275 |
|  | ACY_012710 | miR-30a-3p | 10396977 | 11321273 | 481 | 434 | 46.2634 | 38.3349 | -0.27121288 | 0.0044822 |
|  | ACY_012710 | miR-30a-5p | 10396977 | 11321273 | 9813 | 6031 | 943.832 | 532.7139 | -0.82516916 | 1.75E-276 |
|  | ACY_012710 | miR-30b | 10396977 | 11321273 | 30 | 3 | 2.8855 | 0.265 | -3.44475707 | 2.24E-07 |
|  | ACY_012710 | miR-30b-5p | 10396977 | 11321273 | 29 | 9 | 2.7893 | 0.795 | -1.81087634 | 0.0004084 |
|  | ACY_012710 | miR-30c | 10396977 | 11321273 | 364 | 245 | 35.0102 | 21.6407 | -0.69402814 | 4.04E-09 |
|  | ACY_012710 | miR-30c-5p | 10396977 | 11321273 | 363 | 244 | 34.914 | 21.5523 | -0.69596381 | 3.89E-09 |
|  | ACY_012710 | miR-30d | 10396977 | 11321273 | 14165 | 7647 | 1362.4152 | 675.4541 | -1.0122368 | 0 |
|  | ACY_012710 | miR-30d-5p | 10396977 | 11321273 | 14268 | 7618 | 1372.322 | 672.8925 | -1.02817109 | 0 |
|  | ACY_012710 | miR-30e | 10396977 | 11321273 | 1784 | 1590 | 171.5883 | 140.4436 | -0.2889603 | 6.11E-09 |
|  | ACY_012710 | miR-30e-5p | 10396977 | 11321273 | 1712 | 1525 | 164.6632 | 134.7022 | -0.28974475 | 1.13E-08 |
|  | ACY_012710 | miR-3195 | 10396977 | 11321273 | 7 | 33 | 0.6733 | 2.9149 | 2.11412502 | 7.86E-05 |
|  | ACY_012710 | miR-3201 | 10396977 | 11321273 | 1763 | 3169 | 169.5685 | 279.9155 | 0.72312319 | 3.11E-66 |
|  | ACY_012710 | miR-32 | 10396977 | 11321273 | 15 | 22 | 1.4427 | 1.9432 | 0.42966306 | 0.3827136 |
|  | ACY_012710 | miR-32-5p | 10396977 | 11321273 | 0 | 20 | 0.01 | 1.7666 | 7.46483161 | 2.29E-06 |
|  | ACY_012710 | miR-33-2* | 10396977 | 11321273 | 5 | 15 | 0.4809 | 1.3249 | 1.46207464 | 0.0429669 |
|  | ACY_012710 | miR-3483-5p | 10396977 | 11321273 | 14 | 23 | 1.3465 | 2.0316 | 0.59340215 | 0.2298126 |
|  | ACY_012710 | miR-34a | 10396977 | 11321273 | 18 | 23 | 1.7313 | 2.0316 | 0.23076064 | 0.6215171 |
|  | ACY_012710 | miR-363 | 10396977 | 11321273 | 468 | 633 | 45.0131 | 55.9124 | 0.31282335 | 0.0003583 |
|  | ACY_012710 | miR-365a-3p | 10396977 | 11321273 | 237 | 474 | 22.7951 | 41.8681 | 0.87712771 | 4.43E-15 |
|  | ACY_012710 | miR-378f | 10396977 | 11321273 | 1 | 84 | 0.0962 | 7.4197 | 6.26918015 | 7.45E-23 |
|  | ACY_012710 | miR-3885-5p | 10396977 | 11321273 | 38 | 23 | 3.6549 | 2.0316 | -0.84721555 | 0.0246346 |
|  | ACY_012710 | miR-3918 | 10396977 | 11321273 | 10 | 21 | 0.9618 | 1.8549 | 0.94753258 | 0.0856033 |
|  | ACY_012710 | miR-3960 | 10396977 | 11321273 | 33 | 81 | 3.174 | 7.1547 | 1.17258915 | 4.28E-05 |
|  | ACY_012710 | miR-3965 | 10396977 | 11321273 | 1 | 13 | 0.0962 | 1.1483 | 3.5773189 | 0.0016851 |
|  | ACY_012710 | miR-3967 | 10396977 | 11321273 | 364 | 291 | 35.0102 | 25.7038 | -0.44579364 | 8.05E-05 |
|  | ACY_012710 | miR-3968 | 10396977 | 11321273 | 77 | 67 | 7.406 | 5.9181 | -0.32356048 | 0.1790087 |
|  | ACY_012710 | miR-4000i-5p | 10396977 | 11321273 | 94 | 94 | 9.0411 | 8.303 | -0.12286561 | 0.5577931 |
|  | ACY_012710 | miR-4117-5p | 10396977 | 11321273 | 0 | 59 | 0.01 | 5.2114 | 9.02552718 | 2.11E-17 |
|  | ACY_012710 | miR-4171-5p | 10396977 | 11321273 | 132 | 91 | 12.696 | 8.038 | -0.65946555 | 0.0007177 |
|  | ACY_012710 | miR-4298 | 10396977 | 11321273 | 0 | 68 | 0.01 | 6.0064 | 9.23035675 | 6.01E-20 |
|  | ACY_012710 | miR-4332 | 10396977 | 11321273 | 175 | 335 | 16.8318 | 29.5903 | 0.81393486 | 6.29E-10 |
|  | ACY_012710 | miR-4434 | 10396977 | 11321273 | 0 | 33 | 0.01 | 2.9149 | 8.18730258 | 4.80E-10 |
|  | ACY_012710 | miR-4451 | 10396977 | 11321273 | 26 | 30 | 2.5007 | 2.6499 | 0.08360592 | 0.8363391 |
|  | ACY_012710 | miR-4459 | 10396977 | 11321273 | 0 | 442 | 0.01 | 39.0415 | 11.93079277 | 9.19E-126 |
|  | ACY_012710 | miR-4488 | 10396977 | 11321273 | 13 | 83 | 1.2504 | 7.3313 | 2.55167936 | 1.32E-12 |
|  | ACY_012710 | miR-4497 | 10396977 | 11321273 | 3746 | 5608 | 360.297 | 495.3507 | 0.45926365 | 2.86E-52 |
|  | ACY_012710 | miR-4530 | 10396977 | 11321273 | 3 | 96 | 0.2885 | 8.4796 | 4.87735299 | 1.32E-23 |
|  | ACY_012710 | miR-455 | 10396977 | 11321273 | 75 | 77 | 7.2136 | 6.8014 | -0.08488769 | 0.7144518 |
|  | ACY_012710 | miR-455-3p | 10396977 | 11321273 | 35 | 34 | 3.3664 | 3.0032 | -0.16470605 | 0.633435 |
|  | ACY_012710 | miR-4651 | 10396977 | 11321273 | 440 | 472 | 42.32 | 41.6914 | -0.02158981 | 0.8200122 |
|  | ACY_012710 | miR-4660 | 10396977 | 11321273 | 0 | 44 | 0.01 | 3.8865 | 8.60232771 | 3.71E-13 |
|  | ACY_012710 | miR-4674 | 10396977 | 11321273 | 2180 | 5383 | 209.6763 | 475.4766 | 1.18121054 | 4.14E-250 |
|  | ACY_012710 | miR-4948-5p | 10396977 | 11321273 | 14 | 37 | 1.3465 | 3.2682 | 1.27928204 | 0.003311 |
|  | ACY_012710 | miR-5097 | 10396977 | 11321273 | 1395 | 2215 | 134.1736 | 195.6494 | 0.54416985 | 6.42E-29 |
|  | ACY_012710 | miR-5108 | 10396977 | 11321273 | 36 | 19 | 3.4625 | 1.6783 | -1.04481345 | 0.0092111 |
|  | ACY_012710 | miR-5447 | 10396977 | 11321273 | 38 | 40 | 3.6549 | 3.5332 | -0.04885652 | 0.8769811 |
|  | ACY_012710 | miR-5584-5p | 10396977 | 11321273 | 0 | 30 | 0.01 | 2.6499 | 8.04979411 | 3.39E-09 |
|  | ACY_012710 | miR-573 | 10396977 | 11321273 | 19 | 25 | 1.8275 | 2.2082 | 0.27299944 | 0.543593 |
|  | ACY_012710 | miR-664-5p | 10396977 | 11321273 | 0 | 38 | 0.01 | 3.3565 | 8.39081383 | 1.85E-11 |
|  | ACY_012710 | miR-709 | 10396977 | 11321273 | 105 | 795 | 10.0991 | 70.2218 | 2.79769225 | 1.09E-119 |
