# Supplementary material for: The goose genome sequence leads to insights into the evolution of waterfowl and susceptibility to fatty liver
Source: Genome Biol. 2015 May 6;16(1):89. doi: 10.1186/s13059-015-0652-y (PMC4419397; doi:10.1186/s13059-015-0652-y)
Supplement: Additional file 2: Figure S1. — GC content distributions for various bird genomes. Figure S2. Venn diagram of orthologous genes among five species. Figure S3. Whole-genome alignment of goose and chicken sequences. Figure S4. Chromosomal rearrangement between geese and chickens. Figure S5. Orthologous information of nine genomes. Figure S6. Phylogenetic tree constructed with orthologous genes. Figure S7. Differences between the chicken and goose MHC gene regions. Figure S8. Phylogenetic tree of the RIG-I gene in various species. Figure S9. Comparison of RIG-I gene structures in seven avian species. Figure S10. Phylogenetic tree of the Mx gene in seven species and comparison of gene structures. Figure S11. Single amino-acid changes in the Mx gene in four birds. [file 13059_2015_652_MOESM2_ESM.doc]

**Additional file 2**

**
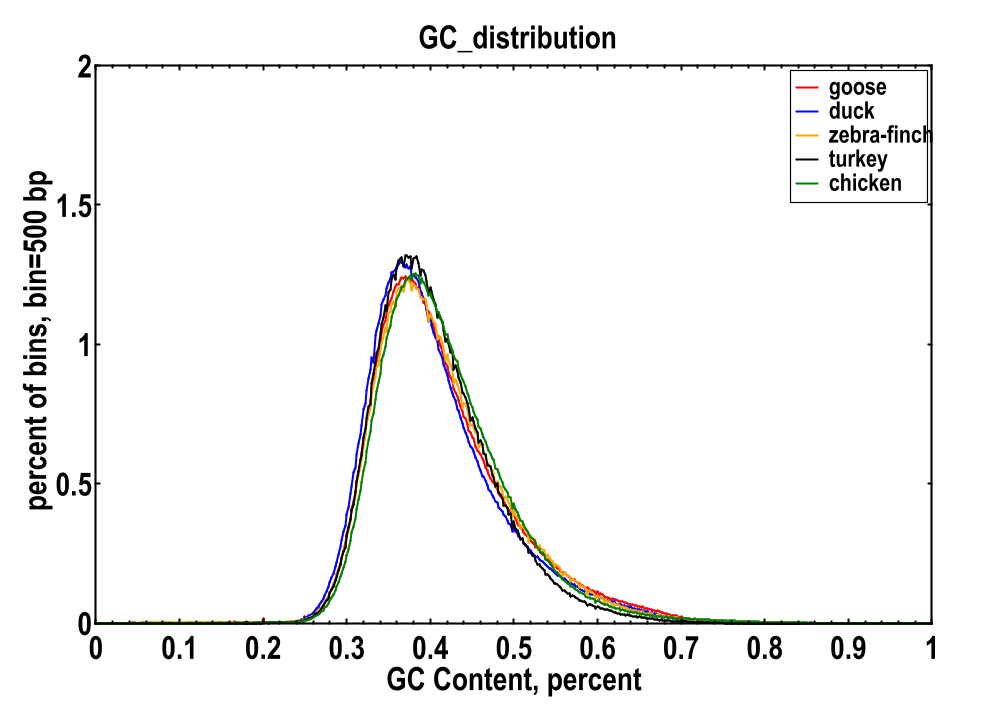
**

**Figure S1. GC content distributions for various bird genomes.** The x-axis represents the GC content and the y-axis represents the proportion of bins divided by the total windows. We used 500-bp bins (with 250-bp overlaps) sliding along the genome. Each of the bird species compared here have similar GC content distribution curves.


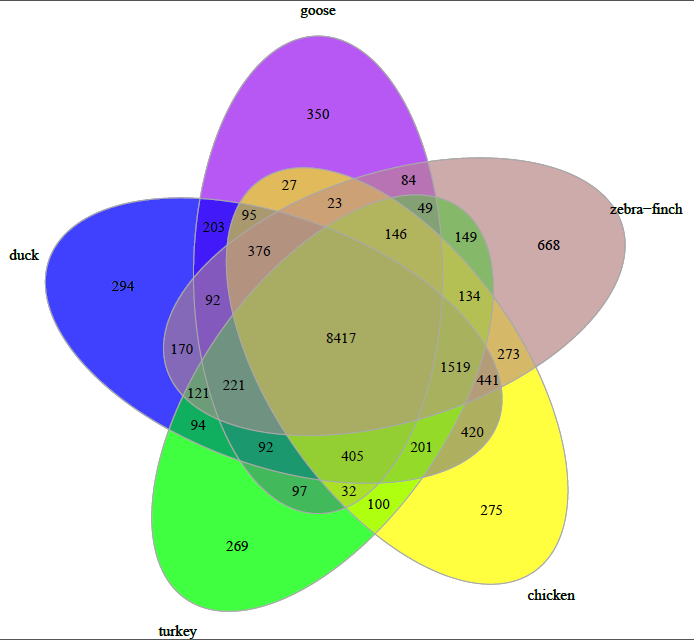


**Figure S2. Venn diagram of orthologous genes among five species.** The five species analyzed included goose, duck, chicken, turkey, and zebra finch.


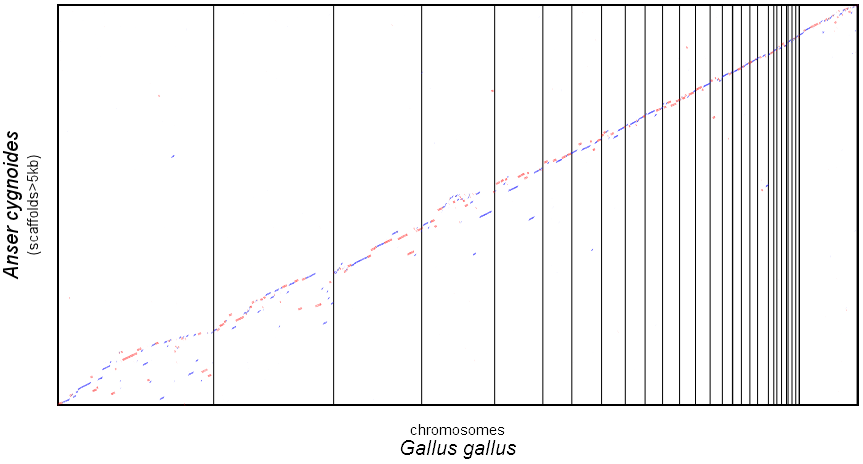


**Figure S3. Whole-genome alignment of goose and chicken sequences.** All scaffolds longer than 5 Kb in chicken were included in the figure. Alignments to both the plus strand (red) and minus strand (blue) are indicated.


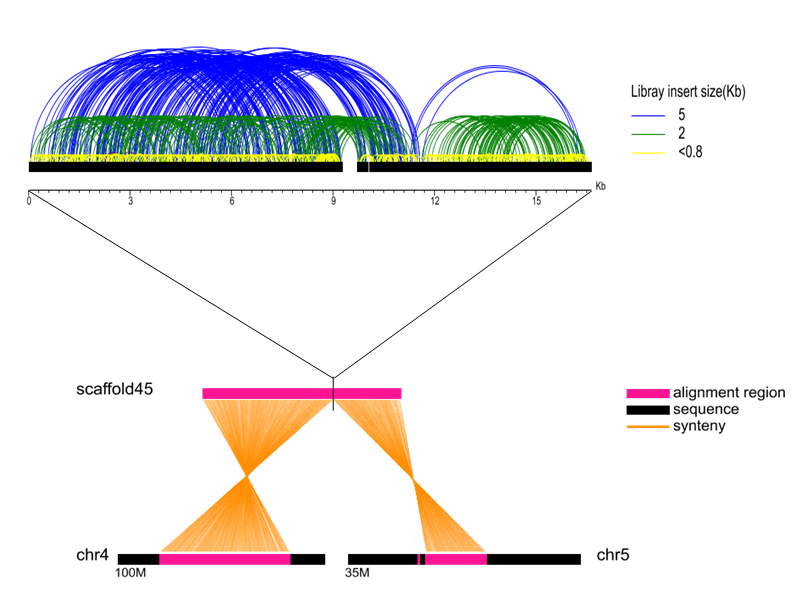


**Figure S4. Chromosomal rearrangement between geese and chickens.** Scaffold 45 is a goose genome sequence fragment, and chr4 and chr5 are chicken chromosomes. Our data suggest that the chicken genome underwent a chromosomal rearrangement.

**Figure** **S5. Orthologous information from 17,239 gene families of nine genomes.**

Figure S6. Phylogenetic tree constructed with orthologous genes on 4-fold degenerate sites. The branch length represents the neutral divergence rate.


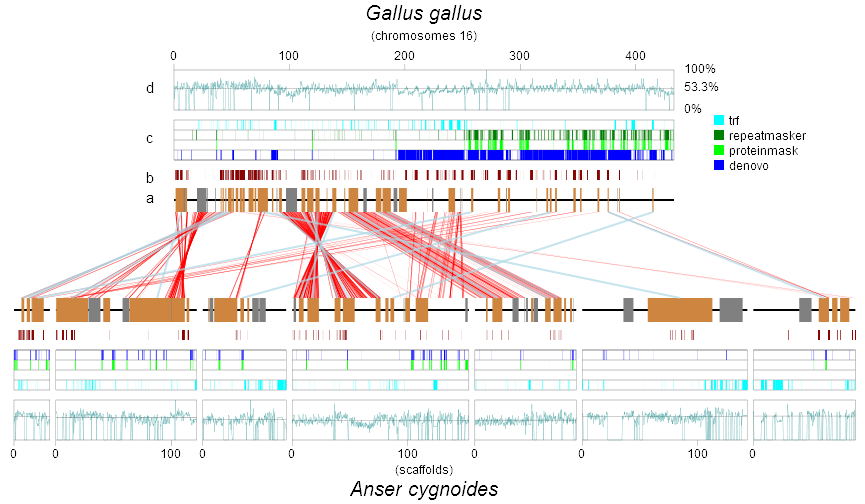


Figure S7. Differences between the chicken and goose MHC gene regions. (a) Chicken MHC protein sequences were downloaded and blastp analysis was performed (E-value cutoff 1e-5) with the goose genes. Gray-yellow regions indicate cases where genes have been mapped, and gray regions indicate unmapped genes. Genes encoding chicken the MHC protein are primarily localized to chr16, while the homologous goose genes are mainly distributed on seven scaffolds. The units of the coordinates are kb for each scaffold shown (scaffold 544, scaffold 558, scaffold 518, scaffold 315, scaffold 400, scaffold 478, and scaffold 565). (b) The downloaded MHC proteins were mapped onto chicken chr16 and the seven goose scaffolds. Alignment relationships are represented by the red line. (c) Denovo, proteinmask, RepeatMasker, and trf regions are represented by different colors, as shown.

**Figure S8. Phylogenetic tree of the RIG-I gene in various species.** chicken_ENSGALP00000018045, peregrine_CCG010430.2, saker_CCG008297.1, and turkey_ENSMGAP00000011342 are phylogenetically distant from the RIG-I genes shown, the phylogenetic relationships of which are in accordance with the published literature. The phylogenetic tree indicates that the four genes mentioned above are distinct from the other genes shown, suggesting that the RIG-I gene is absent in chickens, turkeys, peregrines, and sakers.

**Figure S9. Comparison of RIG-I gene structures in seven avian species.**

**Figure S10. Phylogenetic tree of the Mx gene in seven species and comparison of gene structures.** The phylogenetic tree was primarily constructed using all of the loci of the Mx protein sequences in seven species with MUSCLE software and secondarily constructed tree with PhyML. **(a)** The high scores and branches of phylogenetic tree indicate the accuracy of the gene relationships among the different species represented. In chickens, Mx protein inactivation or weak viral resistance may result from mutations in key nucleotides in the Mx gene, which govern antiviral activity. **(b)** Gene structure among the four avian Mx genes, showing that the chicken and turkey genes are structurally similar, while those of geese and zebra finches have fewer exons.

**Figure S11. Single amino-acid changes in the Mx gene in the goose, zebra finch, turkey, and chicken genes.**
